# Supplementary material for: Common evolutionary origin of acoustic communication in choanate vertebrates
Source: Nat Commun. 2022 Oct 25;13:6089. doi: 10.1038/s41467-022-33741-8 (PMC9596459; doi:10.1038/s41467-022-33741-8)
Supplement: Supplementary file 1 — Supplementary Information [file 41467_2022_33741_MOESM1_ESM.pdf]

Supplementary Note 1 – Data acquisition + PRISMA workflow diagram

We used *Google scholar* and *Web of Science* databases to perform an electronic search using different combinations of the words “acoustic communication”, “call”, “vocal communication”, “vocalization”, “song”, and “sound”, in association with the species’ name and any other superior taxonomic rank. Studies from all dates were considered. We did not limit the search by language or date. Study eligibility was assessed by one investigator. A secondary search was conducted on the reference list of these publications as well as on the list of publications that have cited the previous accessed one.
We included 78 publications (i.e., papers, book chapters, complete books, and dissertations).
We used the PRISMA checklist (Moher et al. 2011) as a guide for quality reporting of our review.
Data from captivity was considered as the characteristics of interest are mostly supposed to be inheritable and we considered possible bias to be irrelevant. Selected data is available in Supplementary material 1.

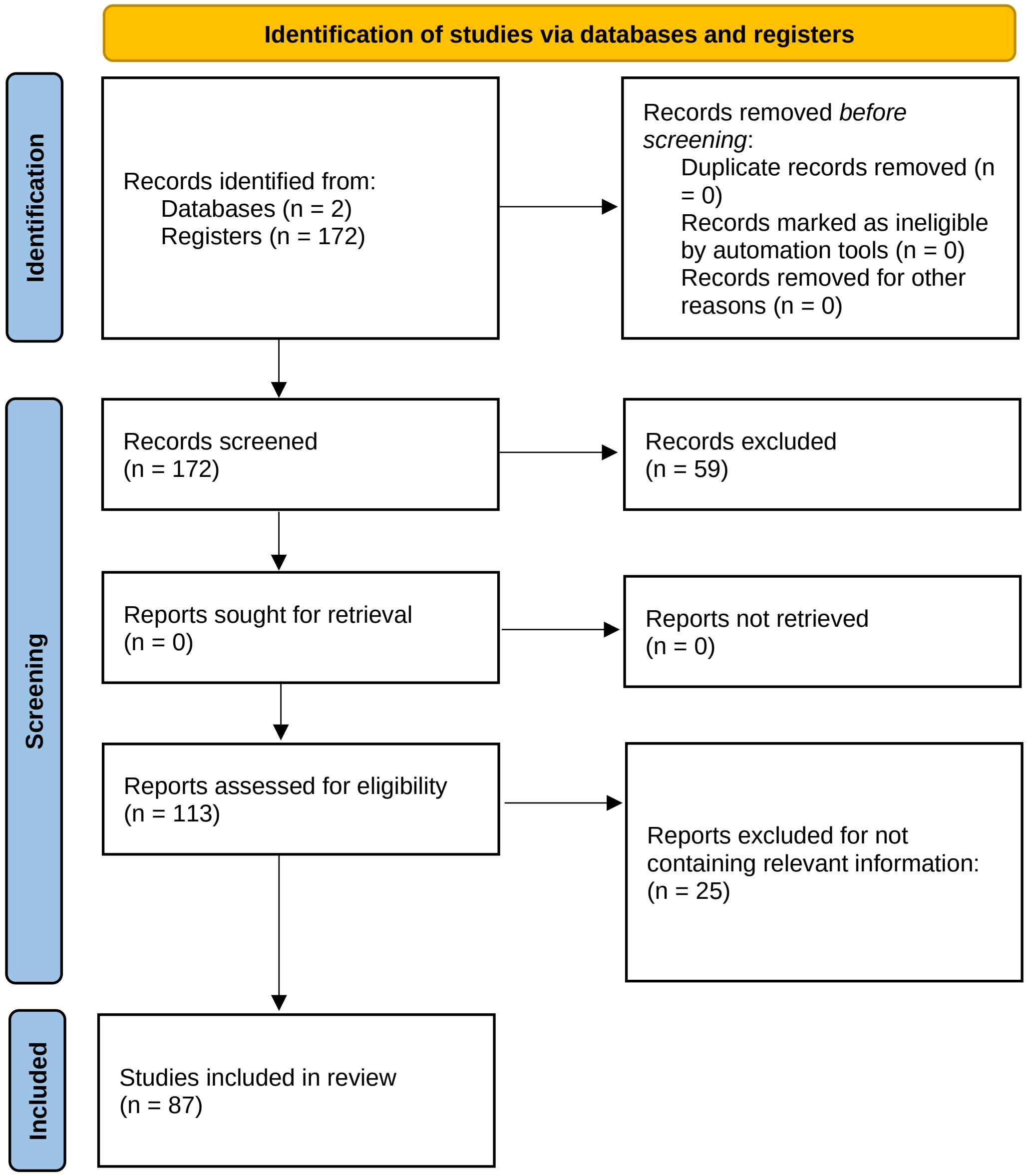

References

1. Giles, J.C., Davis, J.A., McCauley, R.D. & Kuchling, G. Voice of the turtle: the underwater acoustic repertoire of the long-necked freshwater turtle, *Chelodina oblonga*. *The Journal of the Acoustical Society of America*, **126(1),** pp.434-443 (2009).
2. Allen, E.R. Sounds produced by the Suwannee Terrapin. *Copeia*, **1950(1),** pp.62-62 (1950).
3. Auffenberg, W. Notes on the courtship of the land tortoise *Geochelone travancorica* (Boulenger). *J Bombay Nat Hist Soc* **61,** pp.247–253(1964).
4. Auffenberg, W. Sex and species discrimination in two sympatric South American tortoises. *Copeia* **1965,** 335–342 (1965).
5. Barton, B.S. Sorne accounts of Siren *lacertina* and other species of the same genus of amphibious animals, Private printing. Philadelphia, PA, 84 (1808).
6. Bedi, A., Bedi, V., Nag, S. and Suyesh, R. Wild observations of the reproductive behaviour and first evidence of vocalization in Crocodile newt *Tylototriton himalayanus* (Caudata: Salamandridae) from the Himalayan biodiversity hotspot in Eastern India. Salamandra, **57(1),** pp.65-74 (2021).
7. Bell, B.D. Observations on the ecology and reproduction of the New Zealand Leiopelmid frogs. *Herpetologica*, pp.340-354 (1978).
8. Campbell, H.W. Stop, look, and listen. Acoustic behaviour of turtles. *J Int Turt Tortoise Soc* **1,** 13–44 (1967).
9. Campbell, H.W., Evans, W.E. Sound production in two species of tortoises. *Herpetologica* **23,** 204–209 (1967).
10. Campbell, H.W. & Evans, W.E. Observations on the vocal behavior of chelonians. *Herpetologica*, pp.277-280 (1972).
11. Carr, A.F.J. Handbook of turtles. Comstock, Ithaca (1952).
12. Cohen, M.A. Russian tortoise, *Testudo horsfieldii*. *Tortuga Gaz* **30(4),** (1994)
13. Colafrancesco, K.C. & Gridi-Papp, M. Vocal sound production and acoustic communication in amphibians and reptiles. In Vertebrate sound production and acoustic communication (pp. 51-82). *Springer*, Cham (2016).

14. Combs, S.A. Nest building and egg-laying in the wood turtle. *Herp Bull. NY Herpetol. Soc*., **7,** pp.28-29 (1971).
15. Connor, M.J. & Wheeler, V. The Chinese box turtle, *Cistoclemmys flavomarginata* Gray 1863. *Tortuga Gaz* **34,** 1–7 (1998).
16. Cook, S.L. & Forrest, T.G. Sounds produced by nesting leatherback sea turtles (*Dermochelys coriacea*). *Herpetological Review*, **36(4),** pp.387-389 (2005).
17. Cope, E.D. Third contribution to the herpetology of tropical America. *Proc Natl Acad Sci USA* **173,** 185–196 (1865).
18. Cope, E.D. The Batrachia of North America (Vol. 34). *US Government Printing Office* (1889).
19. Davis, M. Aspects of the social and spatial experience of eastern box turtles, *Terrapene carolina carolina* (Doctoral dissertation, The University of Tennessee) (1981).
20. Donoso-Barros, R. Reptiles de Chile. Universidad de Chile, Santiago, Chile. cxlvi + 458 pp (1966).
21. Duellman, W.E. & Trueb, L. Biology of amphibians. JHU press (1994).
22. Ernst, C.H. & Barbour, R.W. Turtles of the world. *Smithsonian Institution Press*, Washington, DC (1989).
23. Evans, W.E. The reproductive behaviour of the giant tortoises *T. vicinia* and *T. vandenberghii*. *Anat Rec* **105,** 579 (1949).
24. Ferrara, C.R., Vogt, R.C. and Sousa-Lima, R.S. Turtle vocalizations as the first evidence of posthatching parental care in chelonians. *Journal of Comparative Psychology*, **127(1),** p.24 (2013).
25. Ferrara, C.R., Mortimer, J.A. & Vogt, R.C. First evidence that hatchlings of *Chelonia mydas* emit sounds. *Copeia*, **2014(2),** pp.245-247 (2014).
26. Ferrara, C.R., Vogt, R.C., Giles, J.C. & Kuchling, G. Chelonian vocal communication. In Biocommunication of animals (pp. 261-274). *Springer*, Dordrecht (2014).
27. Ferrara, C.R., Vogt, R.C. & Pappas, M. *Emydoidea Blandingii* (blanding’s Turtle). Vocalizations. Volume 49, Pags. 526-527 (2018).

28. Ferrara, C.R., Vogt, R.C., Eisemberg, C.C. & Doody, J.S. First evidence of the pig-nosed turtle (*Carettochelys insculpta*) vocalizing underwater. *Copeia*, **105(1),** pp.29-32 (2017).
29. Ferrara, C.R., Vogt, R.C., Sousa-Lima, R.S., Lenz, A. & Morales-Mávil, J.E. Sound Communication in Embryos and Hatchlings of *Lepidochelys kempii*. *Chelonian Conservation and Biology*: Celebrating 25 Years as the World's Turtle and Tortoise Journal, **18(2),** pp.279-283 (2019).
30. Fitze, P.S. Lagartija cenicienta–*Psammodromus hispanicus* Fitzinger, 1826 (2012).
31. Flower, S.S. Notes on a second collection of reptiles made in the Malay Peninsula and Siam, from November 1896 to September 1898, with a list of species recorded from those countries. *Proc Zool Soc (Lond)* **1899,** 600–696 (1899).
32. Franzen, M. & Glaw, F. Distress calls of *Mertensiella luschani finikensis* from Turkey (Amphibia: Salamandridae). *Zoology in the Middle East*, **19(1),** pp.27-32 (1999).
33. Frazier, J. & Peters, G. The call of the Aldabra tortoise (*Geochelone gigantea*) (Reptilia, Testudinidae). *Amphib-Reptil* **2,** 165–179 (1982).
34. Galeotti, P., Sacchi, R., Fasola. M., & Ballasina, D. Do mounting in tortoises have a communication function? A comparative analysis. *Herpetol J* **15,** 61–71 (2005a).
35. Gans, C. & Wever, E.G. Ear and hearing in *Sphenodon punctatus*. *Proceedings of the National Academy of Sciences*, **73(11),** pp.4244-4246 (1976).
36. Geller, G.A. and Casper, G.S., 2019. Late term embryos and hatchlings of Ouachita Map Turtles (*Graptemys ouachitensis*) make sounds within the nest. *Herpetological Review*, **50(3),** pp.449-452.
37. Geyer, H. "Uber Lautausserungen der Molche," Blatter fur Aquar.-Terror. *Kunde,* **39,** 27-28 (1927).
38. Green, D.M. Antipredator behaviour and skin glands in the New Zealand native frogs, genus *Leiopelma*. *New Zealand journal of zoology*, **15(1),** pp.39-45 (1988).
39. Grubb, P. Comparative notes on the behaviour of *Geochelone sulcata*. *Herpetologica* **27**:328–332 (1971).
40. Gunther, A. Reptiles of British India. Robert Hardwicks, London (1864).

41. Hine, M. Notes on the marginated tortoise (*Testudo marginata*) in Greece and captivity. *Bull Br Herpetol Soc* **5**:35–38 (1982).
42. Hoofien, J.H. The voices of snake and tortoises. *Israel J Zool* **20**:148 (1971).
43. Hubáček, J., Šugerková, M. & Gvoždík, L. Underwater sound production varies within not between species in sympatric newts. *PeerJ*, **7,** p.e6649 (2019).
44. Jackson, C.G. & Awbrey, F.T. Mating bellows of the Galapagos Tortoise, *Geochelone elephantopus*. *Herpetologica* **34**:134–136 (1972).
45. Johnson, M.A., Cook, E.G. & Kircher, B.K. Phylogeny and Ontogeny of Display Behavior. Behavior of lizards: evolutionary and mechanistic perspectives, p.259 (2019).
46. Kastle, W. Verhaltensstudien an Taggeckonen der Gattungen *Lygodactylus* und *Phelsuma*. *Z. Tierpsychol*. **21**:486-507 (1964).
47. Kaufmann, J.H. The social behavior of wood turtles, *Clemmys insculpta*, in central Pennsylvania. *Herpetological Monographs*, pp.1-25 (1992).
48. Kirkpatrick, D.T. African hingeback tortoises of the genus *Kinixys*. *Reptile Amphib Mag* **54**:32–37 (1998).
49. Klemmer, K. Die echten Eidechsen, p. 285- 307. In Grzrmeks Tierleben. Vol. 6. Kindler, Munich (1971)
50. Labra, A. Sistemas de comunicación en reptiles. *Herpetología de Chile*, pp.547-577 (2008).
51. Largen, M.J., Morris, P.A. & Yalden, D.W. Observations on the caecilian *Geotrypetes grandisonae* Taylor (Amphibia, Gymnophiona) from Ethiopia: pubblicazioni del centro di studio per la faunistica ed ecologia tropicali del CNR: LX. Monitore Zoologico Italiano. *Supplemento*, **4(1),** pp.185-205 (1972).
52. Lenz, A. Vocalizaciones perinatales de la tortuga caguama (*Caretta caretta*) (Doctoral dissertation, MS Thesis, Instituto de Neuroetologia, Universidad Veracruzana, Jalapa, Veracruz, Mexico) (2017).
53. Lukanov, S. First record of underwater sound produced by the Balkan crested newt (*Triturus ivanbureschi*). *Acta Herpetologica*, **15(1),** pp.15-20 (2020).

54. Maslin, T.P. The production of sound in caudate Amphibia. *Series in Biology*, **1,** pp.29-45 (1950).
55. McCormick, B. The elongated tortoise, *Indotestudo elongata*. *Tortuga Gaz* **28**:1–3 (1992).
56. McKeown, S., Meier, D.E. & Juvik, J.O. The management and breeding of the Asian Forest Tortoise (*Manouria emys*) in captivity. In: Beaman, K.R., Caporaso, F., McKeown, S. & Graff, M.D. (eds) Proceedings of the first international symposium on turtles and tortoises: conservation and captive husbandry. *California Turtle & Tortoise Club*, Van Nuys, Los Angeles, CA, pp 138–159 (1990).
57. Mertens, R. Die Warn-und Droh-Reaktionen der Reptilien (1946).
58. Milton, T.H. Structure and Social Significance of Vocalization by *Anolis Grahami* Grahami (Sauria: Iguanidae) (Doctoral dissertation, Virginia Polytechnic Institute and State University) (1974).
59. Monteiro, C.C., Carmo, H.M., Santos, A.J., Corso, G. & Sousa-Lima, R.S. First record of bioacoustic emission in embryos and hatchlings of hawksbill sea turtles (*Eretmochelys imbricata*). *Chelonian Conservation and Biology*: Celebrating 25 Years as the World's Turtle and Tortoise Journal, **18(2),** pp.273-278 (2019).
60. Morris, L. Western hinge-back tortoise, *Kinixys belliana nogueyi*. *Tortuga Gaz* **30**:1–3 (1974).
61. Mrosovsky, N. Spectrographs of the sounds of leatherback turtles. *Herpetologica*, pp.256-258 (1972).
62. Murphy, J.B. & Mitchell, L.A. Ritualized combat behavior of the pygmy mulga monitor lizard, *Varanus gilleni* (Sauria: Varanidae). *Herpetologica*, pp.90-97 (1974).
63. Palmer, M. The speckled tortoise, *Homopus signatus*, in captivity. *Tortuga Gaz* **30**:1–5 (1994).
64. Pope, C.H. Turtles of the United States & Canada. AA Knopf, New York (1939).
65. Regal, P.J., Greenberg, N. & McLean, P. Behavior and neurology of lizards (1978).
66. Rhen, T. & Crews, D. Organization and Activation of Sexual and Agonistic Behavior in the Leopard Gecko, *Eublepharis macularius*. *Neuroendocrinology*, **71(4),** pp.252-261 (2000).

67. Ritter, W.E. & Miller, L. A contribution to the life history of *Autodax lugubris* Hallow., a Californian salamander. *The American Naturalist*, **33(393),** pp.691-704 (1899).
68. Rothblum, L.M., Watkins, J.W. & Jenssen, T.A. A learning paradigm and the behavioral demonstration of audition for the lizard *Anolis grahami*. *Copeia*, pp.490-494 (1979).
69. Santos, M.T.T., Barata, I.M., Ferreira, R.B., Haddad, C.F., Gridi-Papp, M. & de Carvalho, T.R. Complex acoustic signals in *Crossodactylodes* (Leptodactylidae, Paratelmatobiinae): a frog genus historically regarded as voiceless. *Bioacoustics*, pp.1-16 (2021).
70. Simons, R. Tonausserungen des Scheltopusik (Pseudopus Pallasii). *Zool. Garten* **18**:230-233 (1877).
71. Sonneman, N. Notes on *Delma fraseri* in the north-east of Victoria. *Herpetofauna* (Sydney), **7,** p.15 (1974).
72. Stephenson, B. & Verrell, P. Courtship and mating of the tailed frog (*Ascaphus truei*). *Journal of Zoology*, **259(1),** pp.15-22 (2003).
73. Suthers, R.A., Fitch, W.T., Fay, R.R. & Popper, A.N. Vertebrate sound production and acoustic communication. *Springer International Publishing* (2016).
74. Thurow, G.R. & Gould, H.J. Sound production in a caecilian. *Herpetologica*, pp.234-237 (1977).
75. Wclt, [Professor, ed.j 1927 Editorial Addendum to H Geyer, " liber Lautausserungen der Molche," Blatter fur Aquar.-Terrar. Kunde, 39: 28 – in Maslin (1950).
76. Werner, F. Die Lurche und Kriechtiere: Von Alfred Brehm. Neubearbeitet von Franz Werner. Bibliographisches Institut (1912).
77. Weber, E. & Werner, Y.L. Vocalizations of two snake-lizards (Reptilia: Sauria: Pygopodidae). *Herpetologica*, pp.353-363 (1977).
78. Wever, W.G. Jr. Courtship and combat behaviour in *Gopherus berlandieri*. *Bull Fla State Mus* **15**:1–43 (1970).
79. Wojtusiak, R.J. & Majlert, Z. Bioacoustics of the voice of the tuatara, *Sphenodon punctatus punctatus*. *New Zealand Journal of Science*, **16(2),** pp.305-313 (1973).

80. Coleman, A. Sound Production in the Small-Mouthed Salamander (*Ambystoma texanum*) (Doctoral dissertation) (2016).
81. Davis, J. R., & Brattstrom, B. H. Sounds produced by the California newt, *Taricha torosa*. *Herpetologica,* **31,** 409-412 (1975).
82. Weber, E., & Schumacher, R. Der Aufbau der Abwehrrufe des Kammmolches (*Triturus cristatus*) und des Fadenmolches (*Triturus helveticus*) (Amphibia, Caudata, Salamandridae). *Salamandra*, **11,** 119-129 (1975).
83. Weber, E., & Schumacher, R. Verschiedenartige Abwehrrufe des Teichmolches (*Triturus v. vulgaris*) (Amphibia, Urodela). *Biologisches Zentralblatt,* **95,** 693-701 (1976).
84. Wyman, R. L., & Thrall, J. H. Sound production by the spotted salamander *Ambystoma maculatum*. *Herpetologica*, **28,** 210-212 (1972).
85. Neill, W. T. Remarks on salamander voices. *Copeia*, **1952(3),** 195-196 (1952).
86. M'Donnell, R. Observations on the habits and anatomy of the *Lepidosiren annectans*. *Natural History Review*, **7,** 93-112 (1860).
87. Lacroix, C., Davy, C.M. and Rollinson, N. Hatchling vocalizations and beneficial social interactions in subterranean nests of a widespread reptile. *Animal Behaviour*, **187**, pp.233-244 (2022).

As suggested during peer-review process, we included information about crocodilians to the list of species able to communicate using sounds (Supplementary material 1). The list of references related to this information is as follows:

1. Staniewicz, A. M. Acoustic communication of rare and threatened crocodilians and its use for population monitoring (Doctoral dissertation, University of Bristol) (2020).
2. Staniewicz, A., Foggett, S., McCabe, G., & Holderied, M. Courtship and underwater communication in the Sunda gharial (*Tomistoma schlegelii*). *Bioacoustics*, **31(4),** 435-449 (2022).
3. Dinets, V. Long-distance signaling in Crocodylia. *Copeia*, **2013(3),** 517-526 (2013).
4. Giddings, L. A. Behavioural Response to Juvenile Distress Calls as a Measure of Extended Care in Crocodilians (Doctoral dissertation, University of Bristol) (2020).

5. Compton, A. W. Courtship and nesting behavior of the freshwater crocodile, *Crocodylus johnstoni*, under controlled conditions. *Wildlife Research*, **8(2),** 443-450 (1981).
6. Webb G. & Manolis S. Crocodiles of Australia. Reed NewHolland, Sydney (1989).
7. Dinets, V. The Role of Habitat in Crocodilian Communication. (PhD). University of Miami, Miami, Florida (2011).
8. Garrick, L. D., & Lang, J. W. Social signals and behavior of adult alligators and crocodiles. *American Zoologist*, **17,** 225-239 (1977).
9. Vergne, A. L., Avril, A., Martin, S., & Mathevon, N. Parent–offspring communication in the Nile crocodile *Crocodylus niloticus*: do newborns' calls show an individual signature? *Naturwissenschaften*, **94(1),** 49-54 (2007).
10. Marquis, O., Mathevon, N., Aubin, T., Gaucher, P., & Lemaire, J. Observations on breeding site, bioacoustics and biometry of hatchlings of *Paleosuchus trigonatus* (Schneider, 1801) from French Guiana (Crocodylia: Alligatoridae). *Herpetology Notes*, **13,** 513-516 (2020).
11. Reber, S. A., Nishimura, T., Janisch, J., Robertson, M., & Fitch, W. T. A Chinese alligator in heliox: formant frequencies in a crocodilian. *Journal of Experimental Biology*, **218,** 2442-2447 (2015).
12. Todd, N. P. M. Estimated source intensity and active space of the American alligator (*Alligator Mississippiensis*) vocal display. *The Journal of the Acoustical Society of America*, **122(5),** 2906-2915 (2007).
13. Vergne, A. L., Pritz, M. B., & Mathevon, N.. Acoustic communication in crocodilians: from behaviour to brain. *Biological Reviews*, **84(3),** 391-411 (2009).
