# Supplementary material for: Common evolutionary origin of acoustic communication in choanate vertebrates
Source: Nat Commun. 2022 Oct 25;13:6089. doi: 10.1038/s41467-022-33741-8 (PMC9596459; doi:10.1038/s41467-022-33741-8)
Supplement: Supplementary file 3 — Description of Additional Supplementary Files [file 41467_2022_33741_MOESM3_ESM.pdf]

Description of Additional Supplementary Files

File Name: Supplementary Data 1
Description: List of choanate vertebrates often considered non-vocal, capable of producing sounds. Species belonging to Squamata were not altered in our phylogenetic analysis. PW refers to species recorded in the present work. Only species that were not accounted as vocal by Chen & Wiens (2020) were included in this list. Species that appear in bold were used in analysis one (choanate vertebrates' phylogeny).

File Name: Supplementary Data 2
Description: Interactive sound file with the acoustic repertoire of species recorded in the present work. (shorturl.at/cwMU2)

File Name: Supplementary Data 3
Description: Repertoire descriptions of the species recorded in the present work. Sound type numeration follows Sup. Mat. 2. Min frequency possible is 0.05 KHz due to equipment limitations. Values are approximated. The column 'enclosure' refers to information about the size and material of enclosure used during recordings.

File Name: Supplementary Data 4
Description: Input dataset for choanate vertebrates' ancestral state reconstruction analysis. State 1= presence and 0=absence of data/acoustic communication. Species in bold were previously considered to lack with acoustic communication by Chen and Wiens, 2020.

File Name: Supplementary Data 5
Description: Input dataset for turtle ancestral state reconstruction analysis. State 1= presence and 0=absence of data/acoustic communication. Presence of character was assigned to every genus with at least one species know to produce sounds.

File Name: Supplementary Code 1
Description: R code and input files.
