# Supplementary material for: Common evolutionary origin of acoustic communication in choanate vertebrates
Source: Nat Commun. 2022 Oct 25;13:6089. doi: 10.1038/s41467-022-33741-8 (PMC9596459; doi:10.1038/s41467-022-33741-8)
Supplement: Supplementary file 5 — Supplementary Data 2 [file 41467_2022_33741_MOESM5_ESM.zip › Supplementary Data 2.pdf]

Common evolutionary origin of acoustic communication in vertebrates

Supplementary material 2

Jorgewich-Cohen et al.

For your best experience, turn on presentation mode.

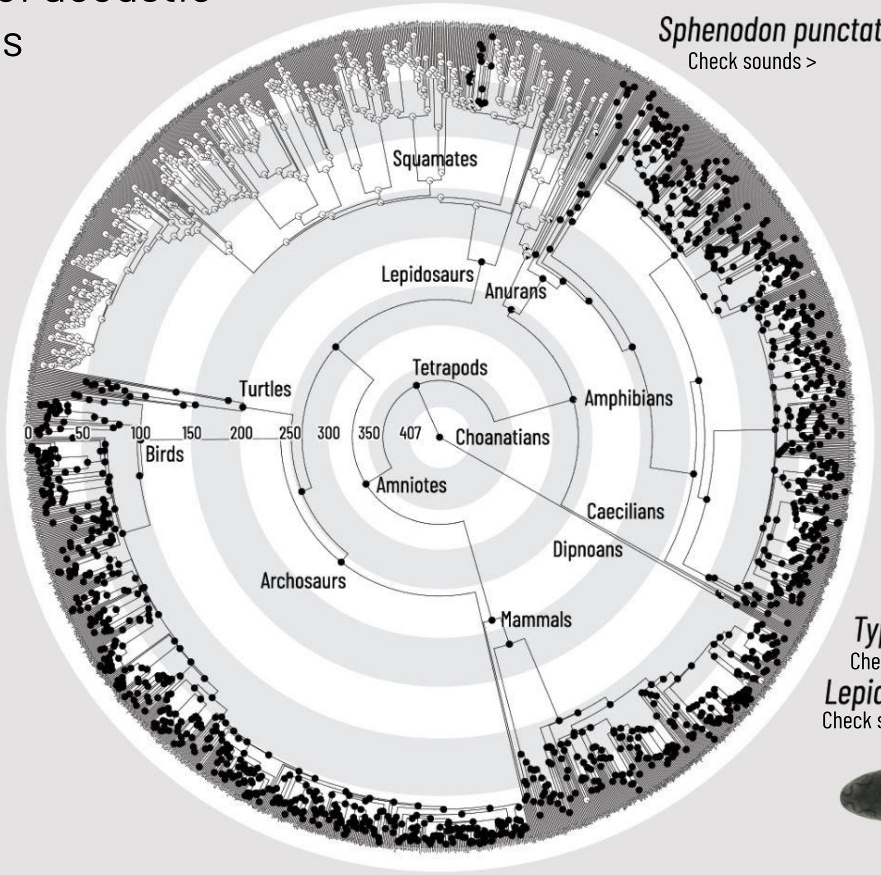

Sphenodon punctatus
Check sounds >

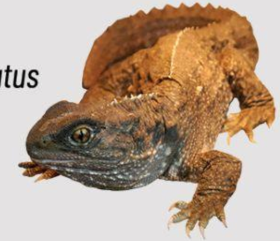

Turtles
Check sounds >

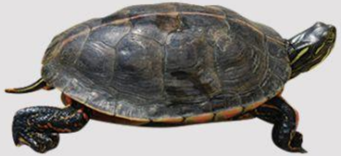

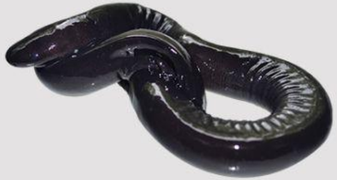

Typhlonectes compressicauda
Check sounds >

Lepidosiren paradoxa
Check sounds >

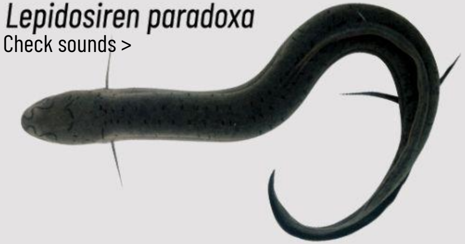

● Presence of acoustic communication
○ Absence of data

Lepidosiren paradoxa

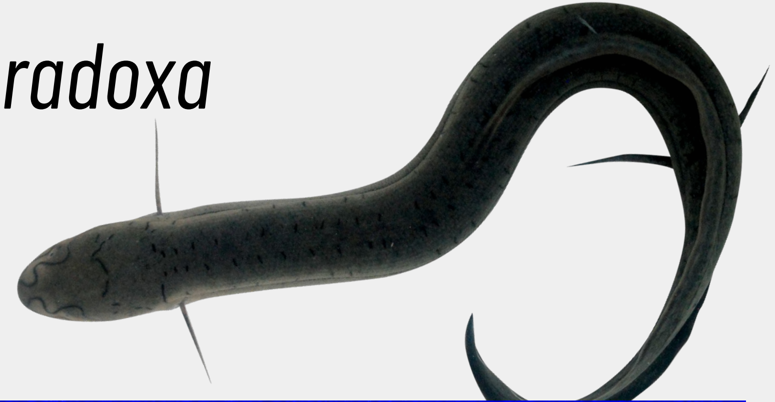

1

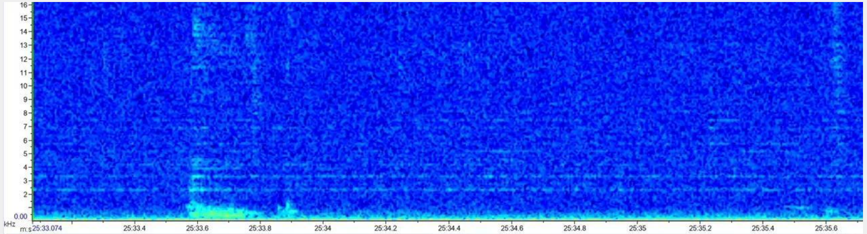

2

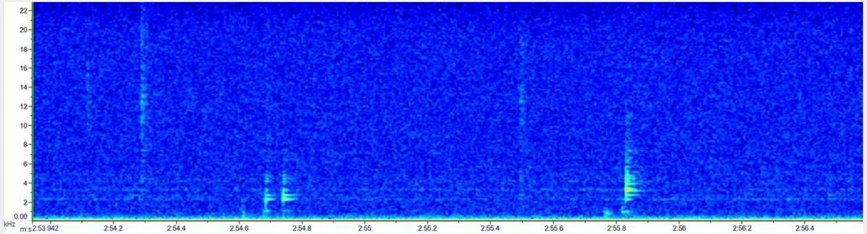

3

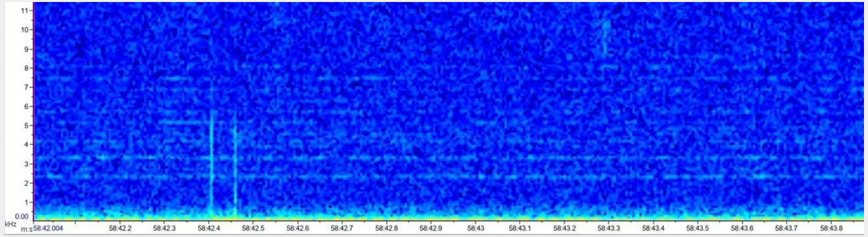

4

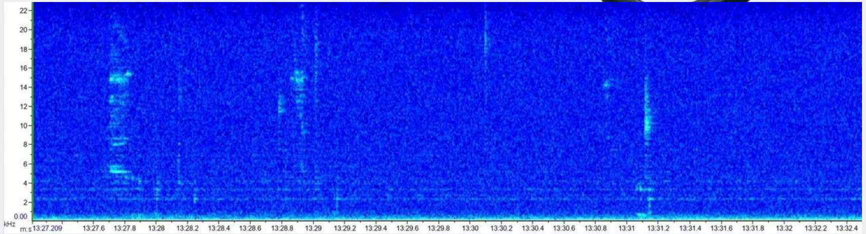

5

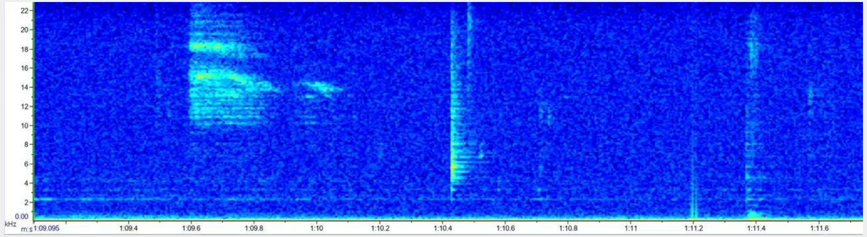

Typhlonectes compressicauda

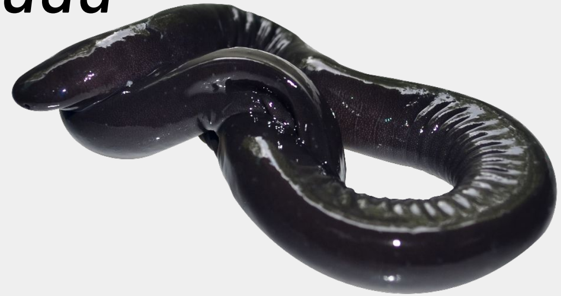

1

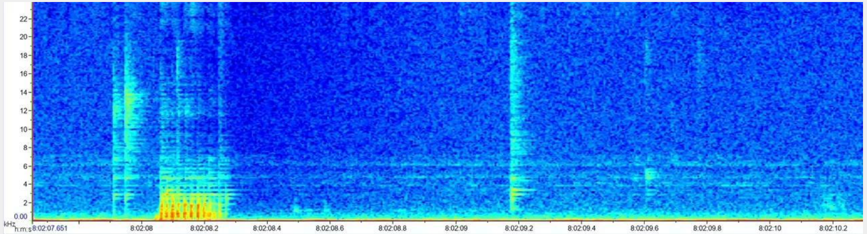

2

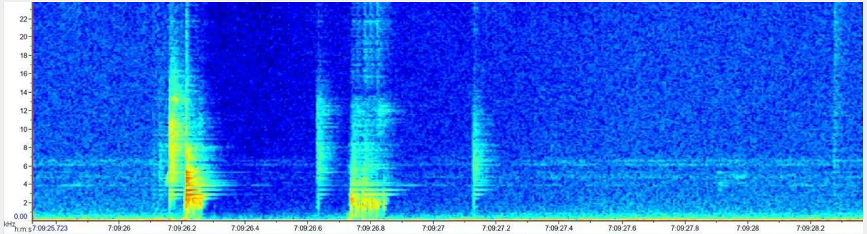

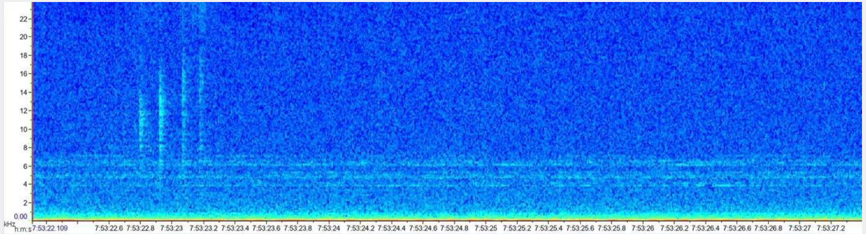

4

3

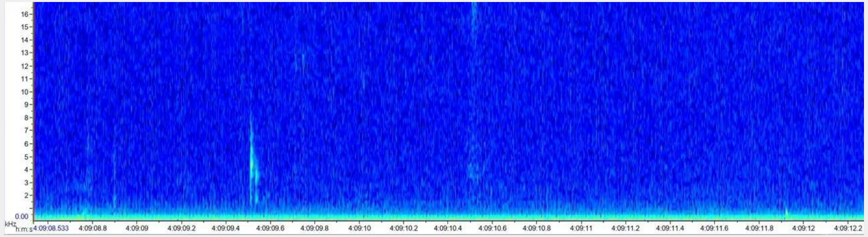

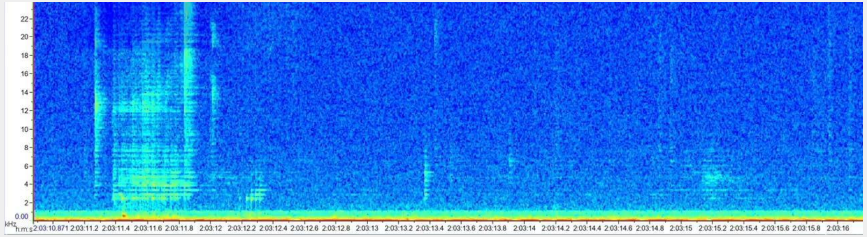

5

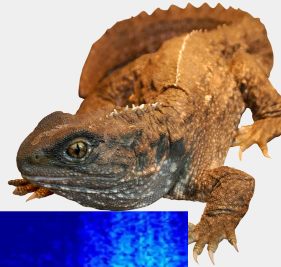

Sphenodon punctatus

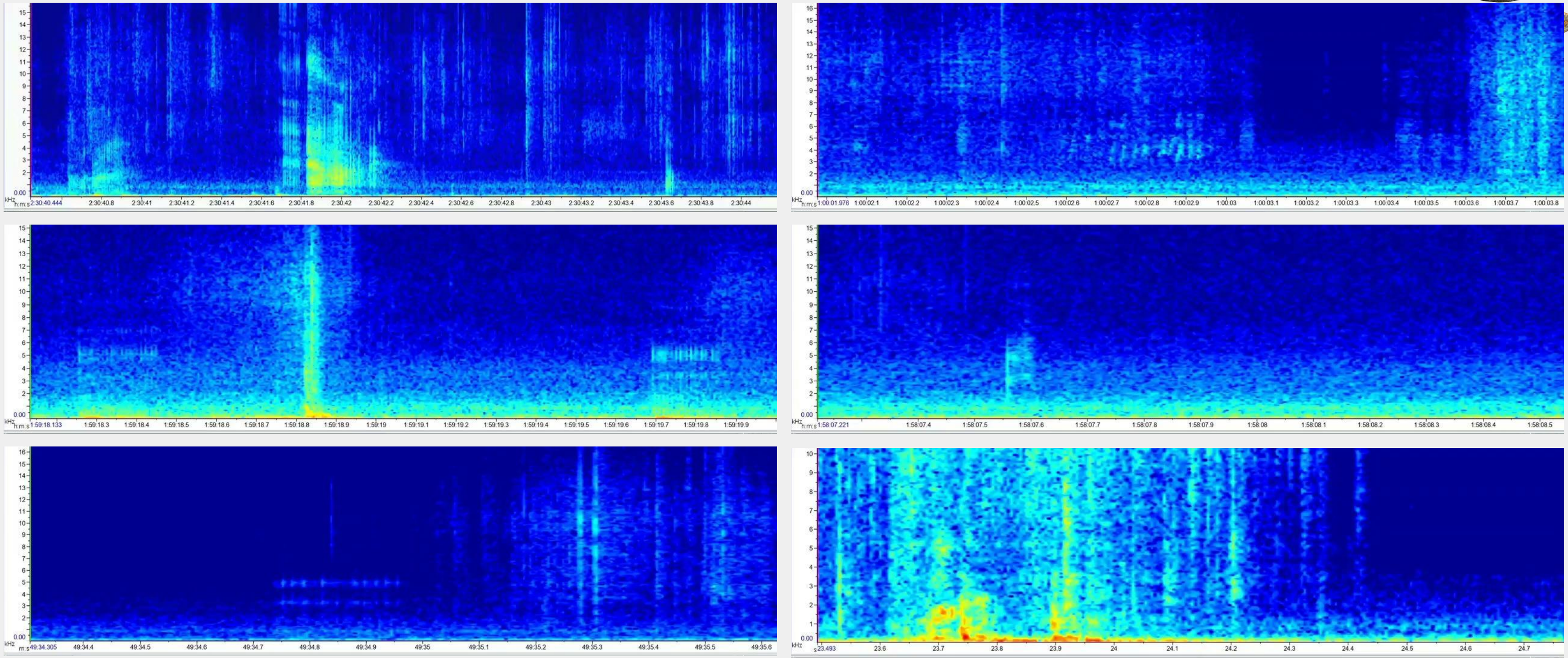

1 2 3 4 5 6

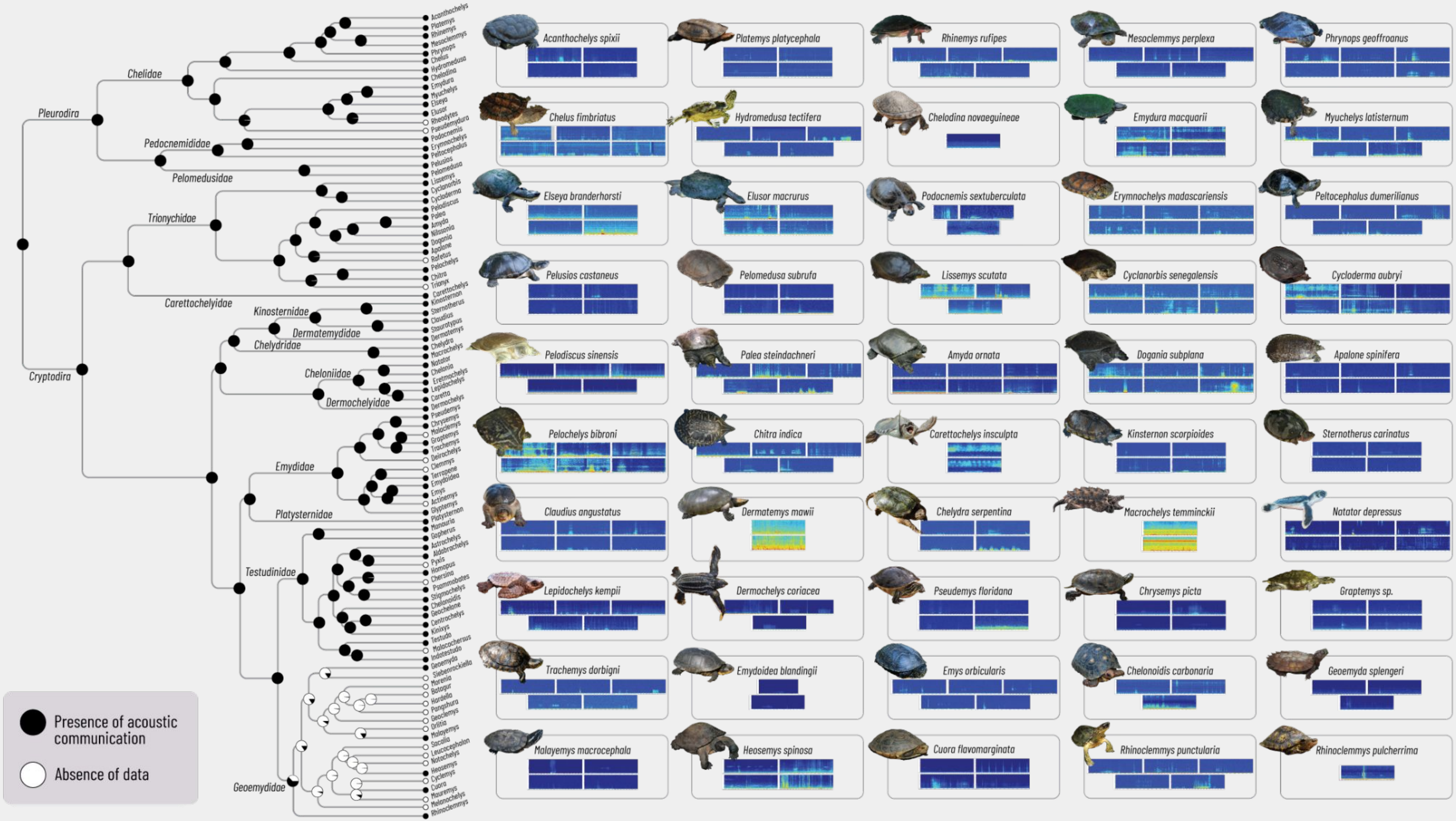
Pleurodira
Chelidae
Pedocnemididae
Pelomedusidae
Trionychidae
Carettochelyidae
Kinosternidae
Dermatemydidae
Chelydridae
Cheloniidae
Dermochelyidae
Cryptodira
Emydidae
Platysternidae
Testudinidae
Geoemydidae
Presence of acoustic communication
Absence of data
Acanthochelys spixii
Platemys platycephala
Rhinemys rufipes
Mesoclemmys perplexa
Phrynops geoffroanus
Chelus fimbriatus
Hydromedusa tectifera
Chelodina novaeguineae
Emydura macquarii
Myuchelys latisternum
Elseya branderhorsti
Elusor macrurus
Podocnemis sextuberculata
Erymnochelys madascariensis
Peltocephalus dumerilianus
Pelusios castaneus
Pelomedusa subrufa
Lissemys scutata
Cyclanorbis senegalensis
Cycloderma aubryi
Pelodiscus sinensis
Palea steindachneri
Amyda ornata
Dogania subplana
Apalone spinifera
Pelochelys bibroni
Chitra indica
Carettochelys insculpta
Kinsternon scorpioides
Sternotherus carinatus
Claudius angustatus
Dermatemys mawii
Chelydra serpentina
Macrochelys temminckii
Natator depressus
Lepidochelys kempii
Dermochelys coriacea
Pseudemys floridana
Chrysemys picta
Graptemys sp.
Trachemys dorbigni
Emydoidea blandingii
Emys orbicularis
Chelonoidis carbonaria
Geoemyda splengeri
Malayemys macrocephala
Heosemys spinosa
Cuora flavomarginata
Rhinoclemmys punctularia
Rhinoclemmys pulcherrima

Chelidae

Acanthochelys spixii

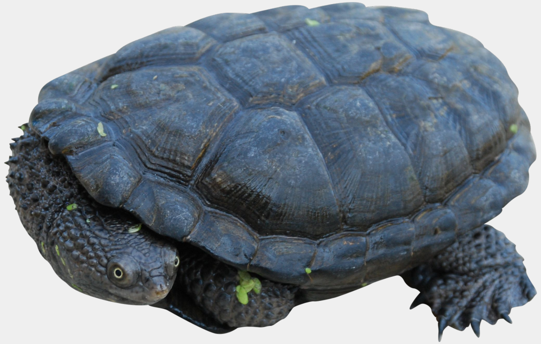

1
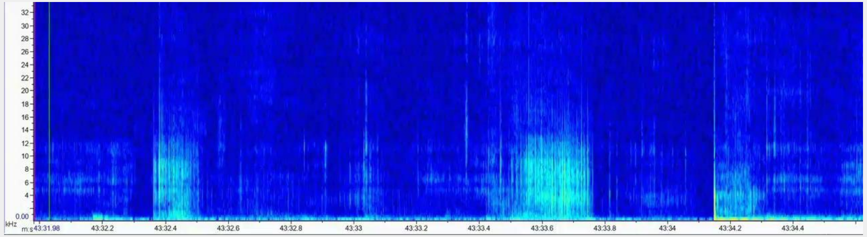

2
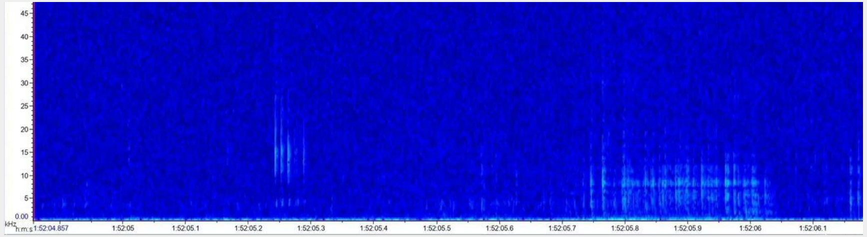

3
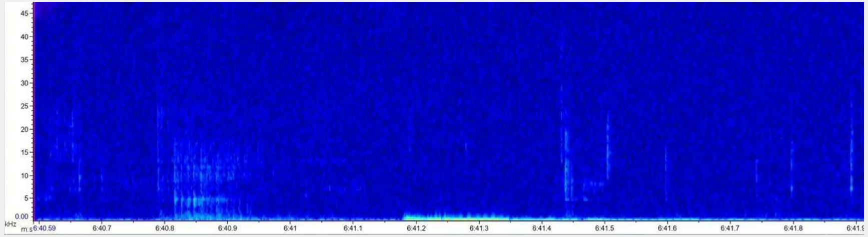

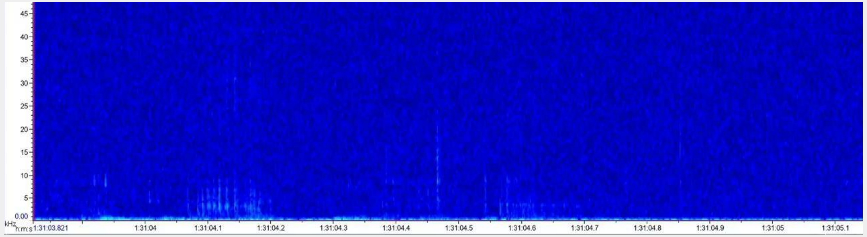
4

< Turtles tree

Next >

Chelidae

Platemys platycephala

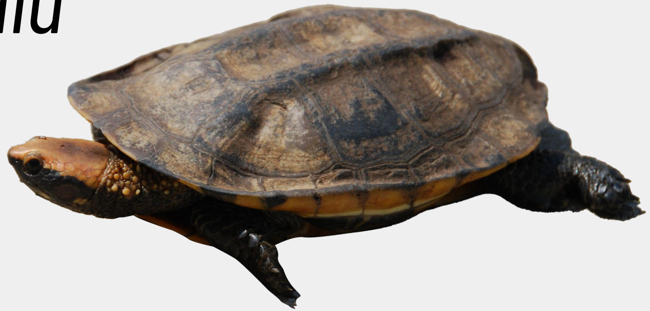

1
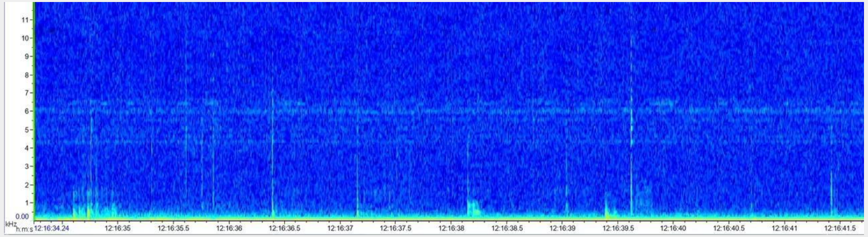

2
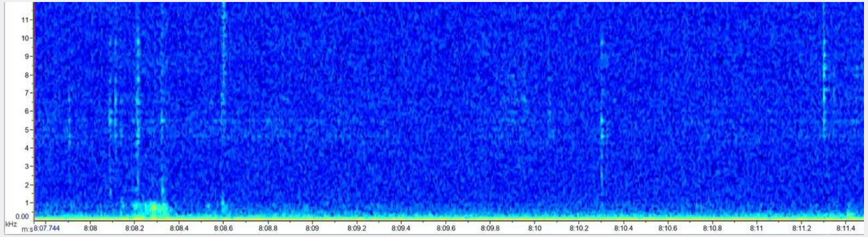

3
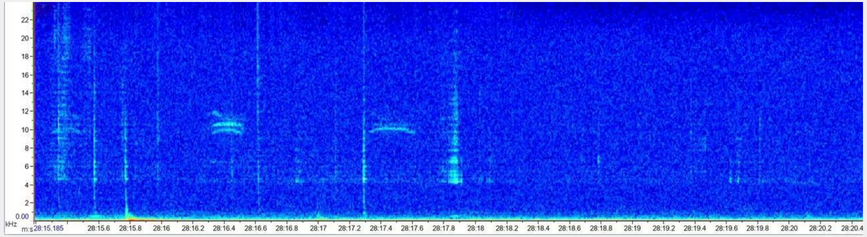

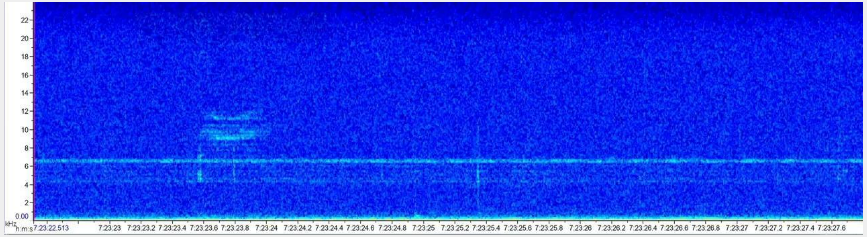
4

Chelidae

Rhinemys rufipes

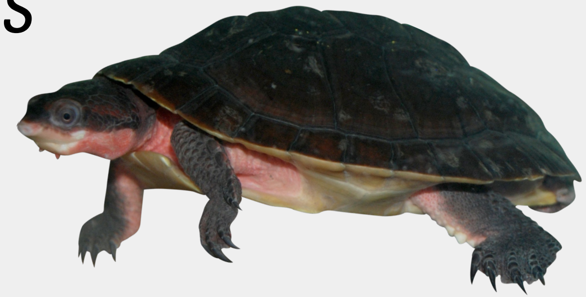

1
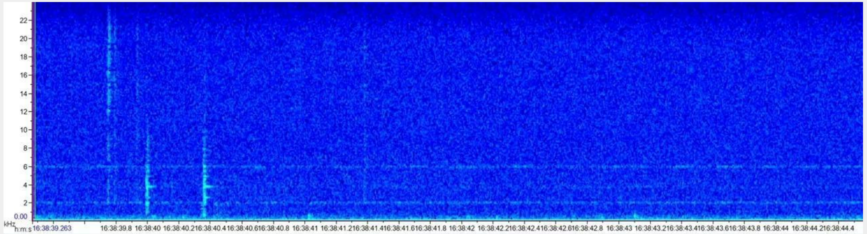

2
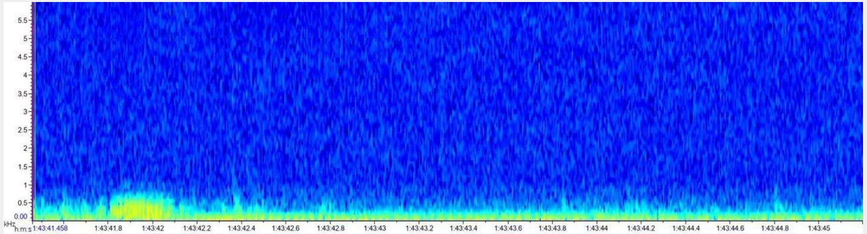

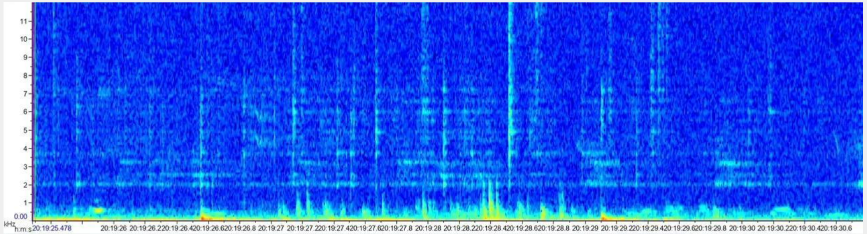
4

3
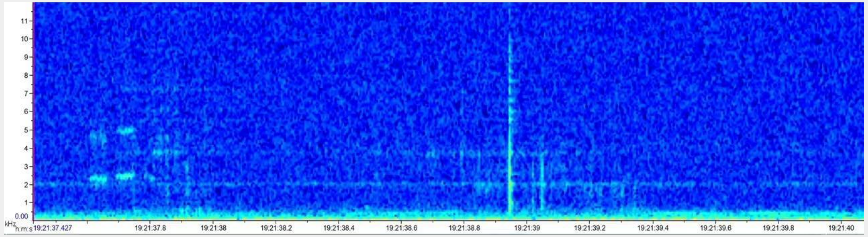

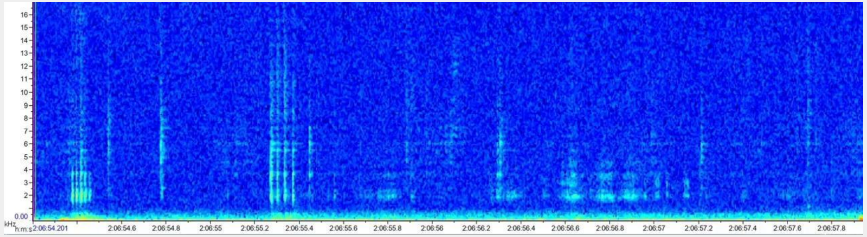
5

< Turtles tree

Next >

Chelidae

Mesoclemmys perplexa

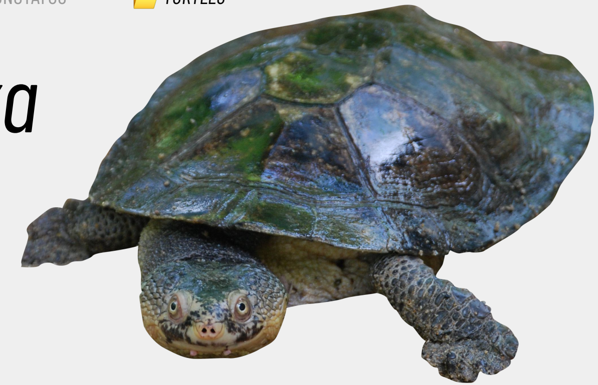

1

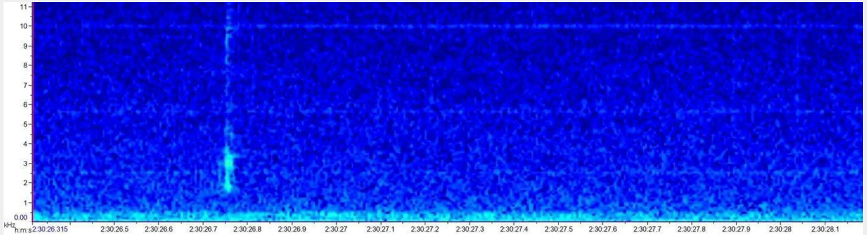

2

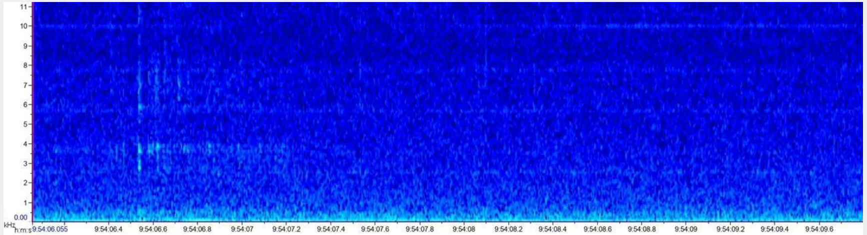

4

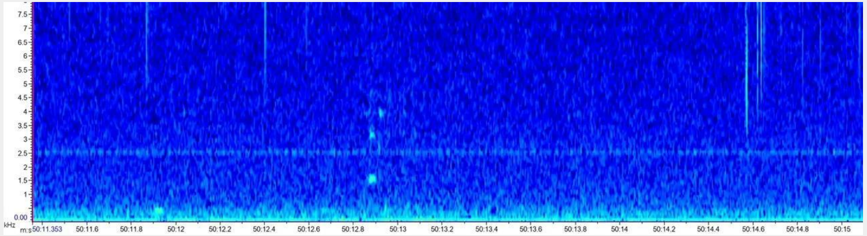

3

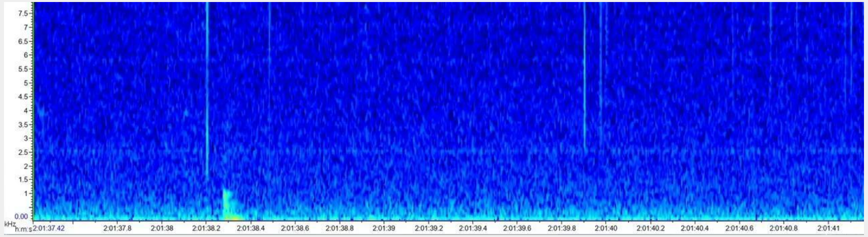

5

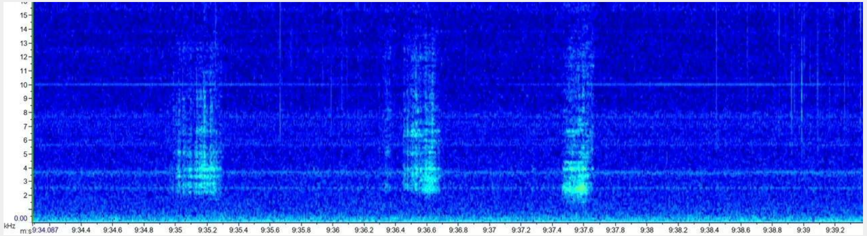

< Turtles tree Next >

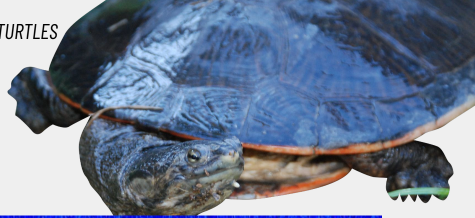

Chelidae

Phrynops geoffroanus

1

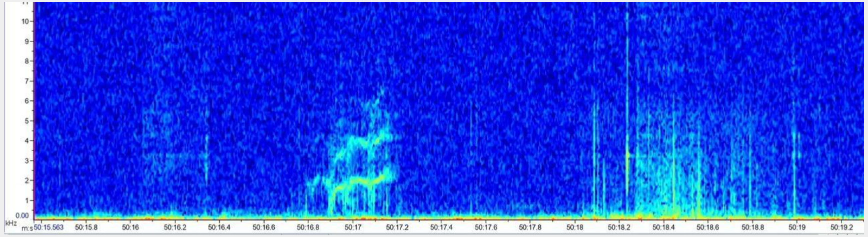

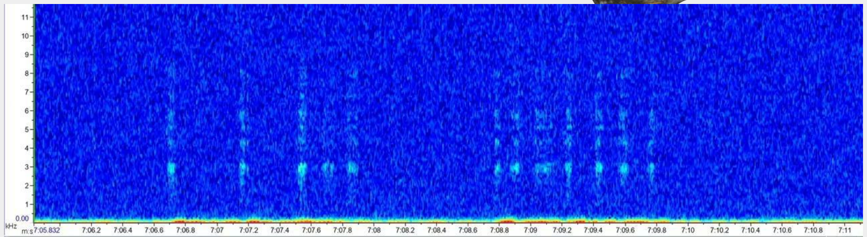

4

2

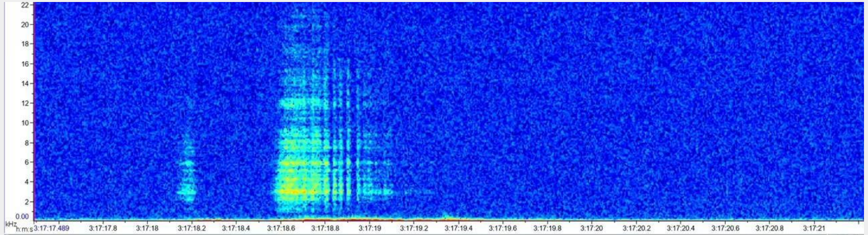

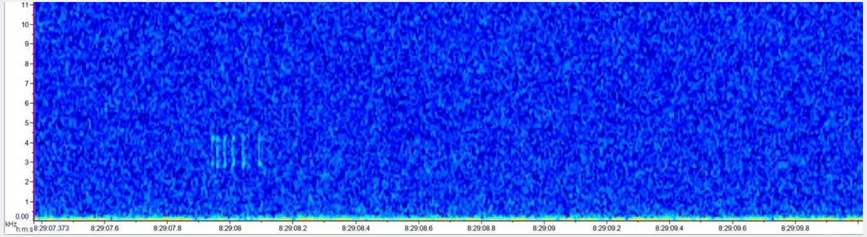

5

3

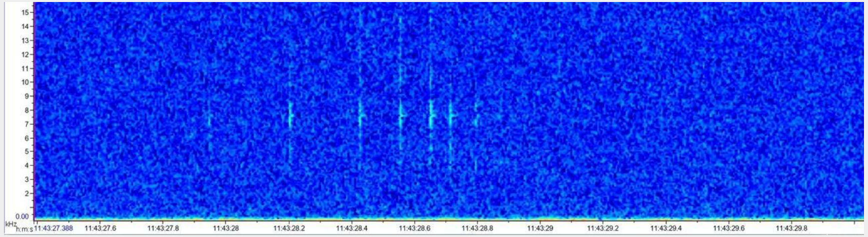

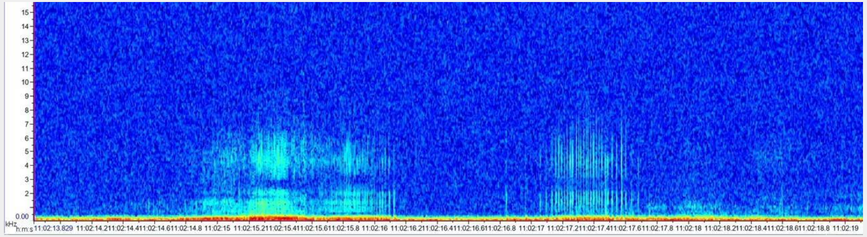

6

< Turtles tree

Next >

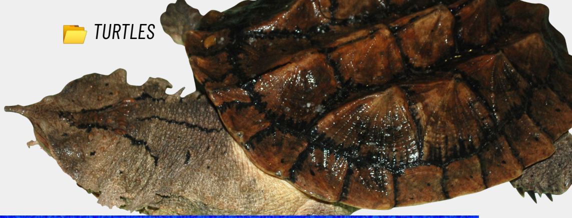

Chelidae

Chelus fimbriatus

1

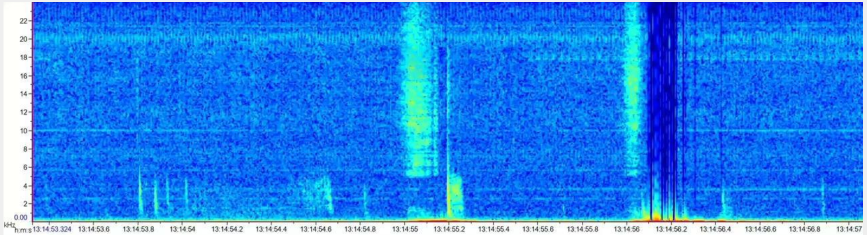

4

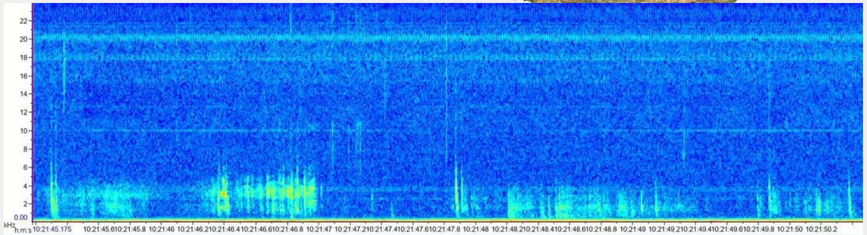

2

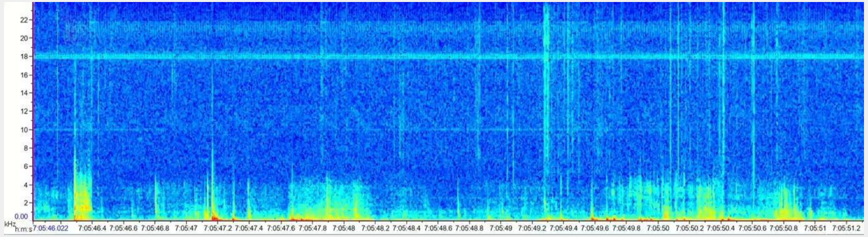

5

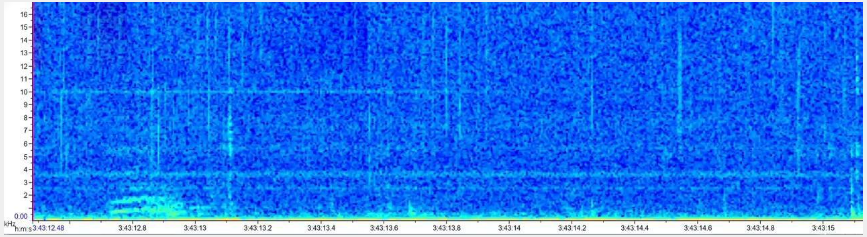

3

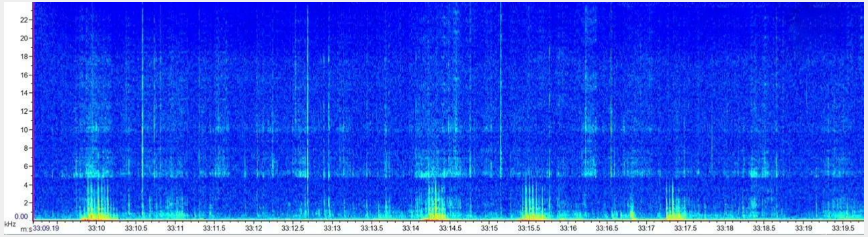

6

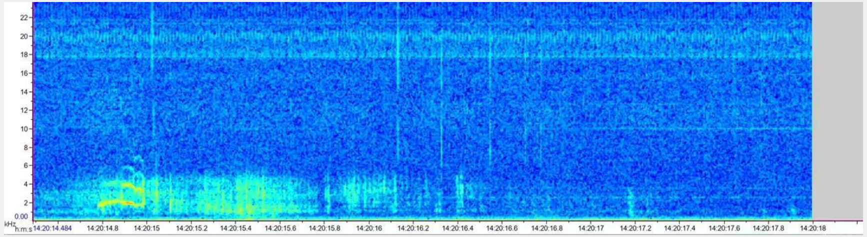

< Turtles tree

Next >

Chelidae

Hydromedusa tectifera

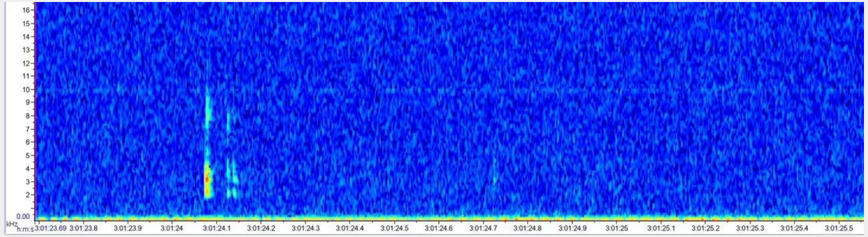

1

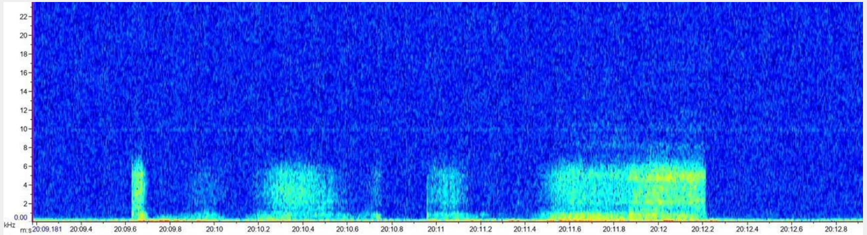

2

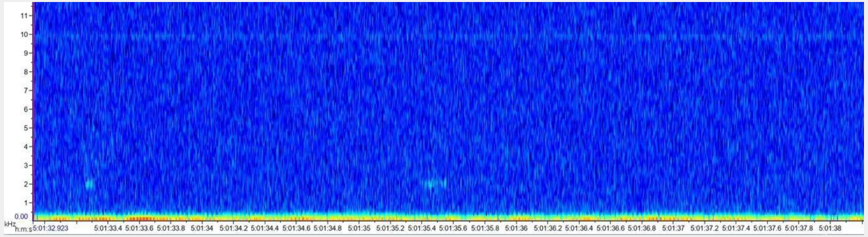

3

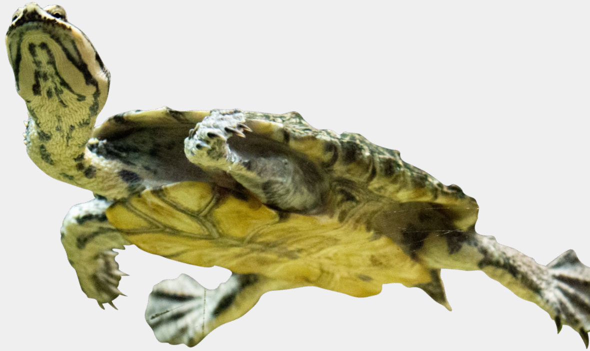

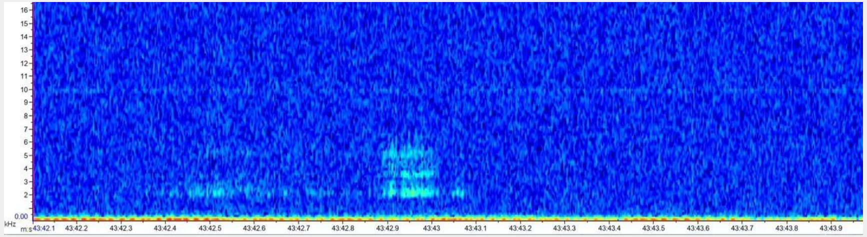

4

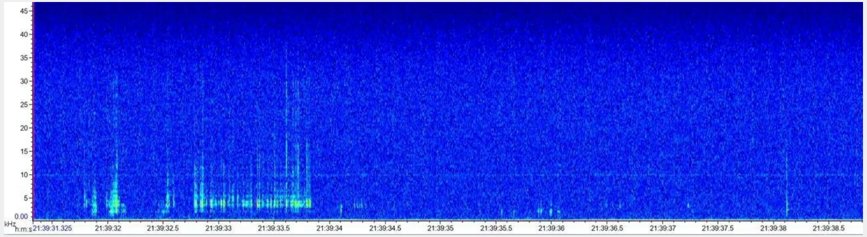

5

< Turtles tree

Next >

Chelidae

Chelodina novaeguineae

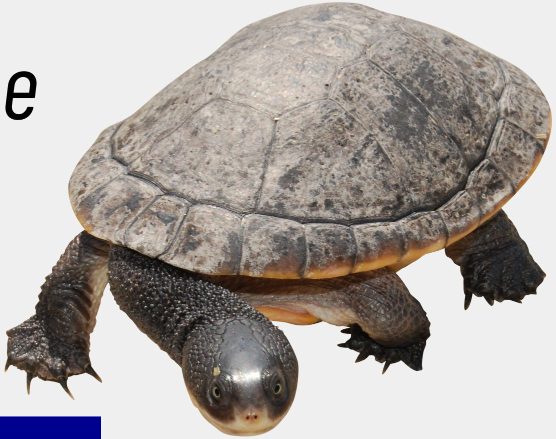

1

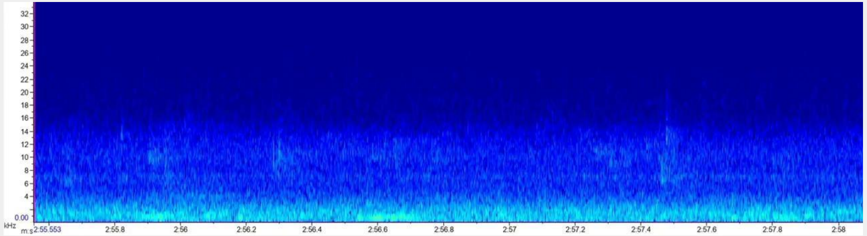

Chelidae

Emydura macquarii

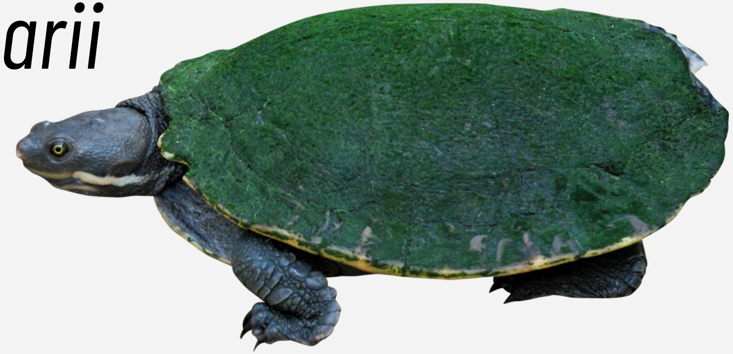

1

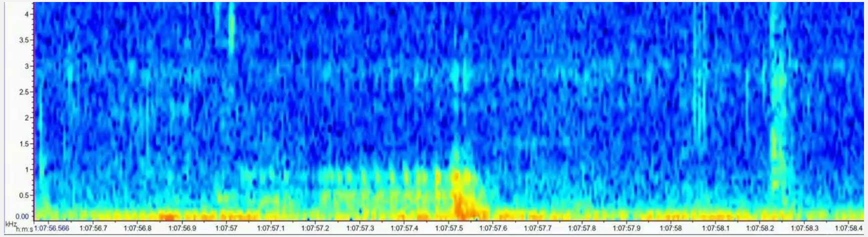

2

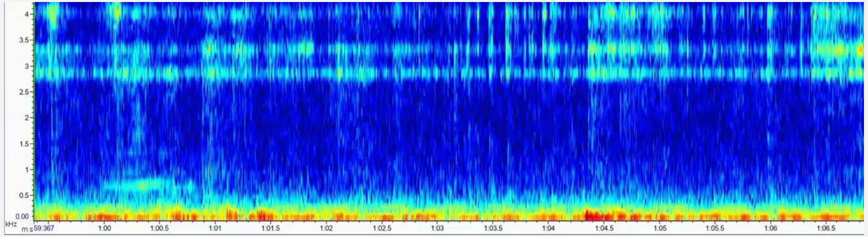

3

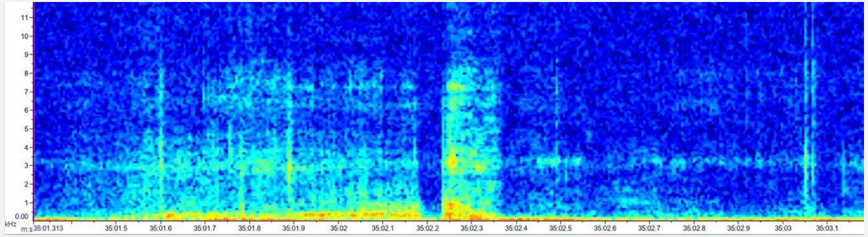

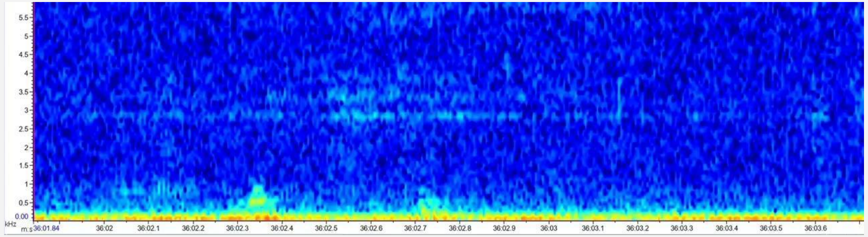

4

< *Turtles tree*

Next >

Chelidae

Myuchelys latisternum

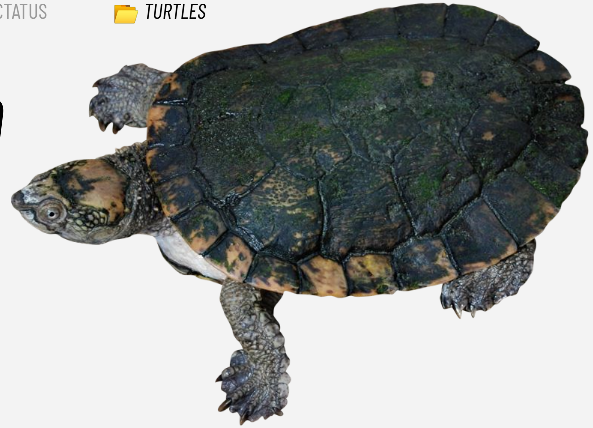

1

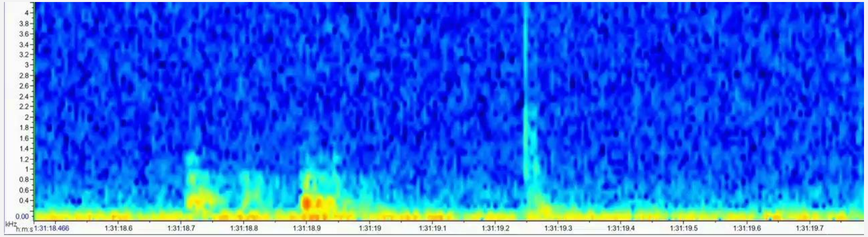

2

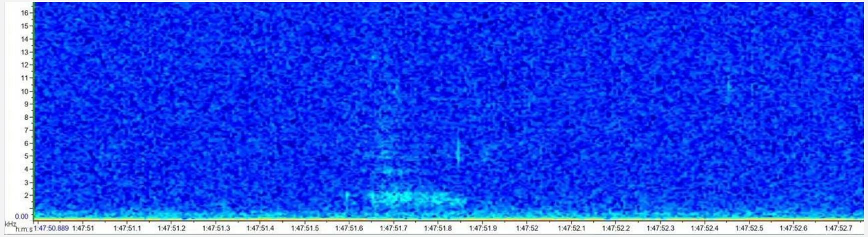

4

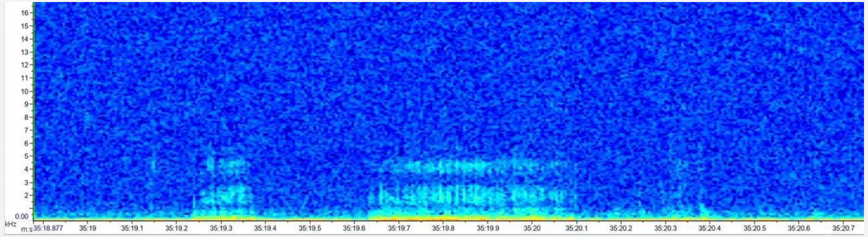

3

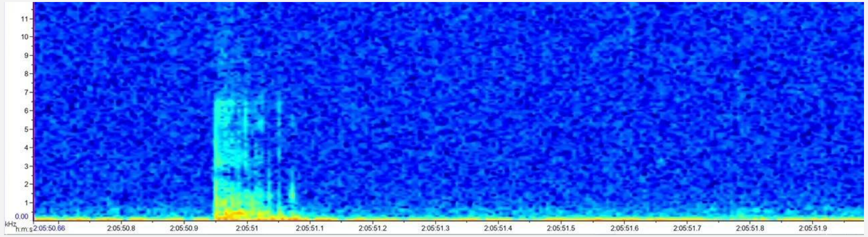

5

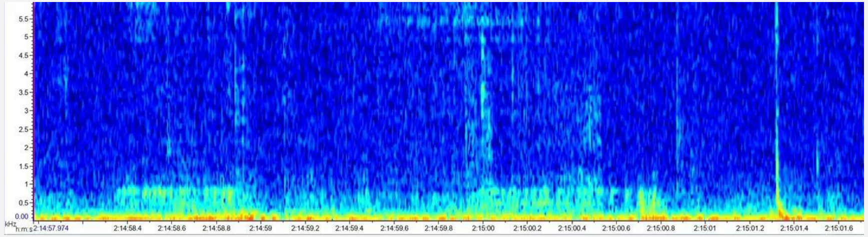

< Turtles tree

Next >

Chelidae

Elseya branderhorsti

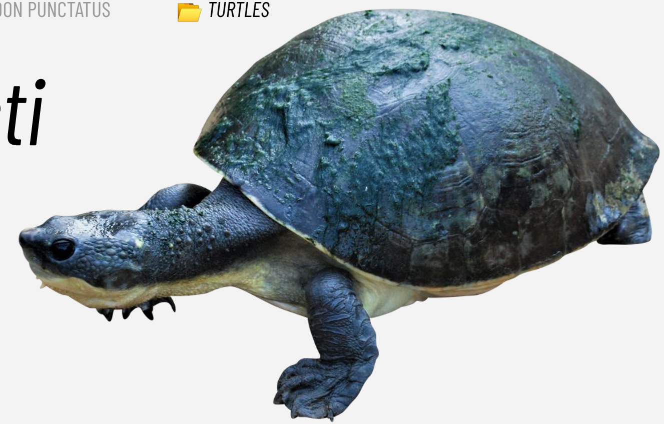

1

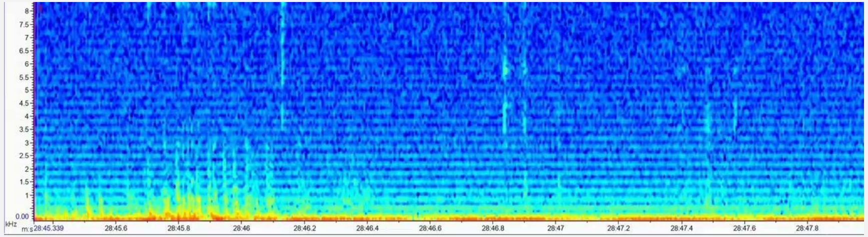

2

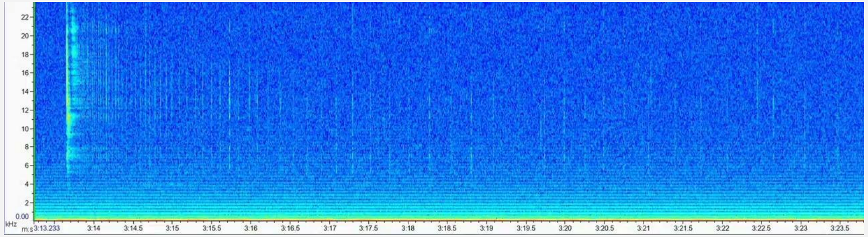

3

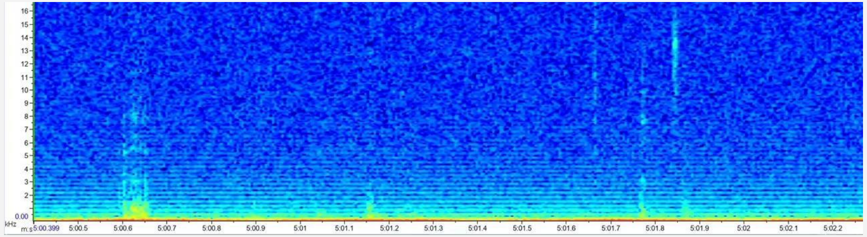

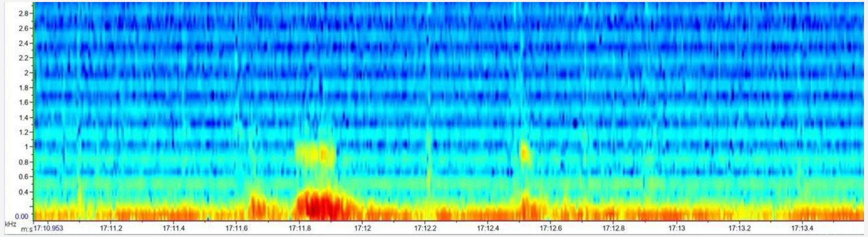

4

Chelidae

Elusor macrurus

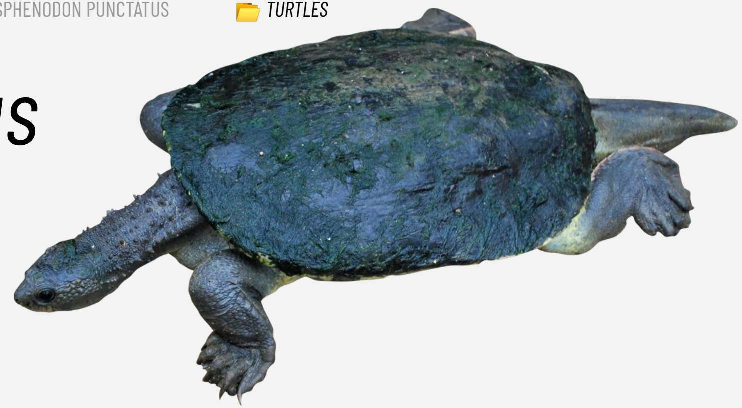

1

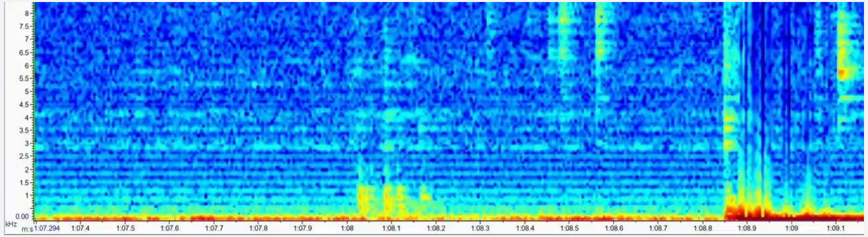

2

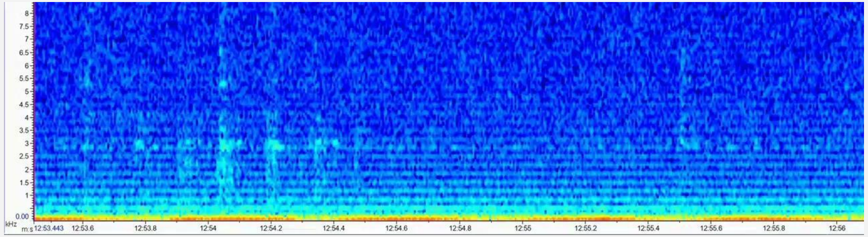

3

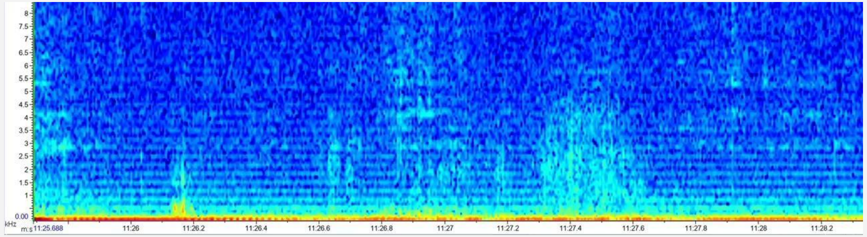

4

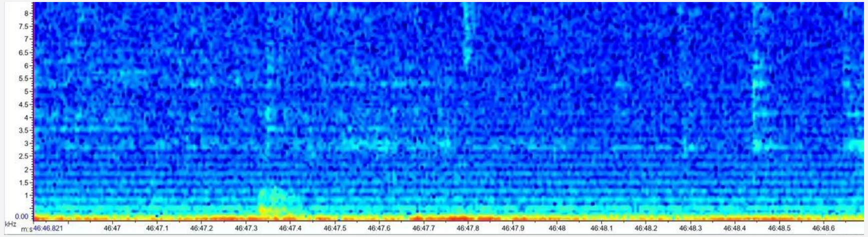

< Turtles tree

Next >

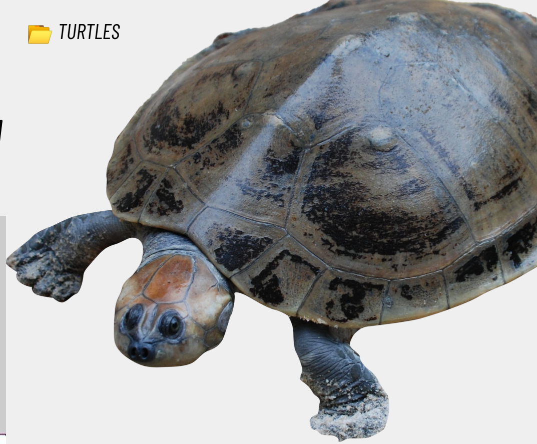

Podocnemididae

Podocnemis sextuberculata

1

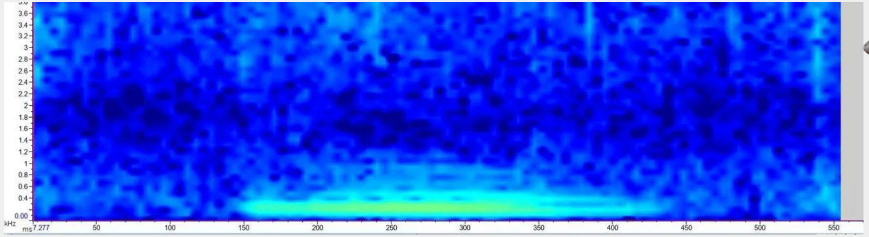

2

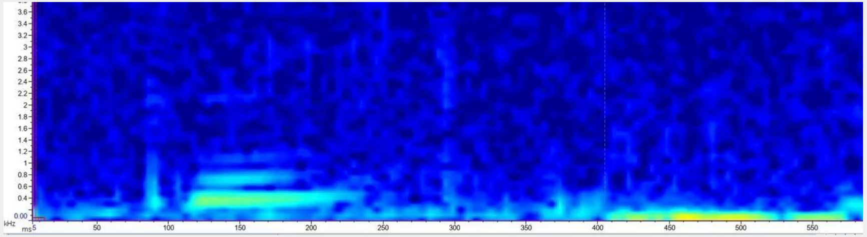

3

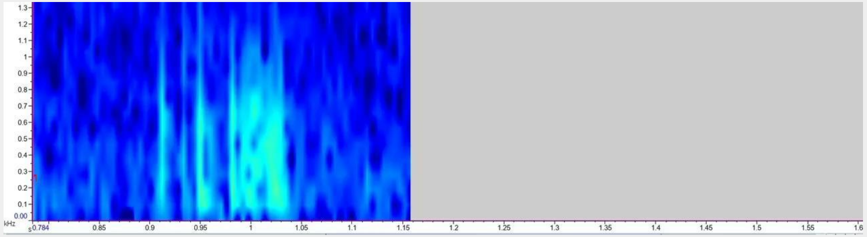

Podocnemididae

Erymnochelys madagascariensis

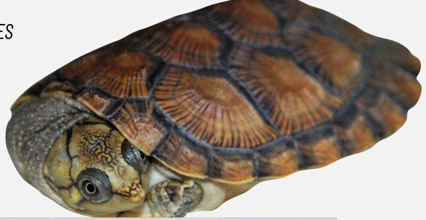

1

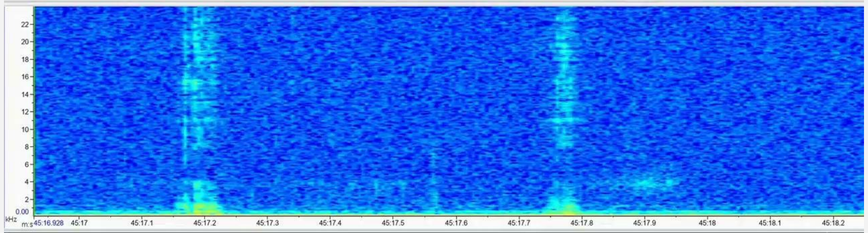

4

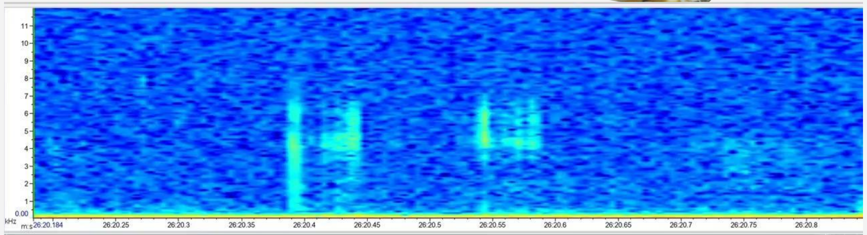

2

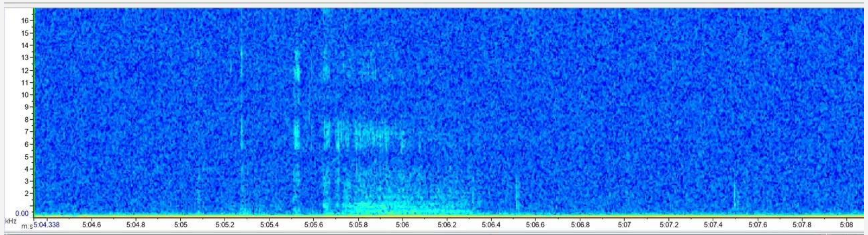

5

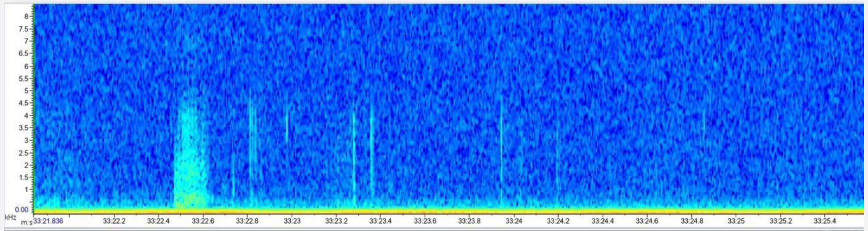

3

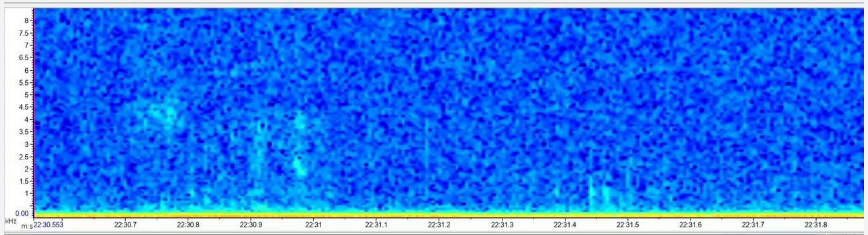

6

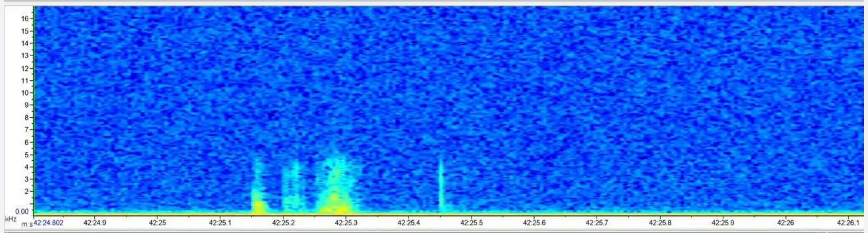

< Turtles tree

Next >

Podocnemididae

Peltocephalus dumerilianus

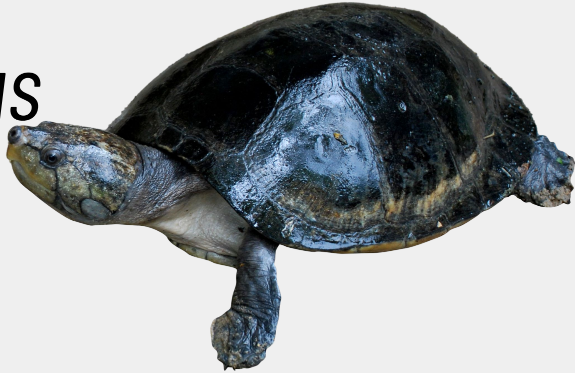

1

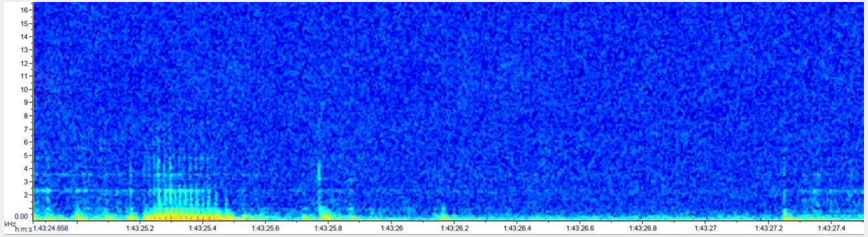

2

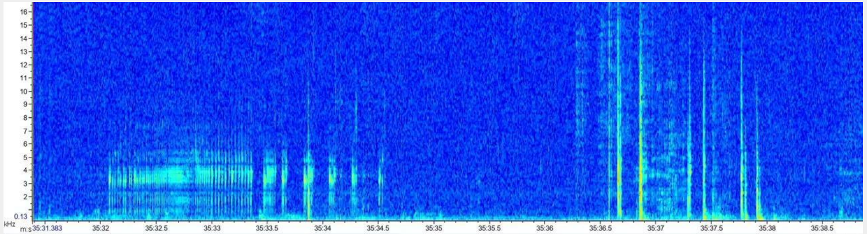

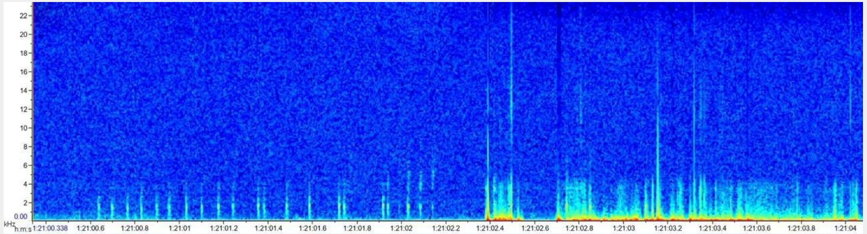

4

3

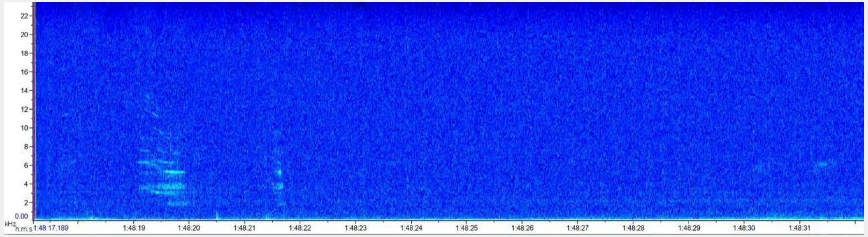

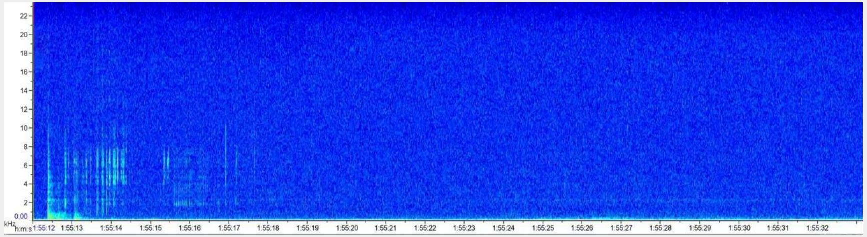

5

< Turtles tree

Next >

Pelomedusidae

Pelusios castaneus

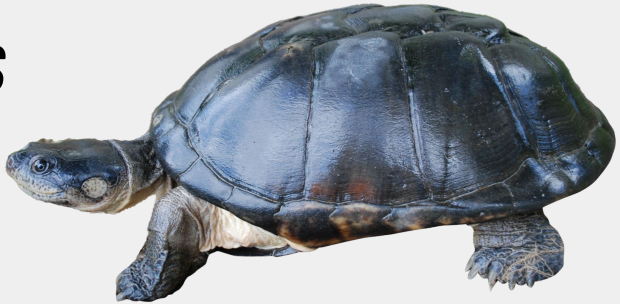

1

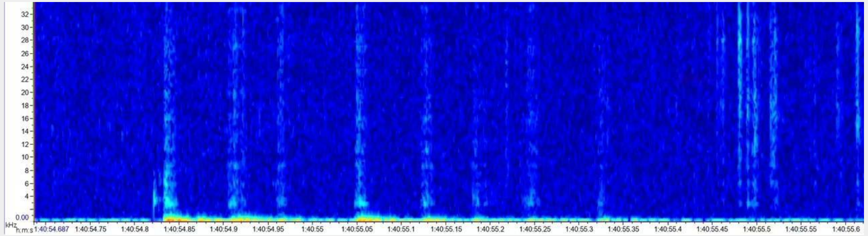

2

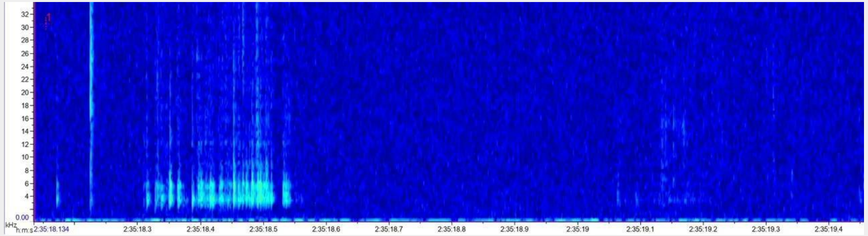

3

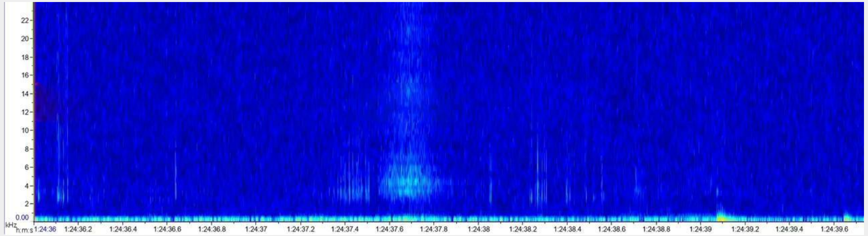

4

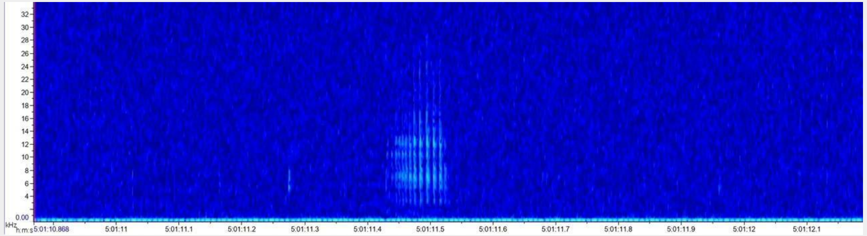

< Turtles tree

Next >

Pelomedusidae

Pelomedusa subrufa

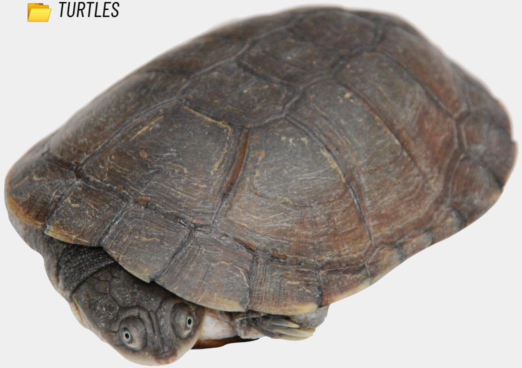

1 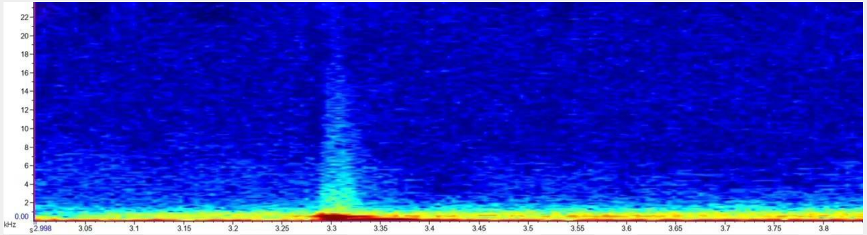

2 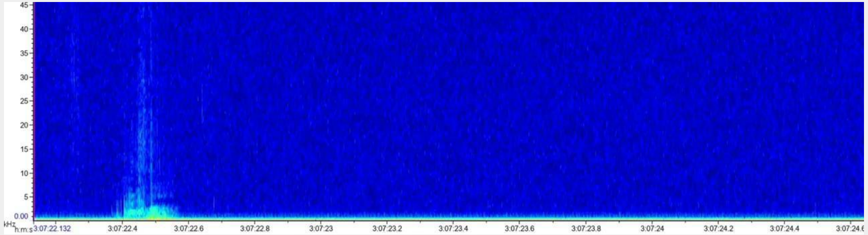

3 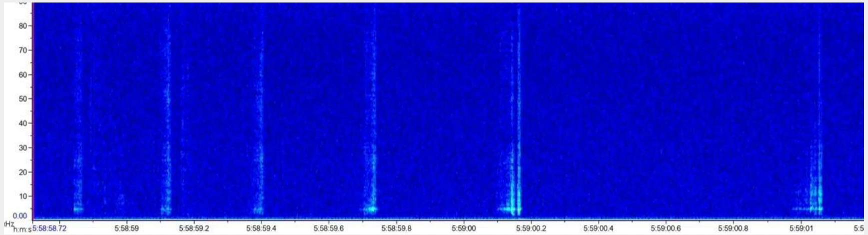

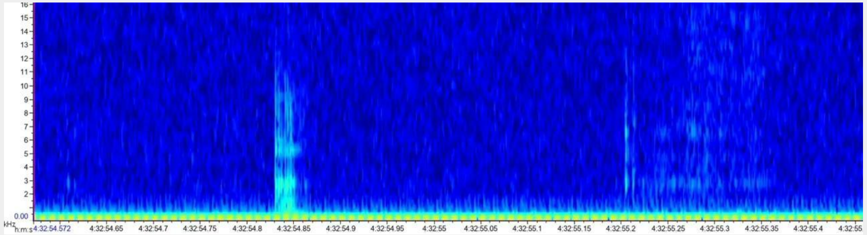 4

Trionychidae

Lissemys scutata

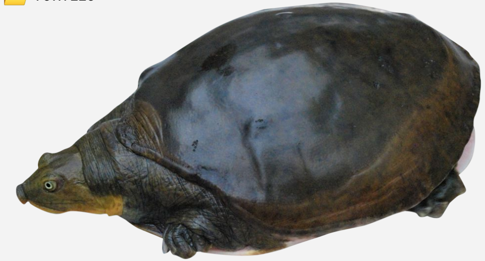

1

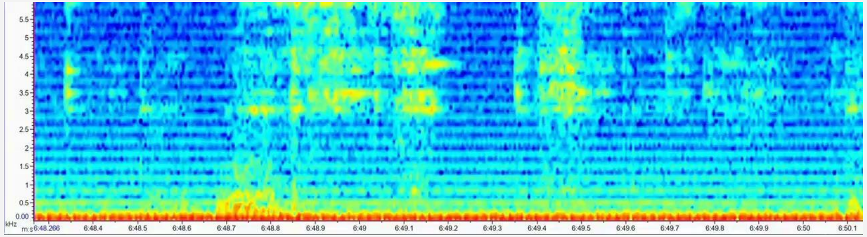

2

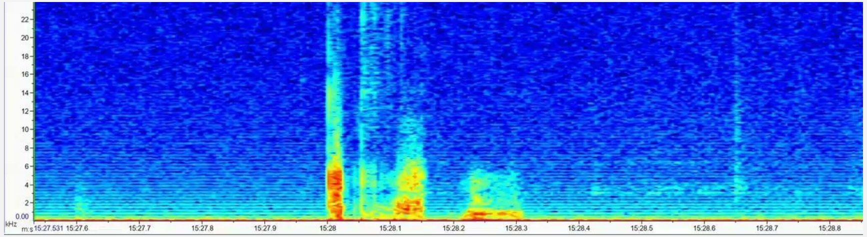

3

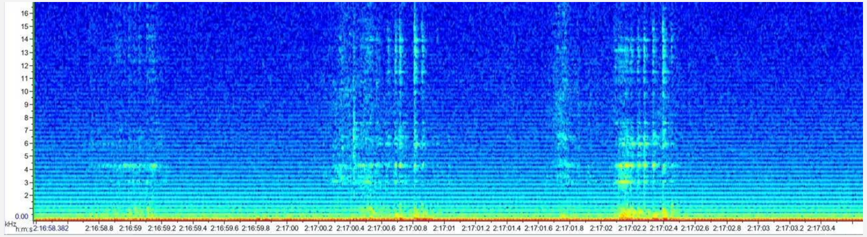

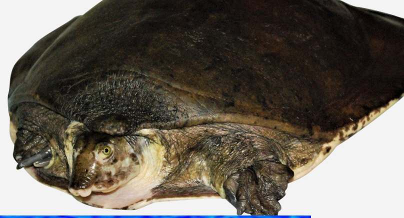

Trionychidae

Cyclanorbis senegalensis

1

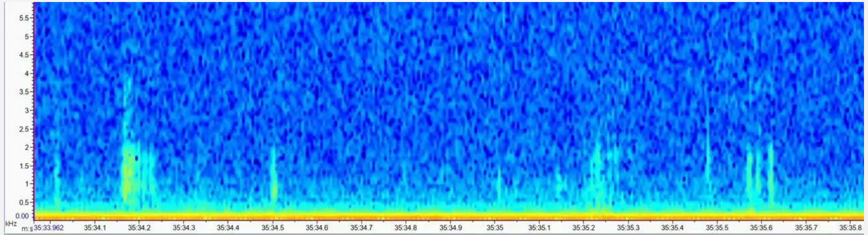

4

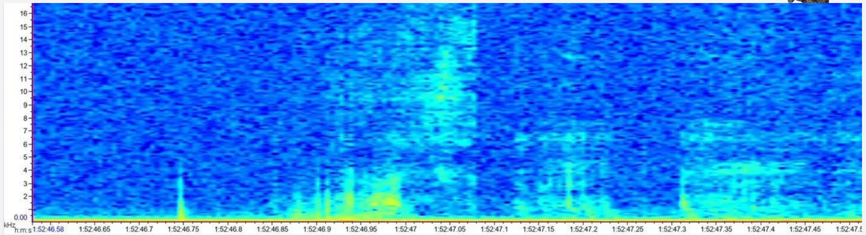

2

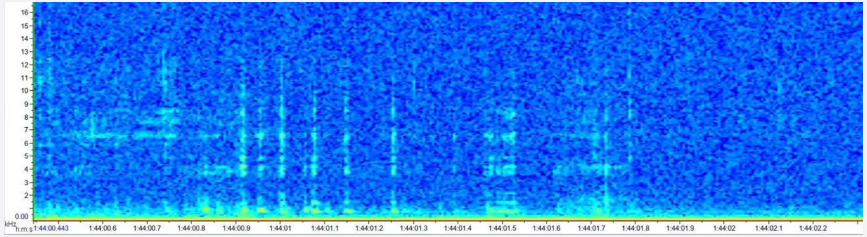

5

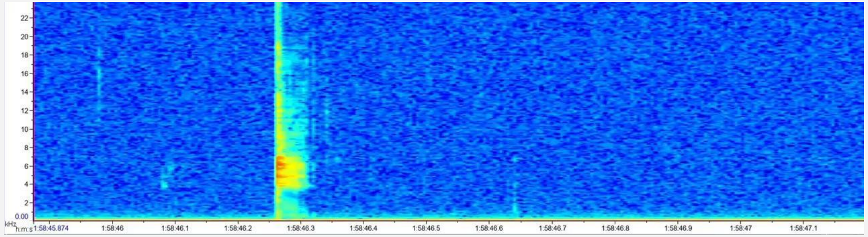

3

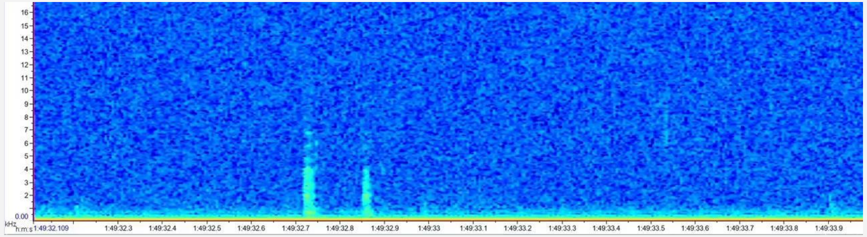

6

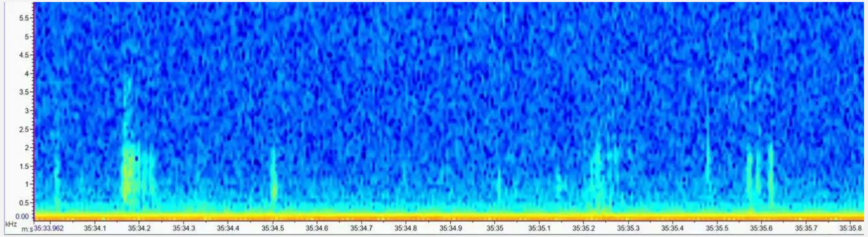

Trionychidae

Cycloderma aubryi

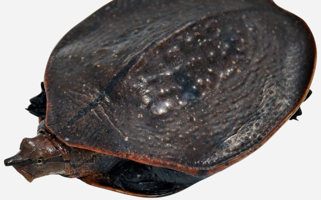

1

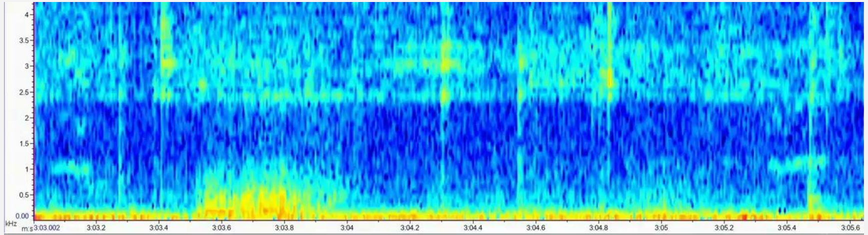

2

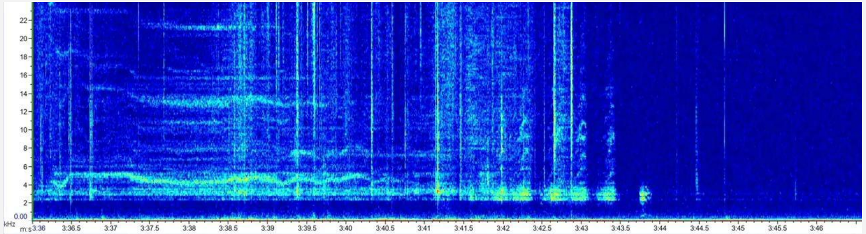

3

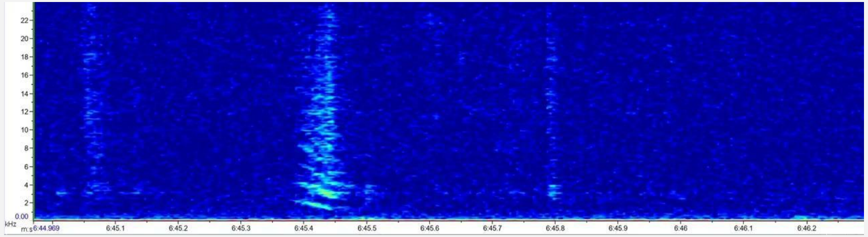

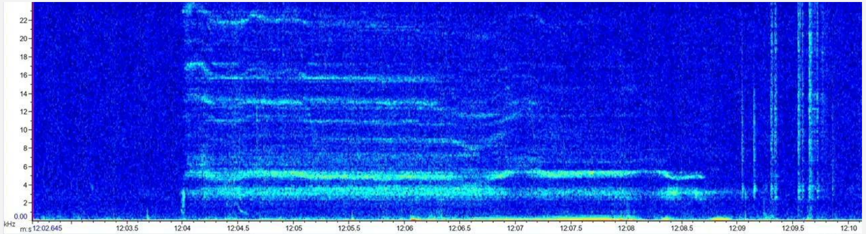

4

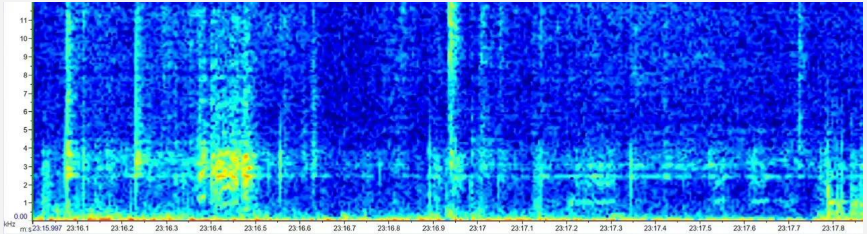

5

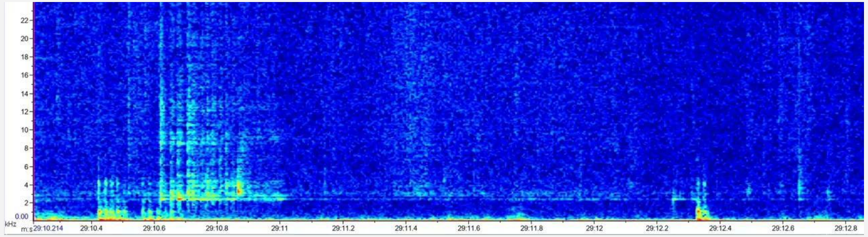

6

Trionychidae

Pelodiscus sinensis

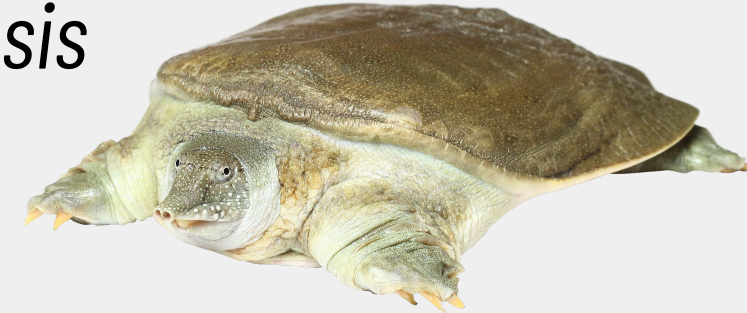

1

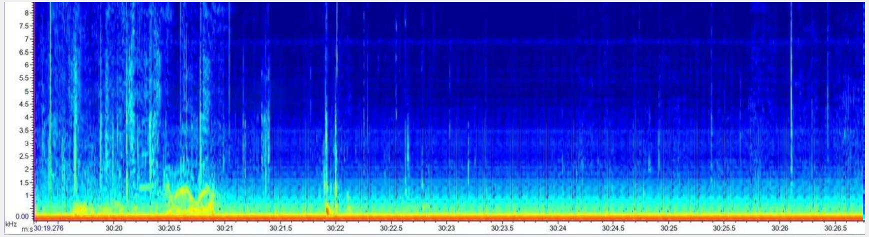

2

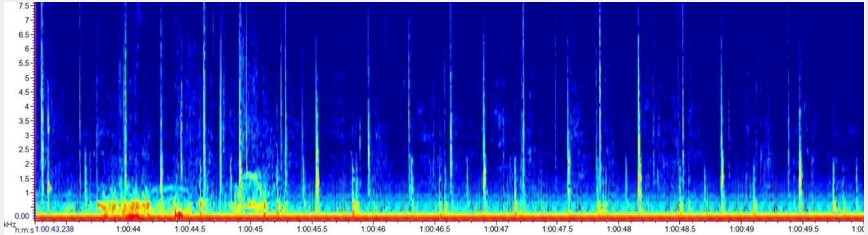

4

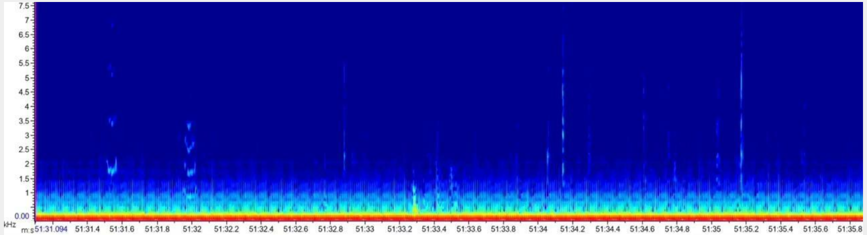

3

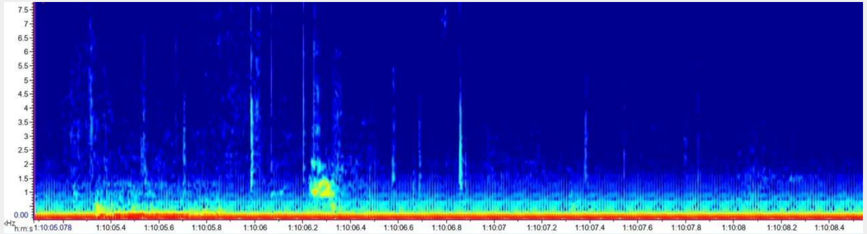

5

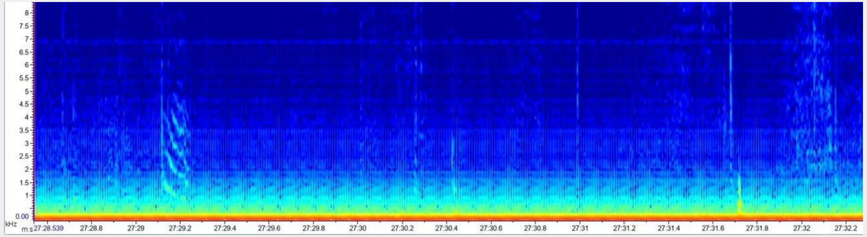

< Turtles tree

Next >

Trionychidae

Palea steindachneri

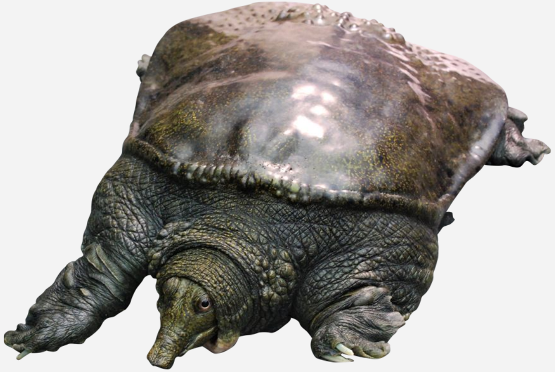

1

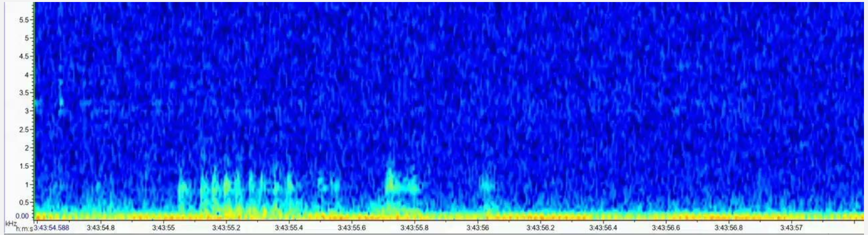

2

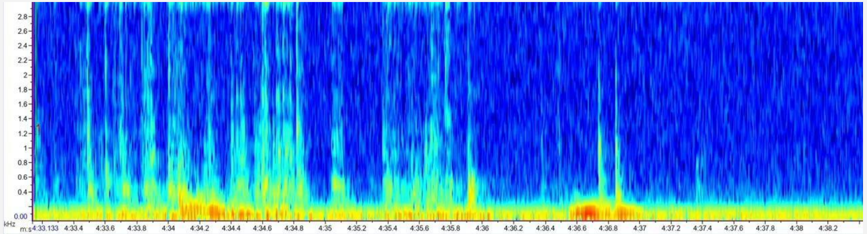

4

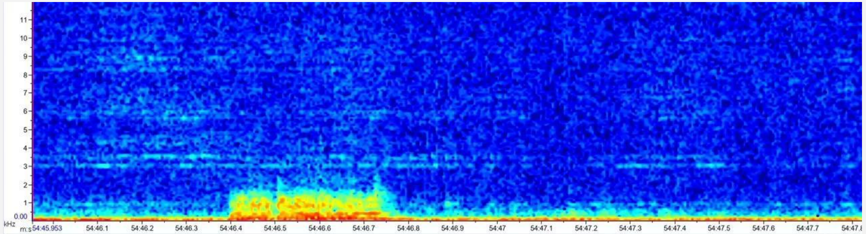

3

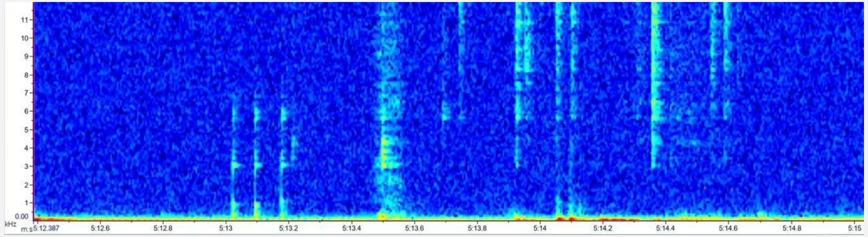

5

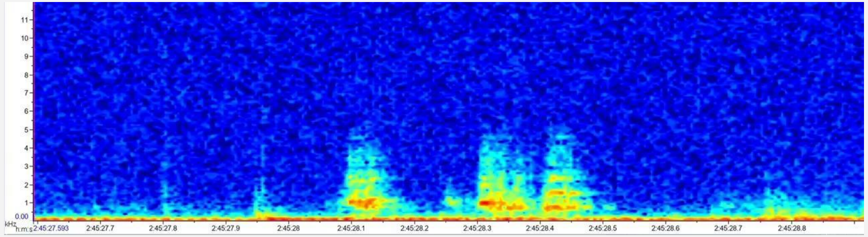

< Turtles tree

Next >

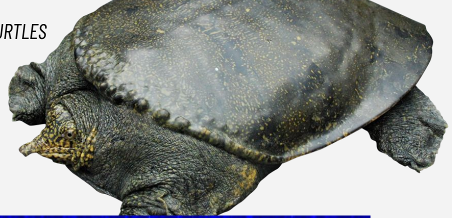

Trionychidae

Amyda ornata

1
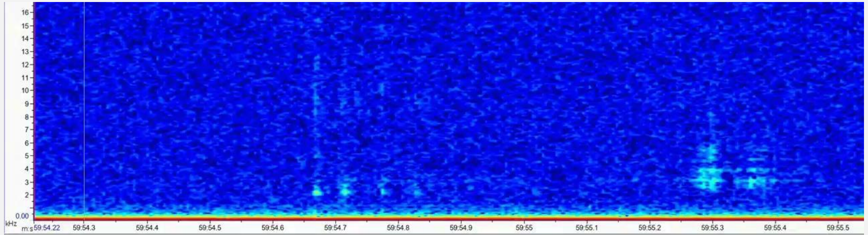

4
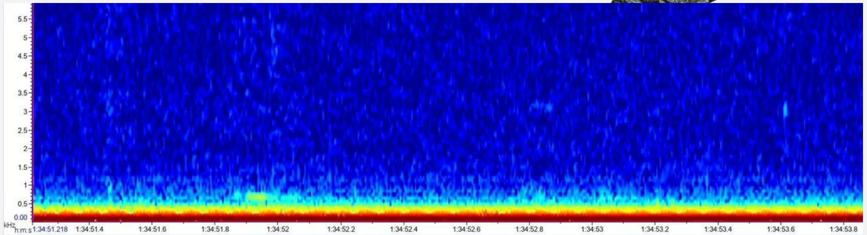

2
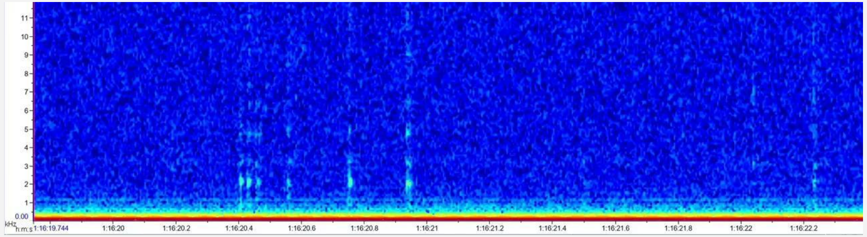

5
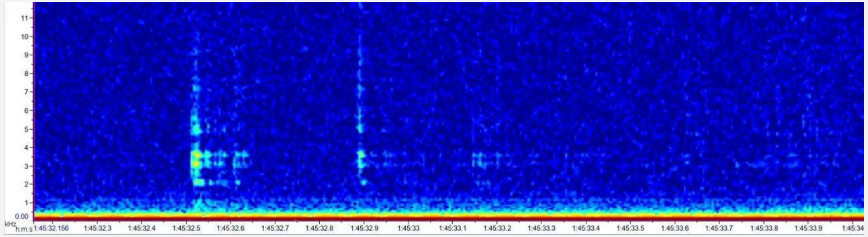

3
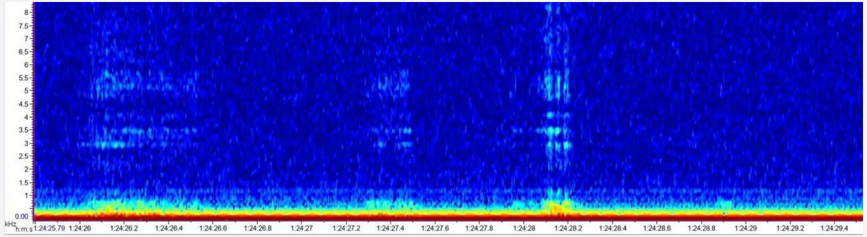

6
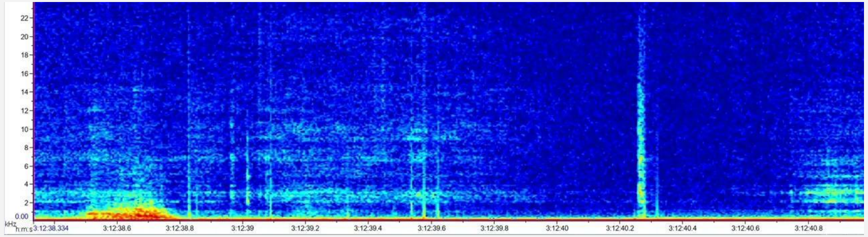

< Turtles tree

Next >

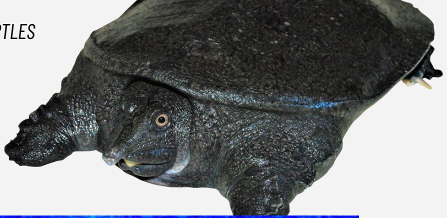

Trionychidae

Dogania subplana

1 2 3 4 5 6

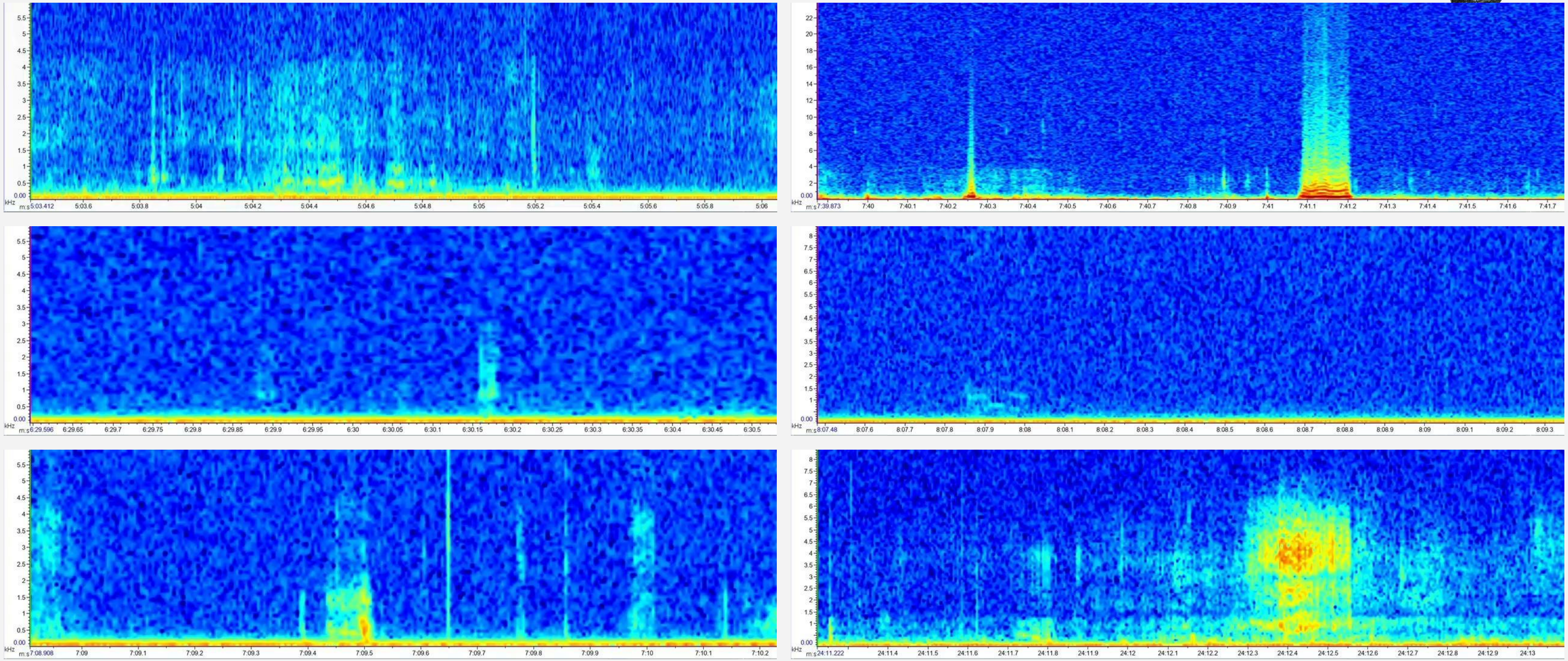

< Turtles tree

Next >

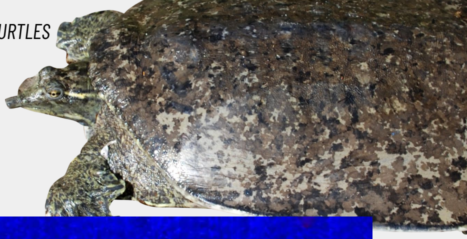

Trionychidae

Apalone spinifera

1
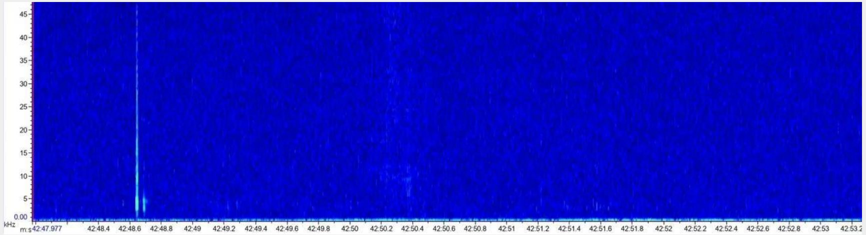

2
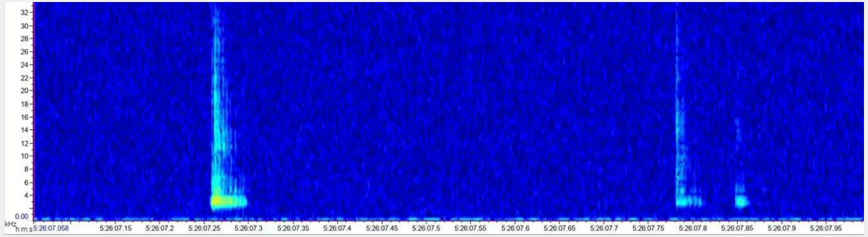

3
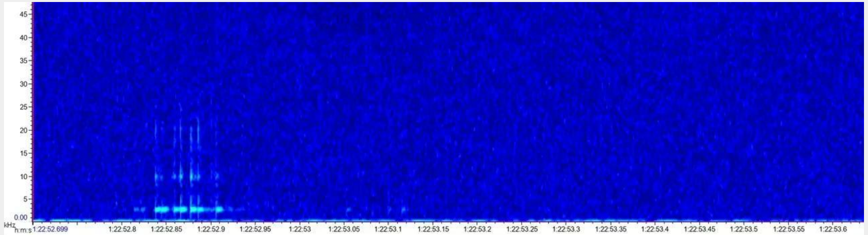

4
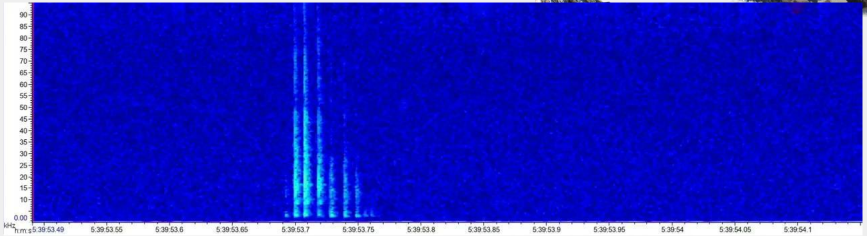

5
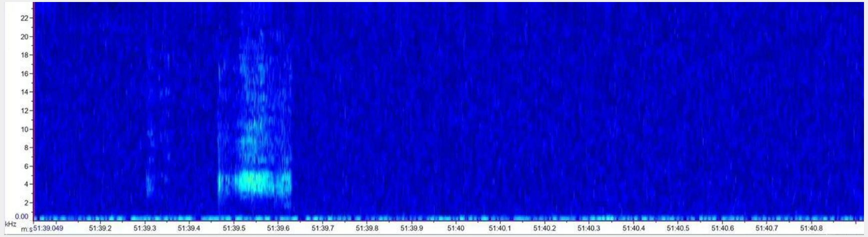

6
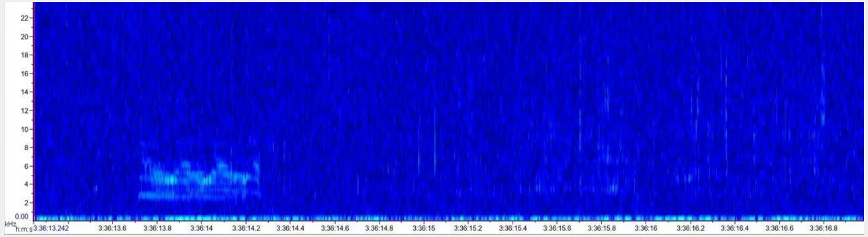

< Turtles tree

Next >

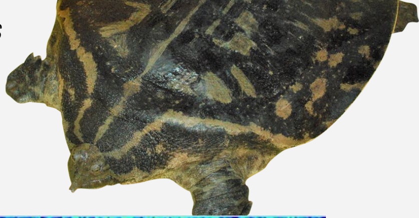

Trionychidae

Pelochelys bibroni

1
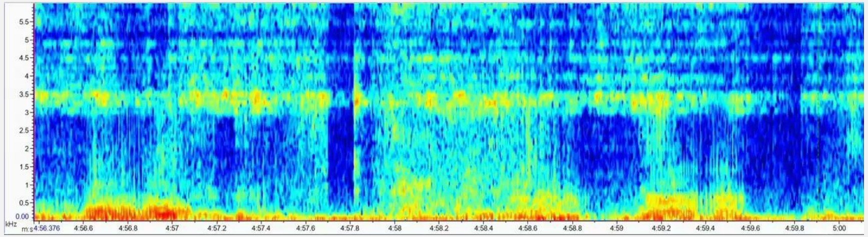

4
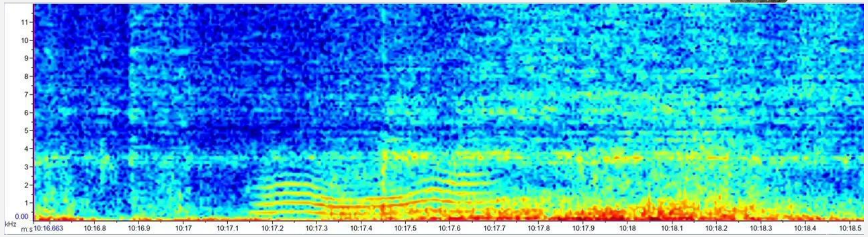

2
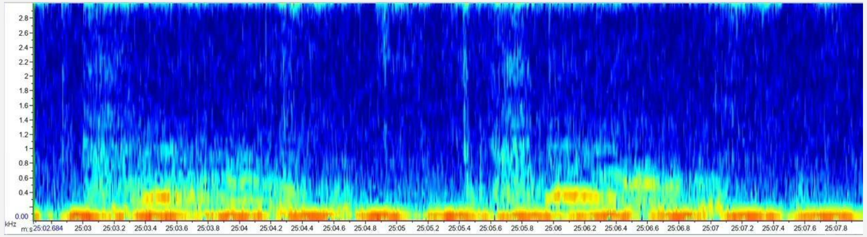

5
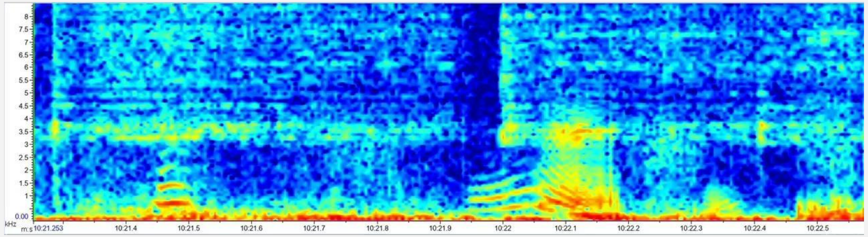

3
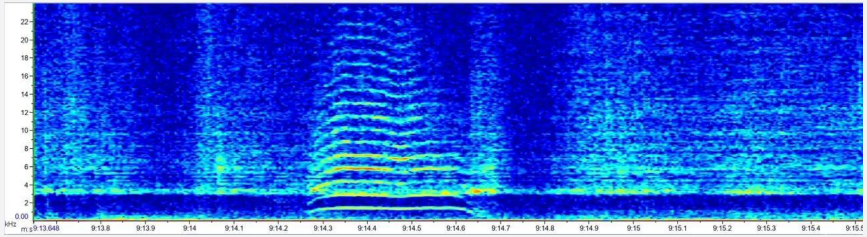

6
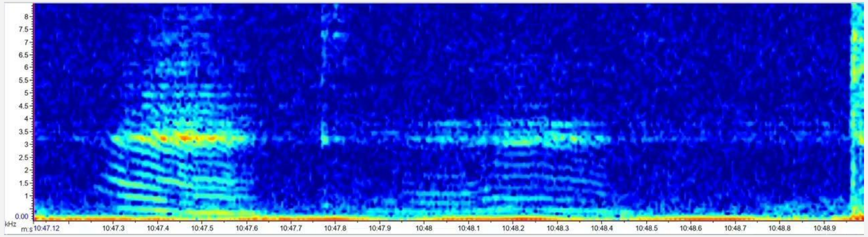

Trionychidae

Chitra indica

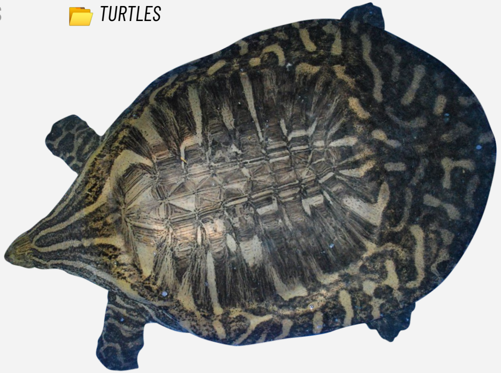

1
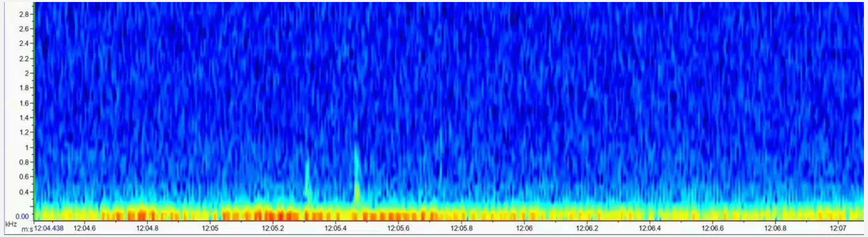

2
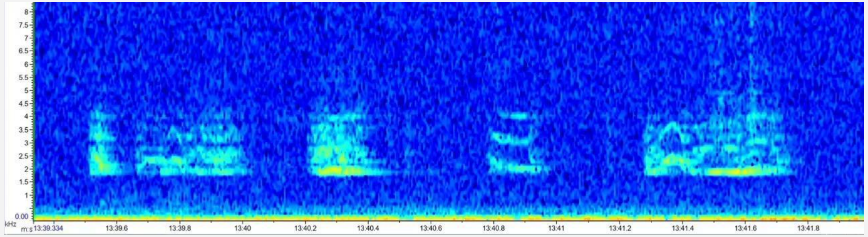

4
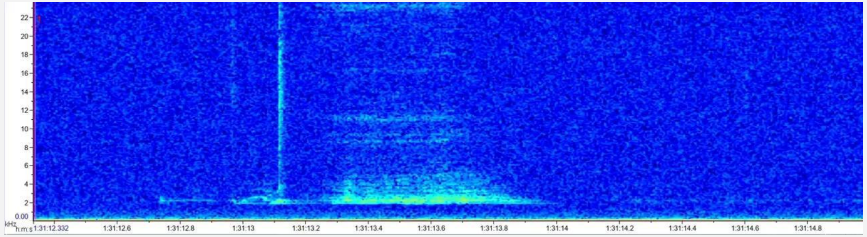

3
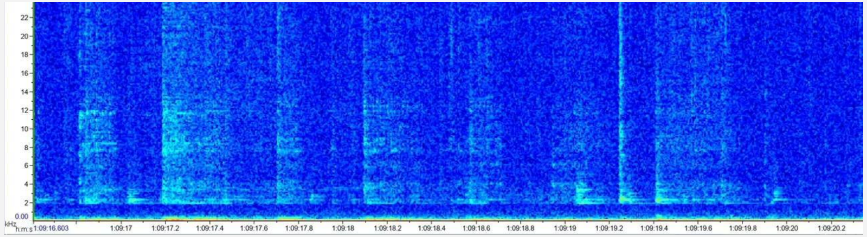

5
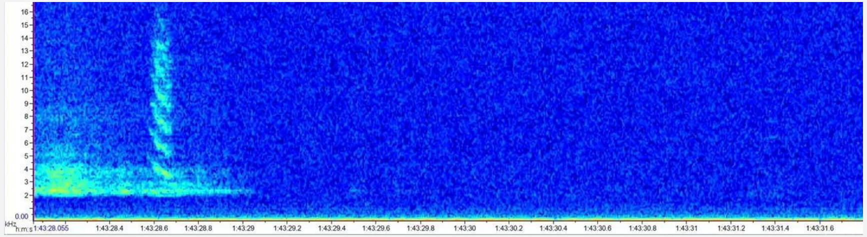

Carettochelyidae

Carettochelys insculpta

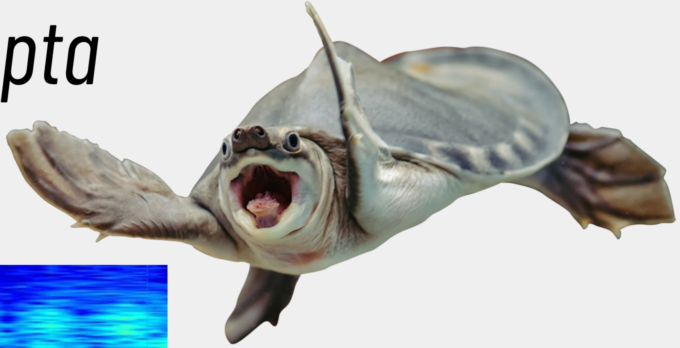

1

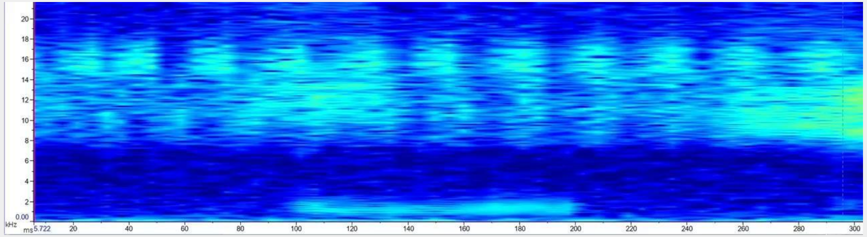

2

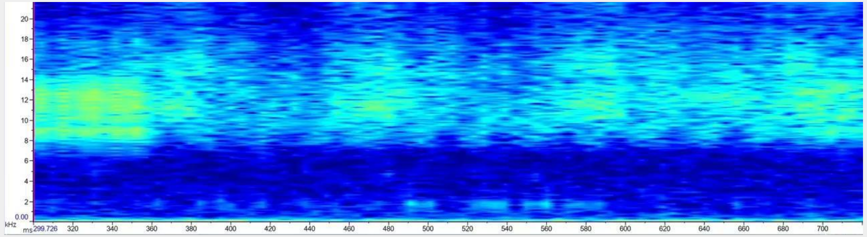

Kinosternidae

Kinosternon scorpioides

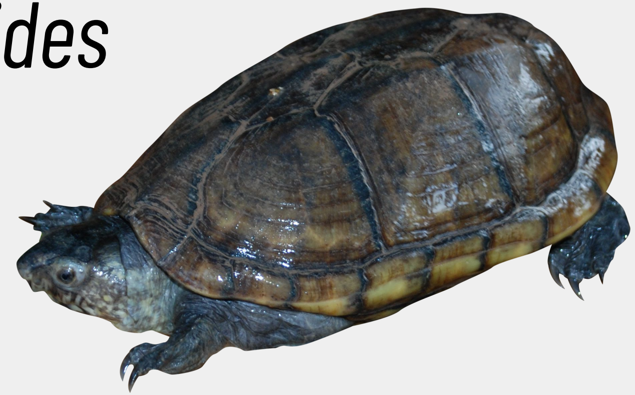

1

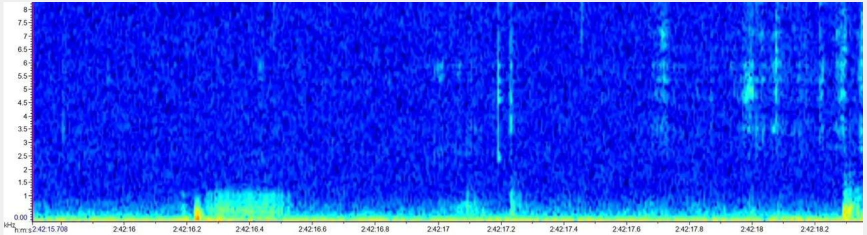

2

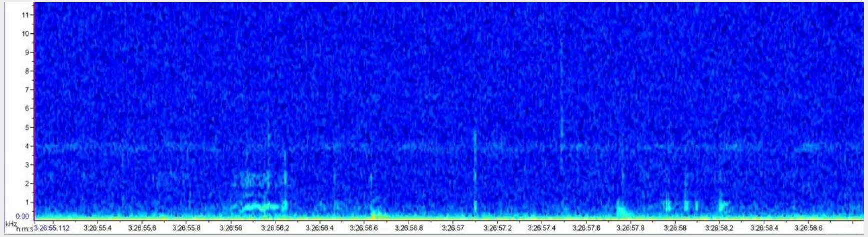

3

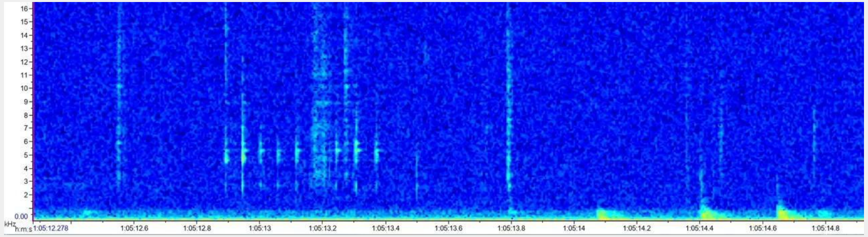

4

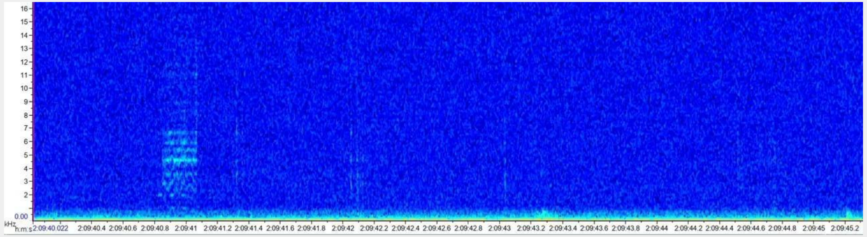

< *Turtles tree*

Next >

Kinosternidae

Sternotherus carinatus

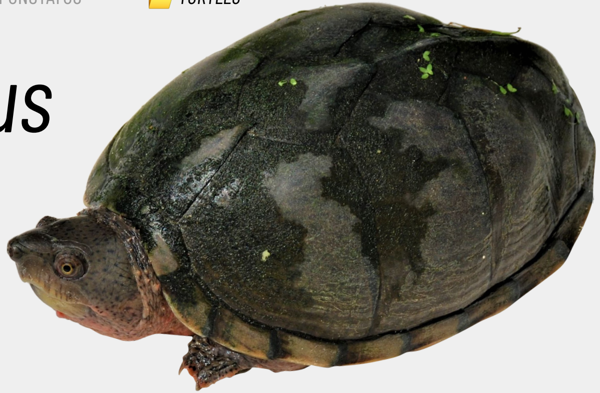

1

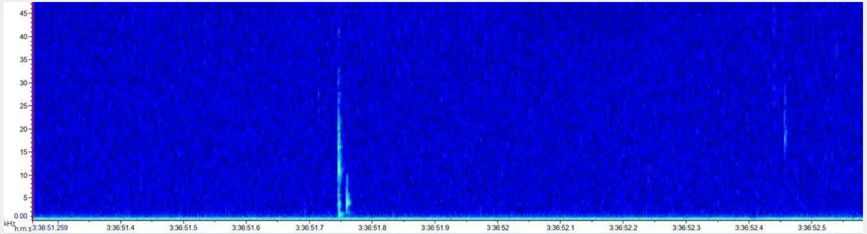

2

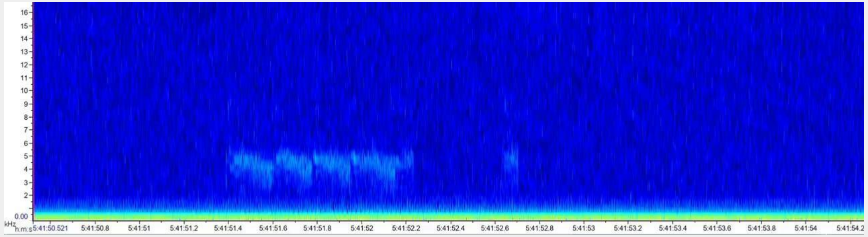

3

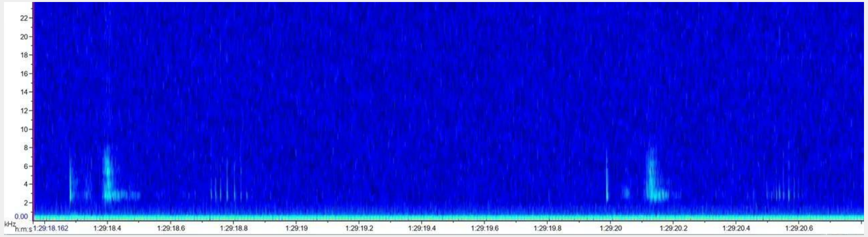

4

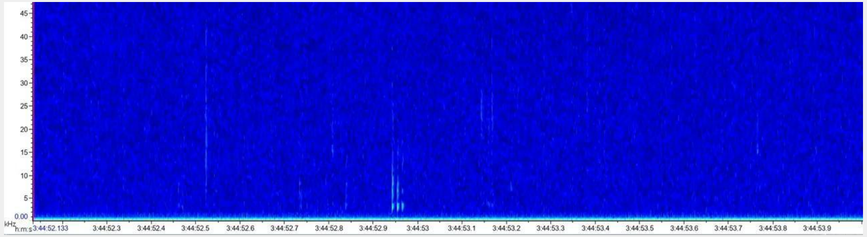

< Turtles tree

Next >

Kinosternidae

Claudius angustatus

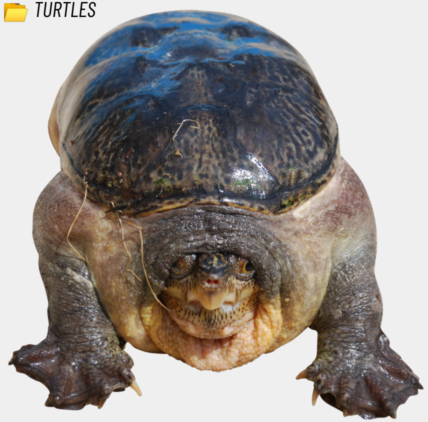

1

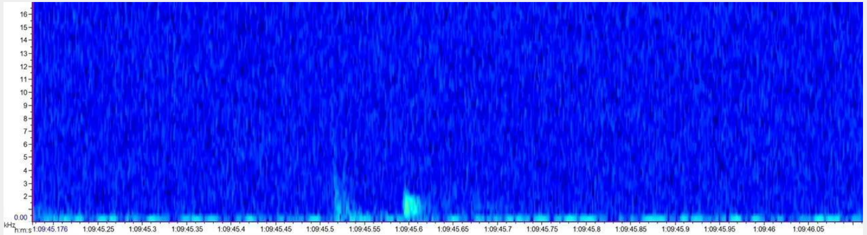

2

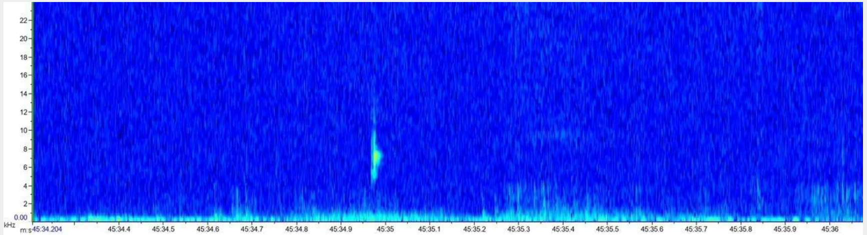

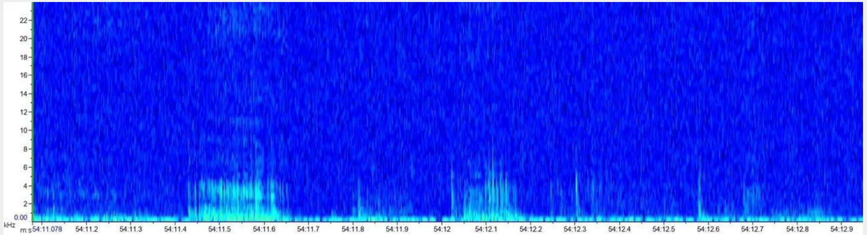

4

3

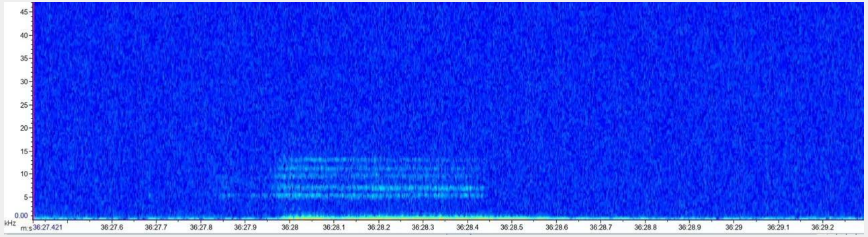

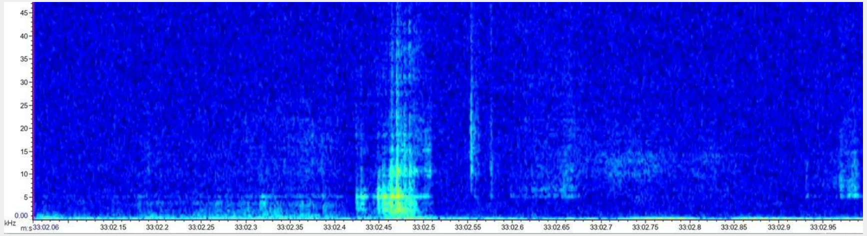

5

< Turtles tree

Next >

Dermatemydidae

Dermatemys mawii

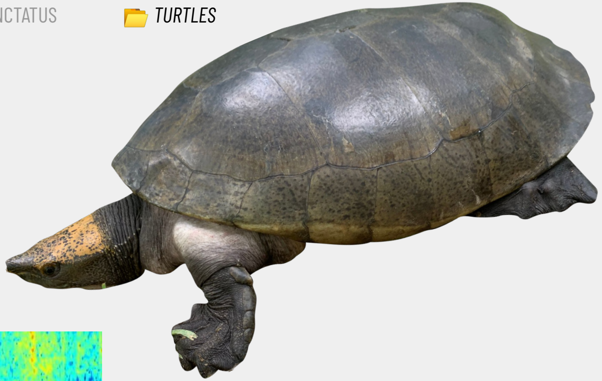

1

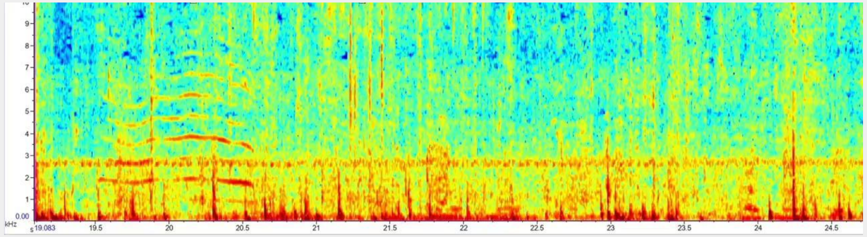

2

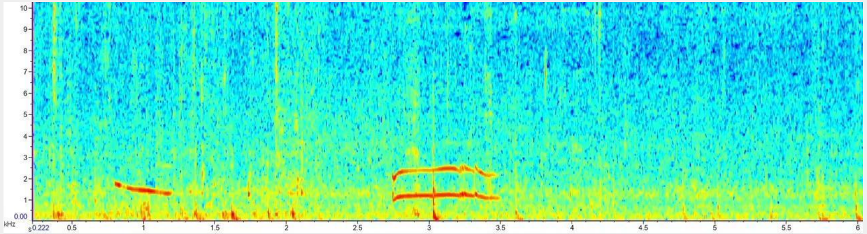

Chelydridae

Chelydra serpentina

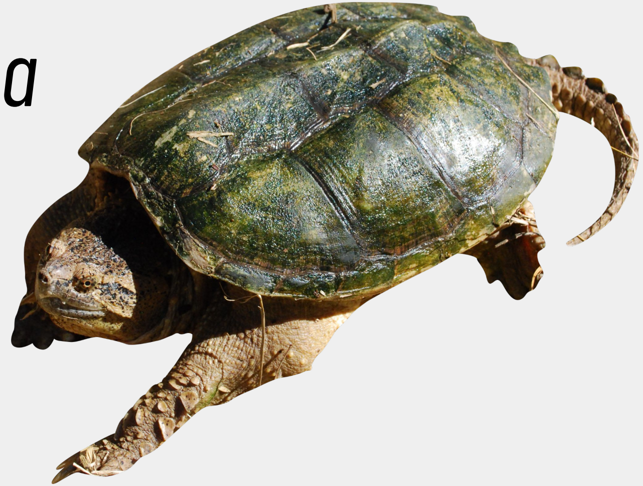

1

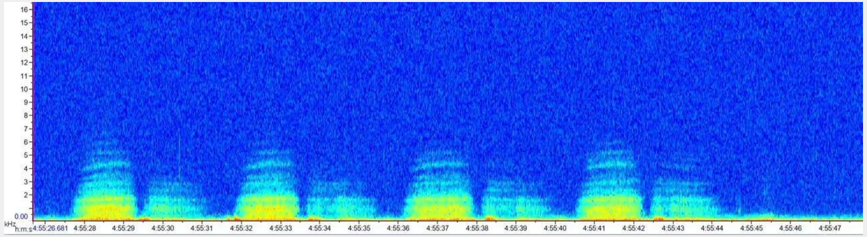

2

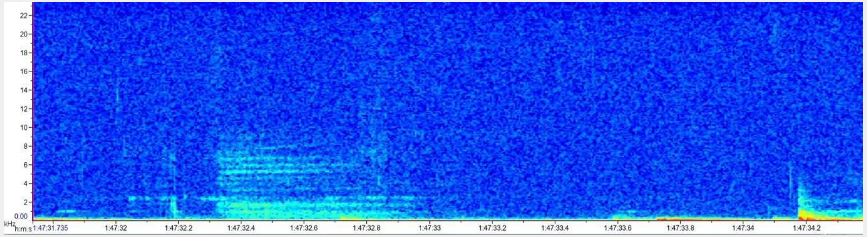

3

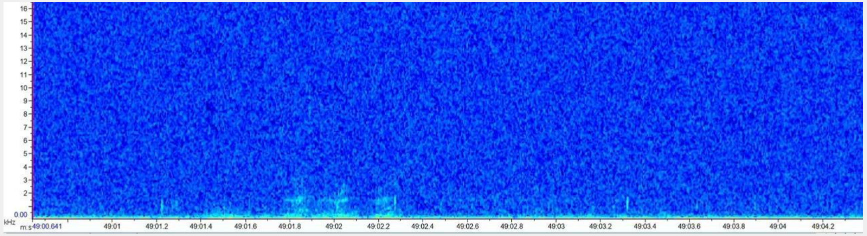

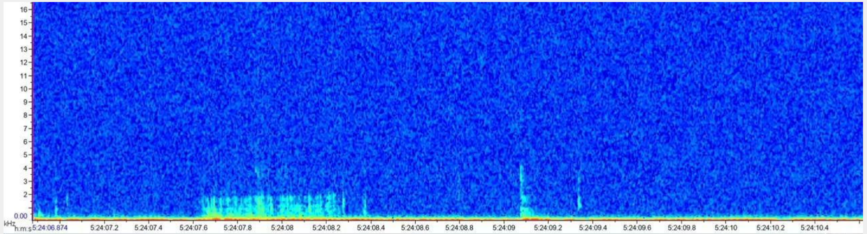

4

< Turtles tree

Next >

Chelydridae

Macrochelys temminckii

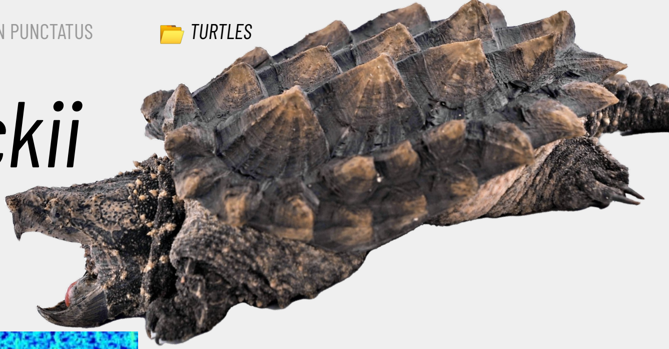

1

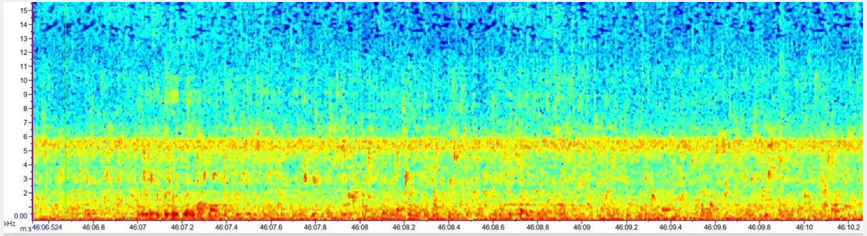

2

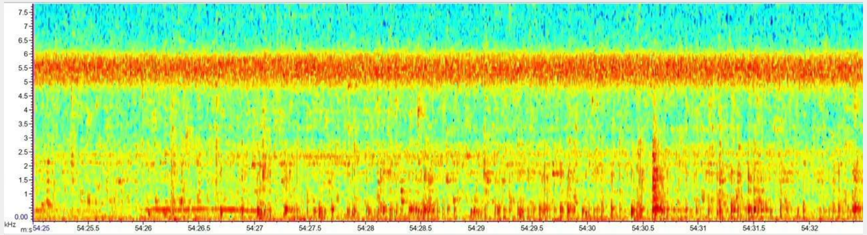

Cheloniidae

Natator depressus

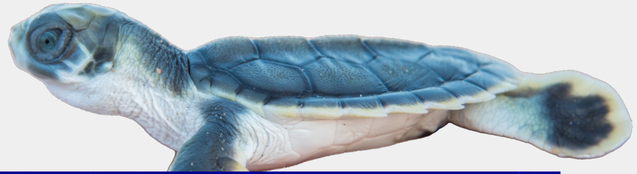

1
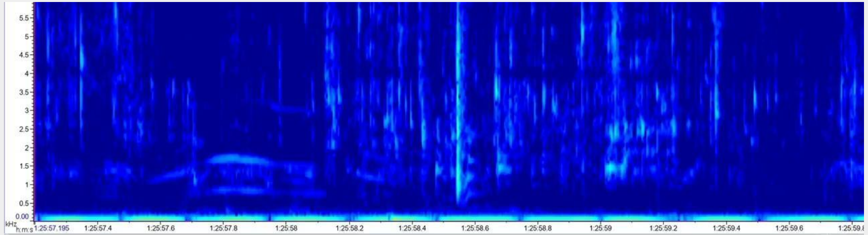

2
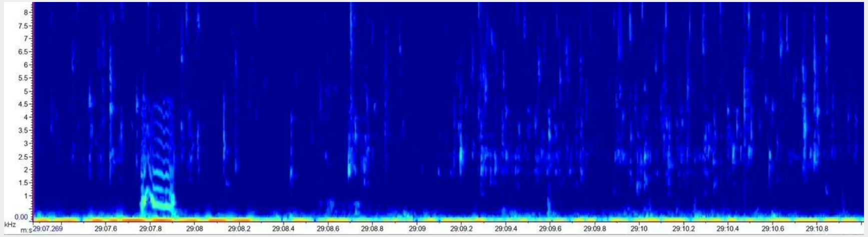

3
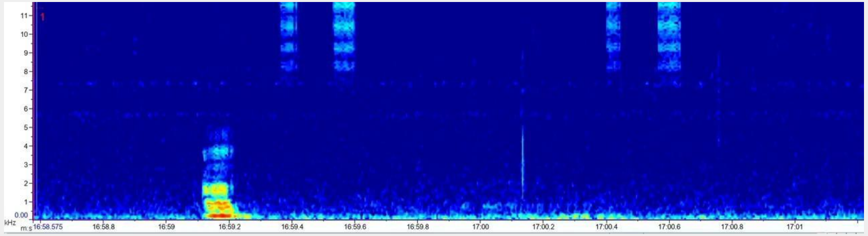

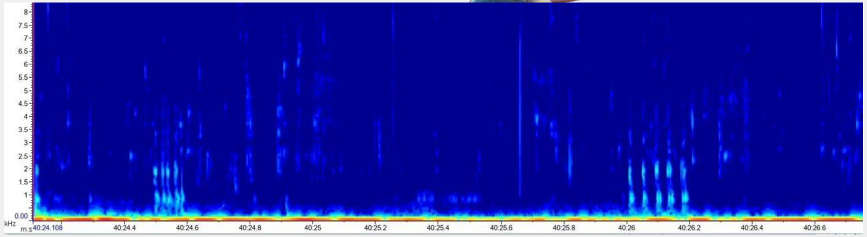
4

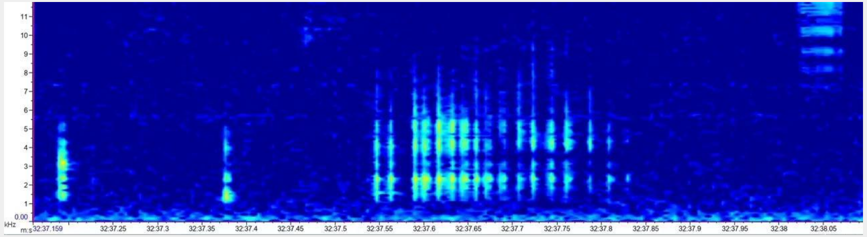
5

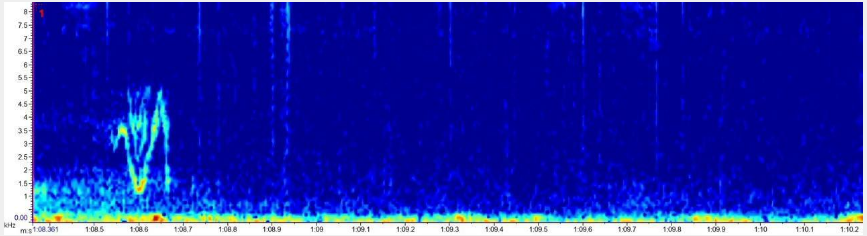
6

< Turtles tree

Next >

Cheloniidae

Lepidochelys kempii

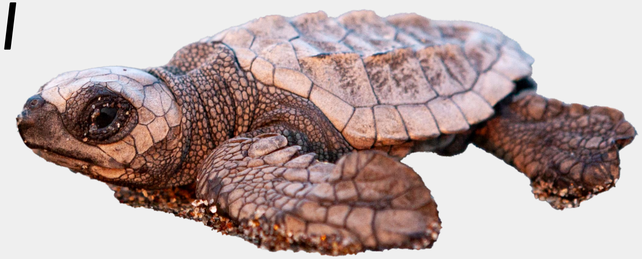

1

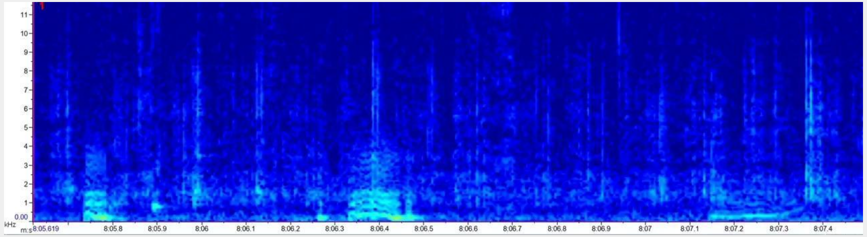

2

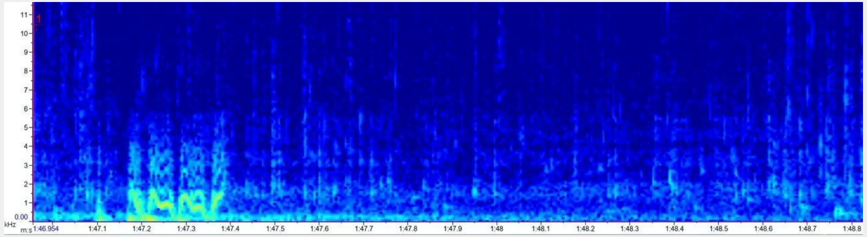

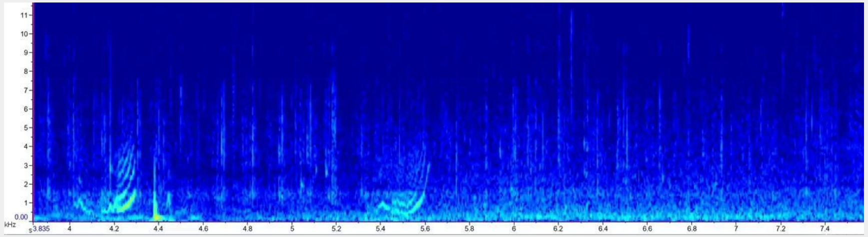

4

3

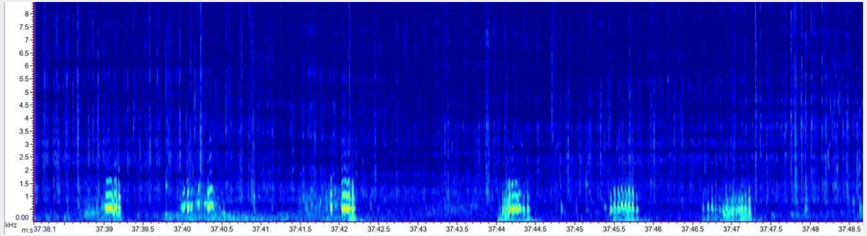

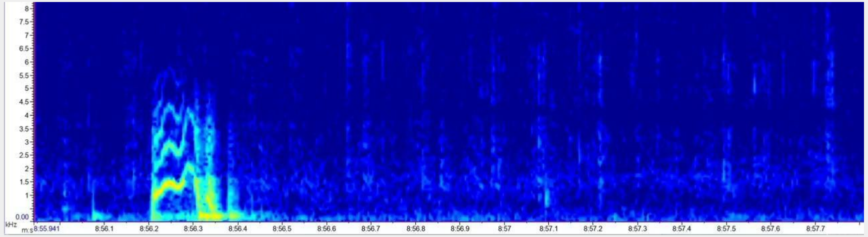

5

< Turtles tree

Next >

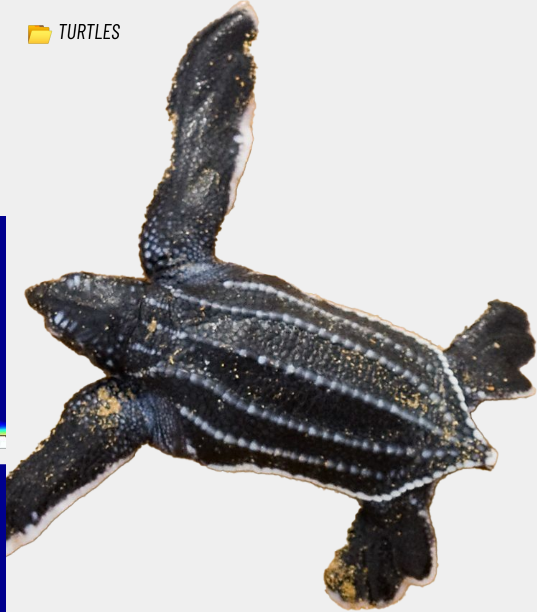

Dermochelyidae

Dermochelys coriacea

1

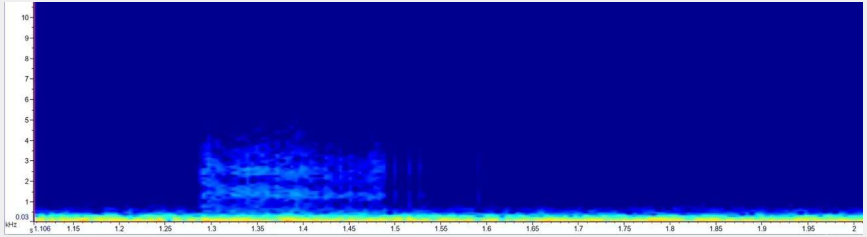

2

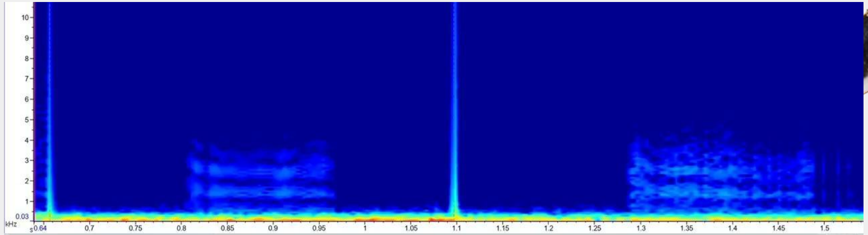

3

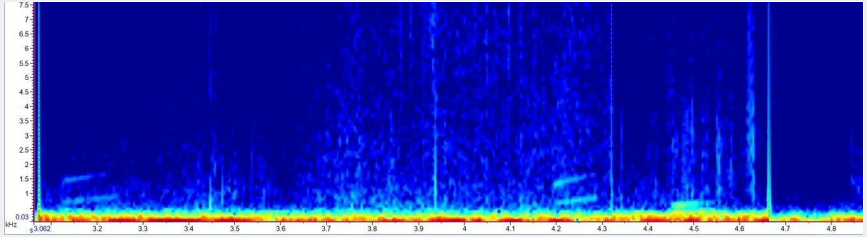

< Turtles tree

Next >

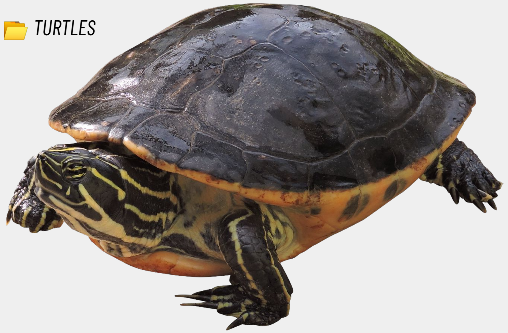

Emydidae

Pseudemys floridana

1

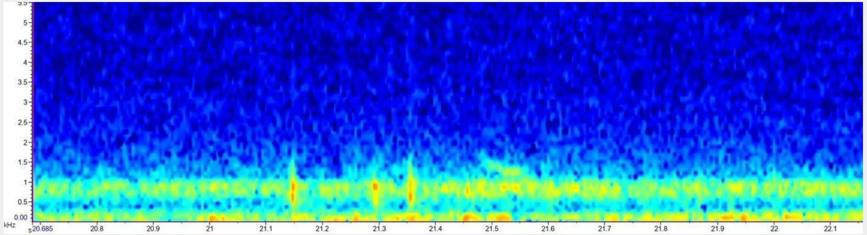

3

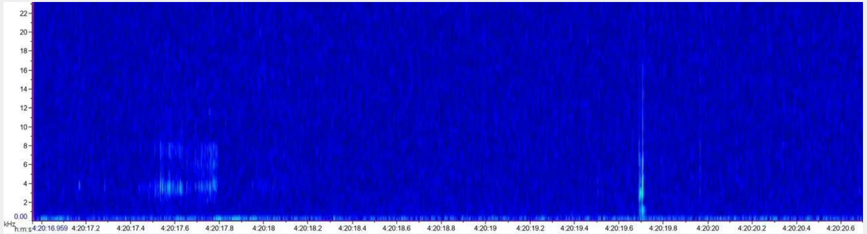

2

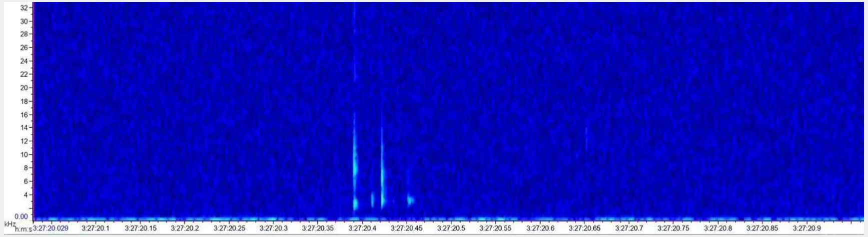

4

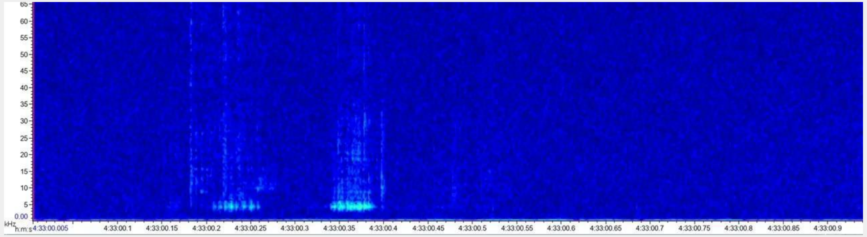

< Turtles tree

Next >

Emydidae

Chrysemys picta

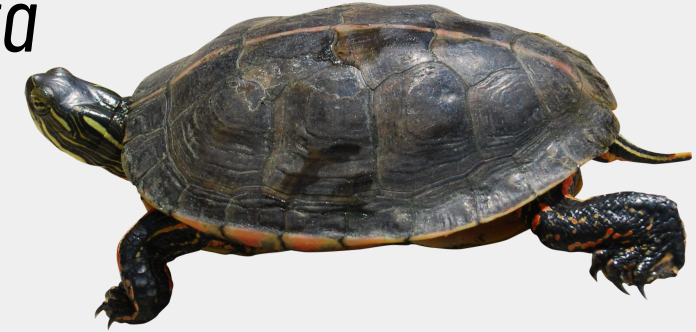

1

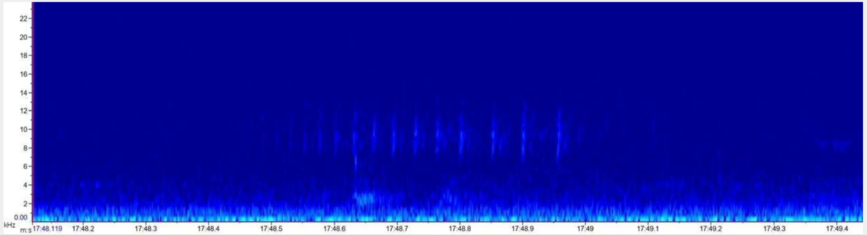

2

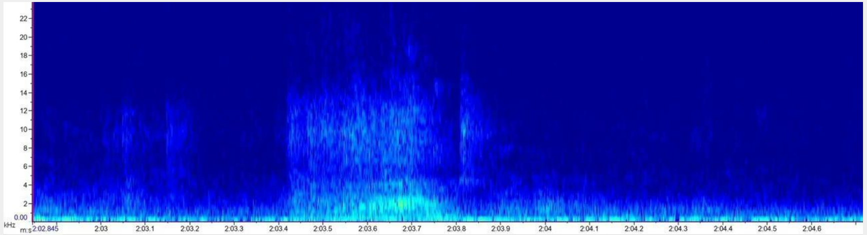

3

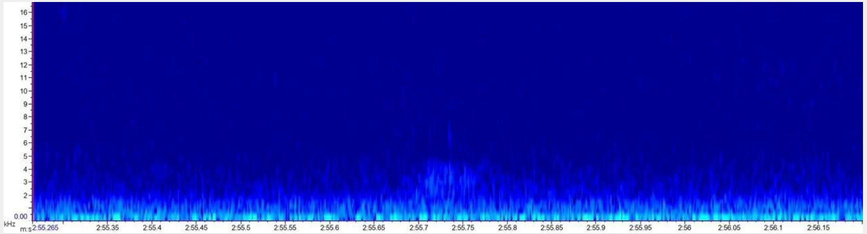

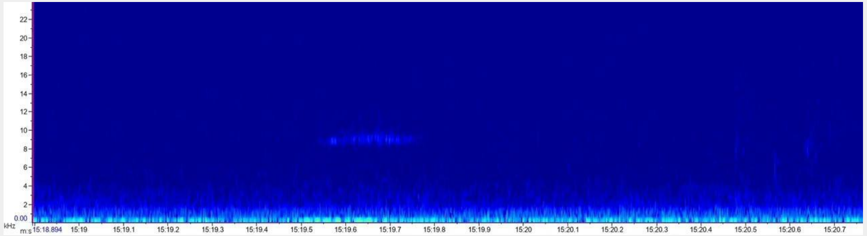

4

< Turtles tree

Next >

Emydidae

Graptemys sp.

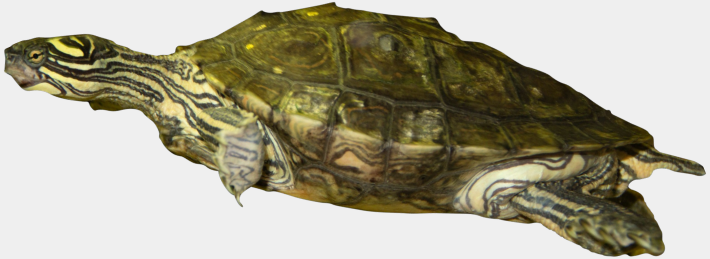

1

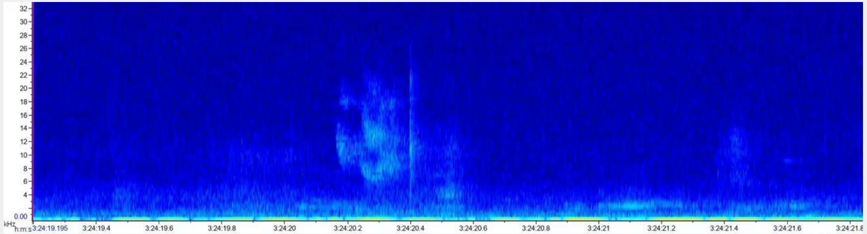

2

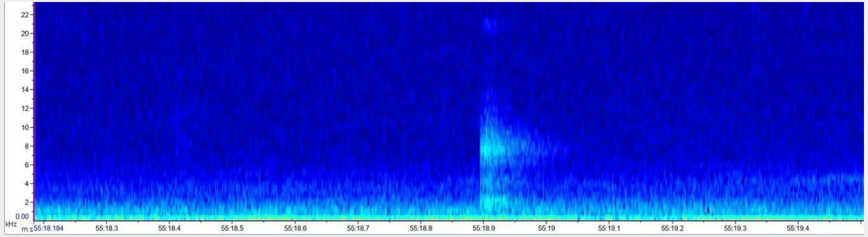

3

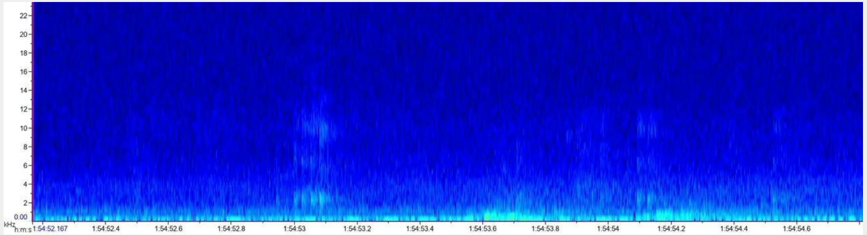

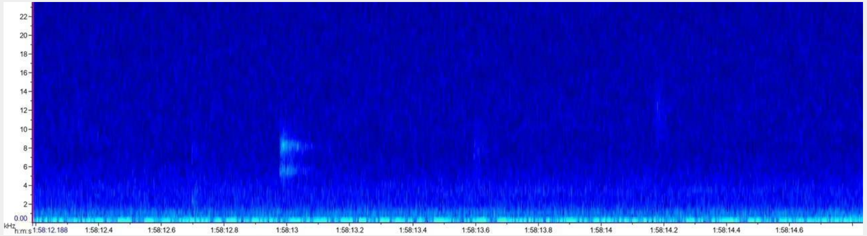

4

< Turtles tree

Next >

Emydidae

Trachemys dorbigni

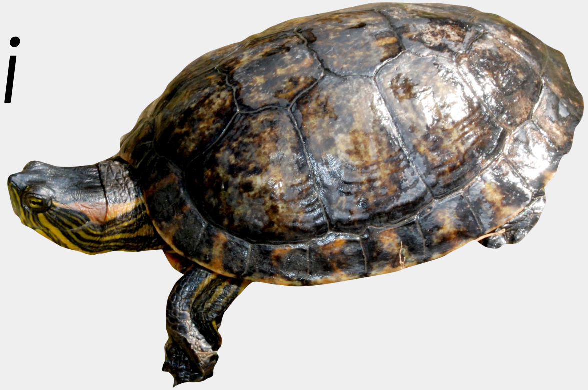

1

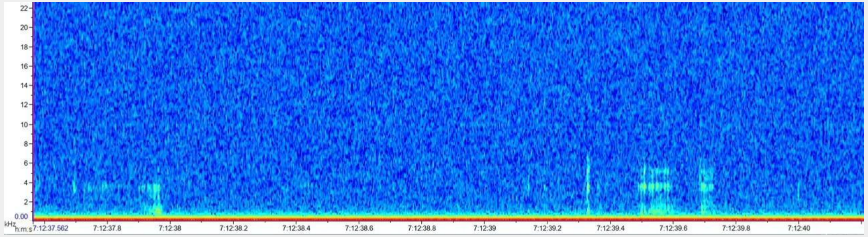

2

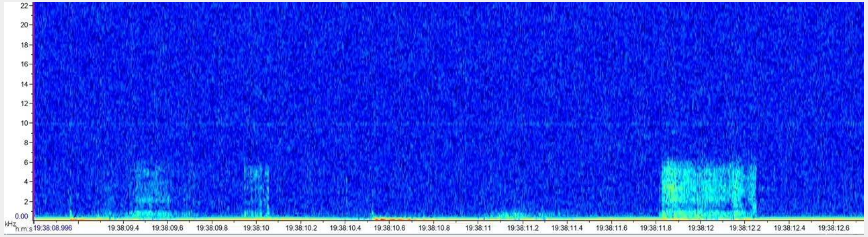

4

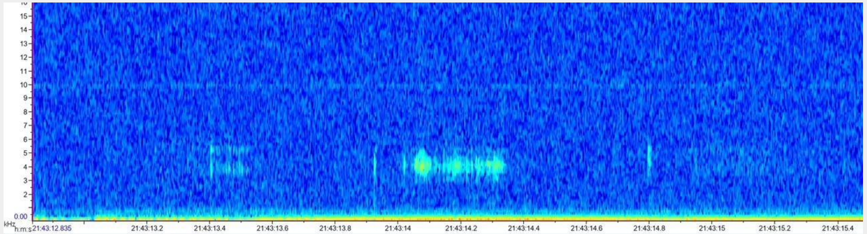

3

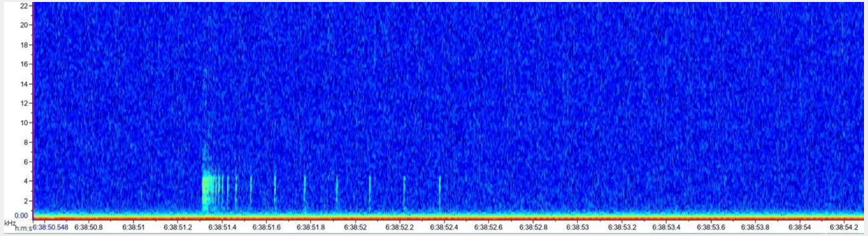

5

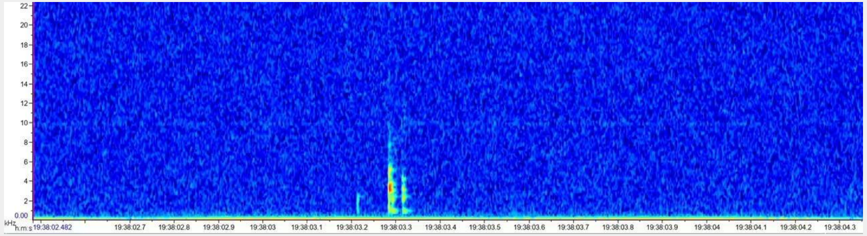

< Turtles tree Next >

Emydidae

Emydoidea blandingii

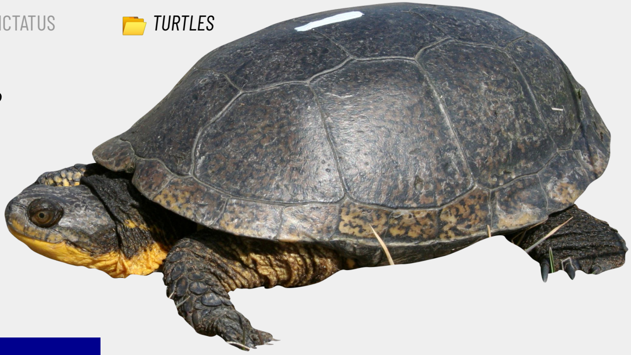

1

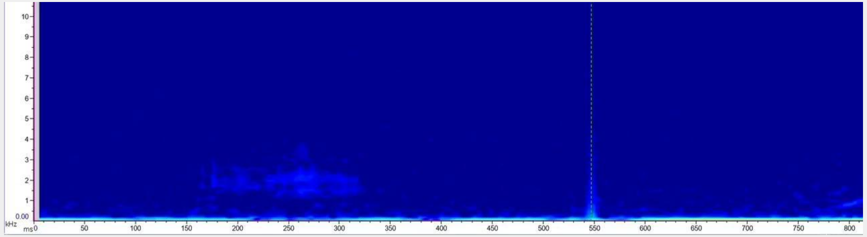

2

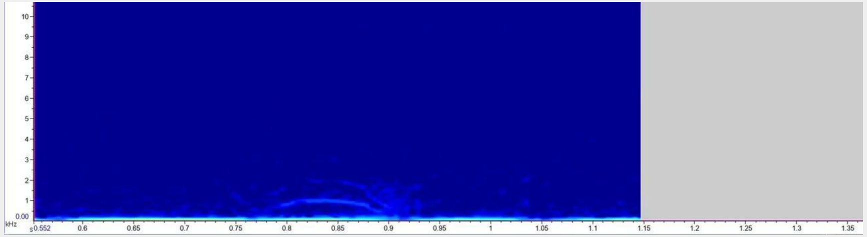

Emydidae

Emys orbicularis

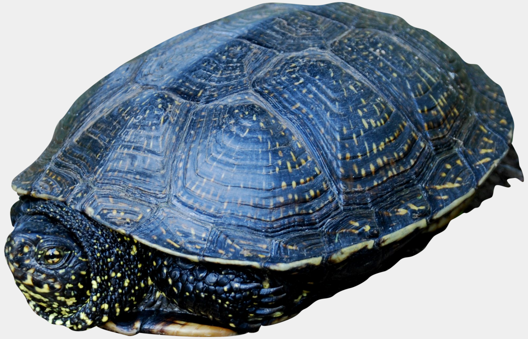

1

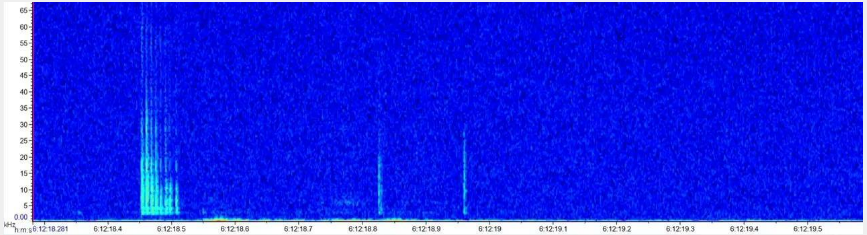

2

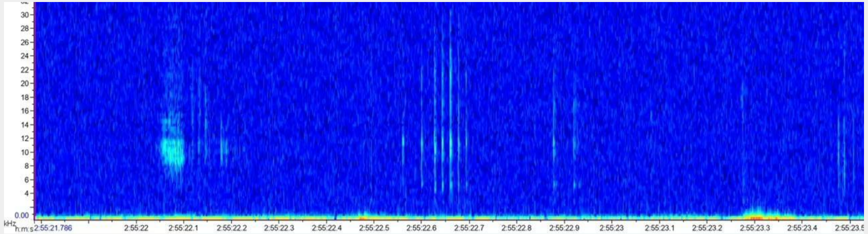

4

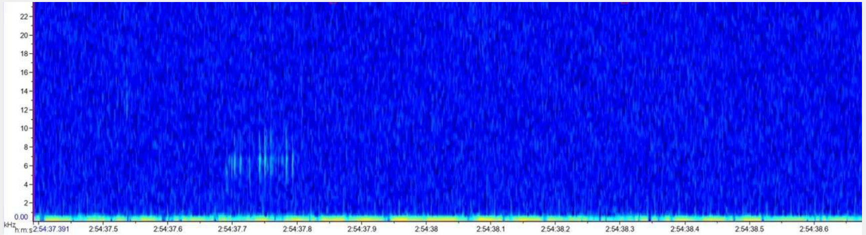

3

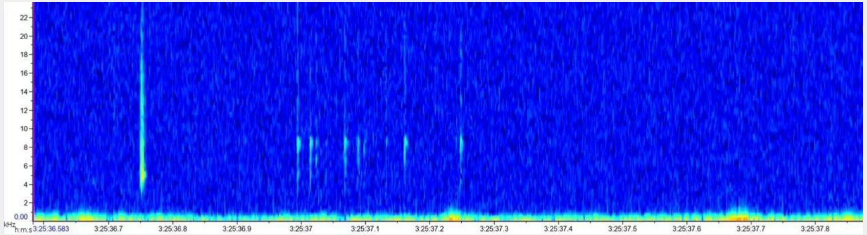

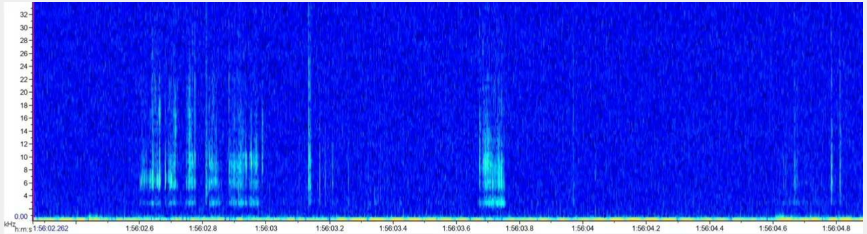

5

< Turtles tree

Next >

Testudinidae

Chelonoidis carbonaria

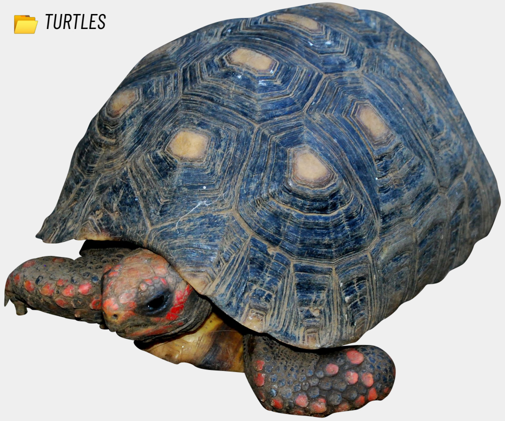

1

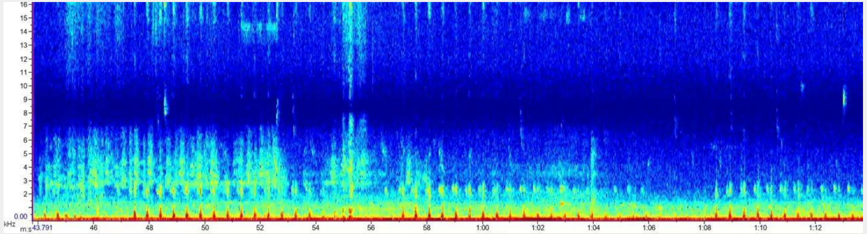

2

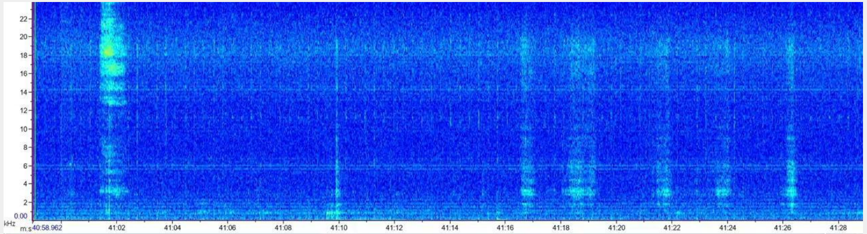

3

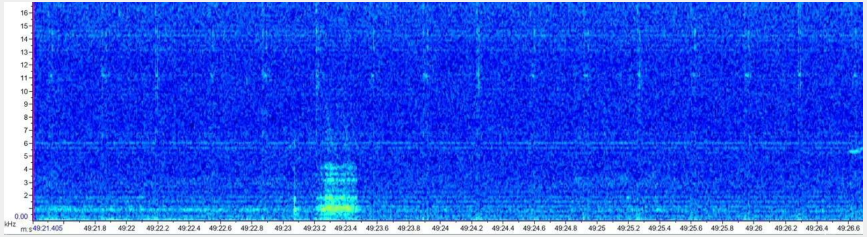

Geoemydidae

Geoemyda spengleri

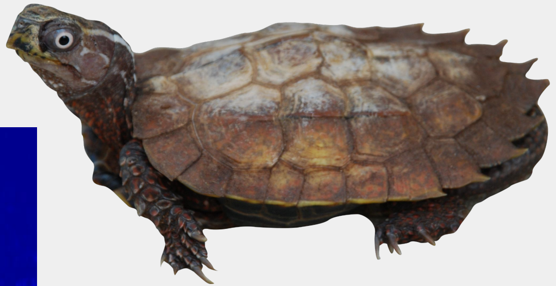

1

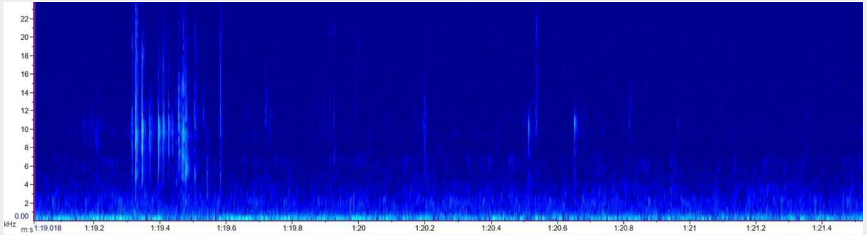

2

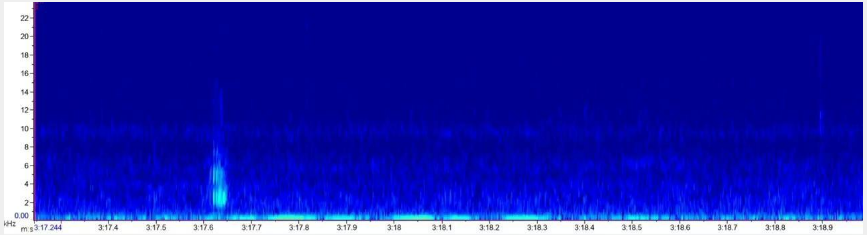

3

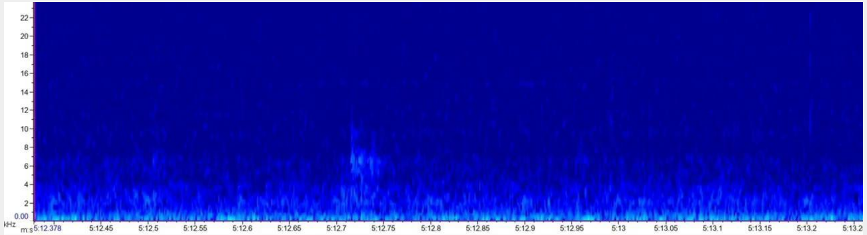

Geoemydidae

Malayemys macrocephala

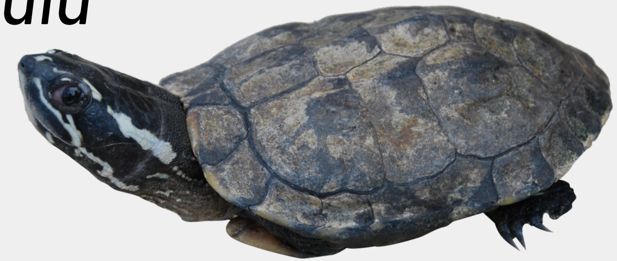

1

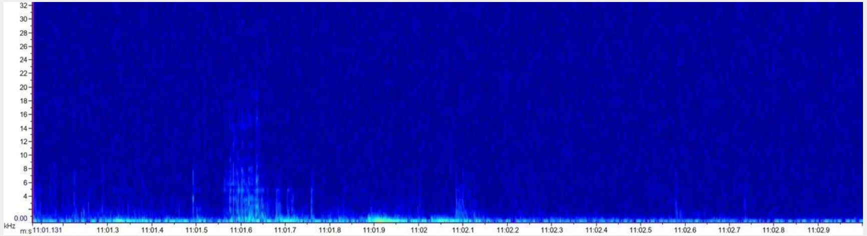

2

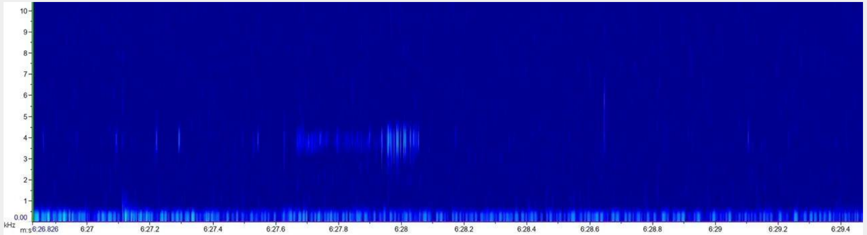

3

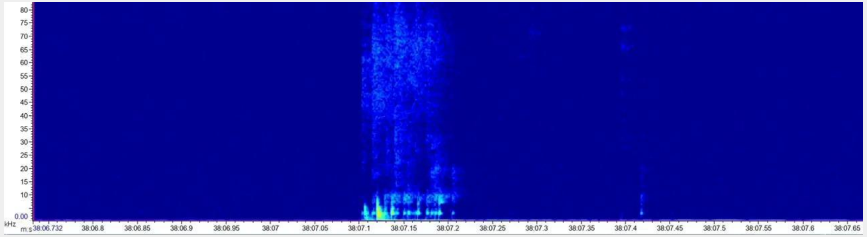

4

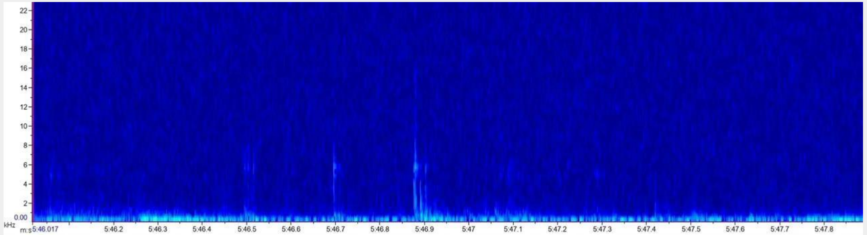

< Turtles tree

Next >

Geoemydidae

Heosemys spinosa

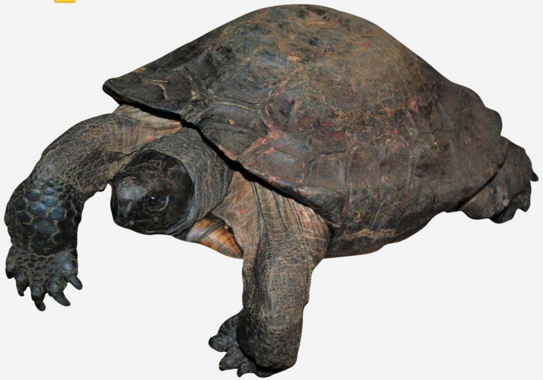

1
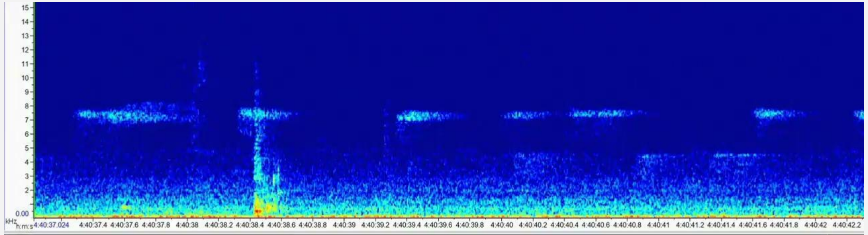

2
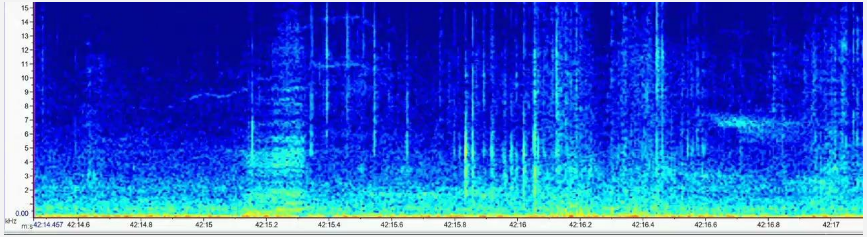

3
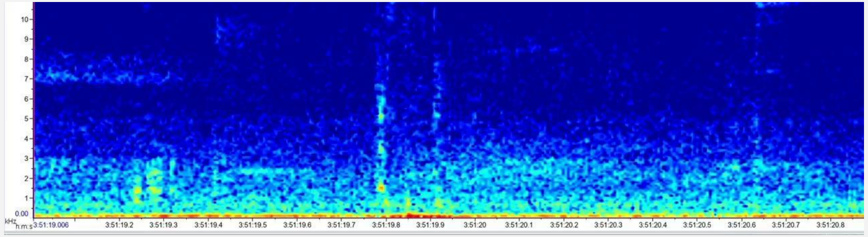

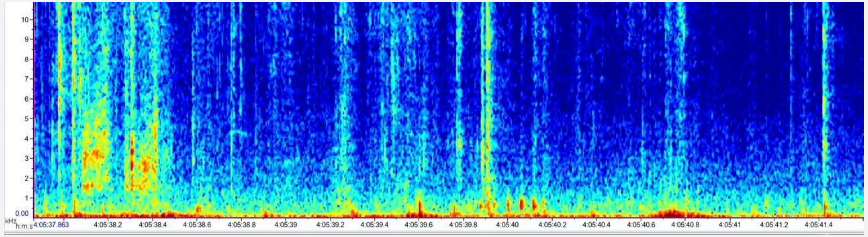
4

< Turtles tree

Next >

Geoemydidae

Cuora flavomarginata

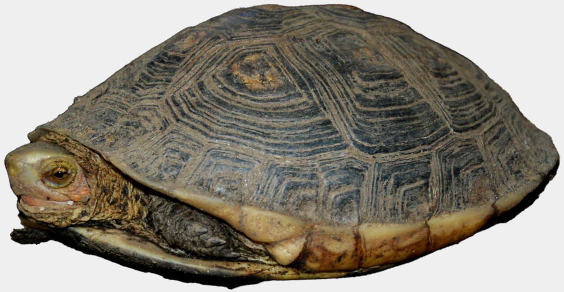

1

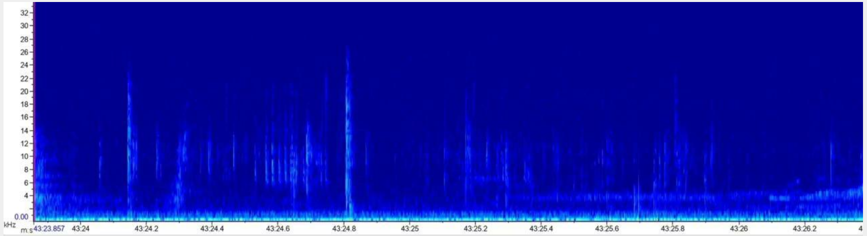

2

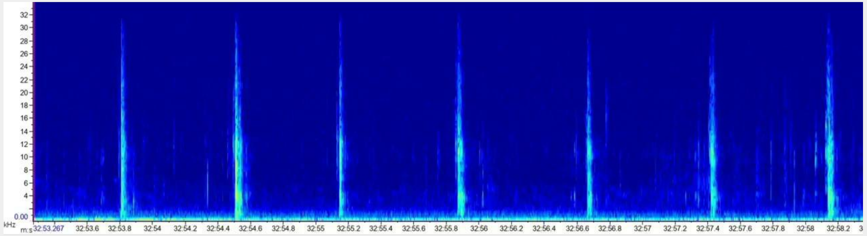

3

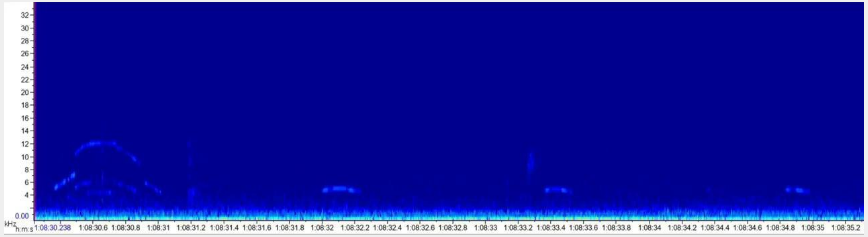

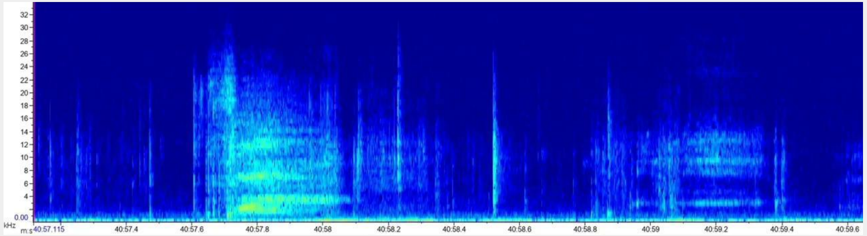

4

< Turtles tree

Next >

Geoemydidae

Rhinoclemmys punctularia

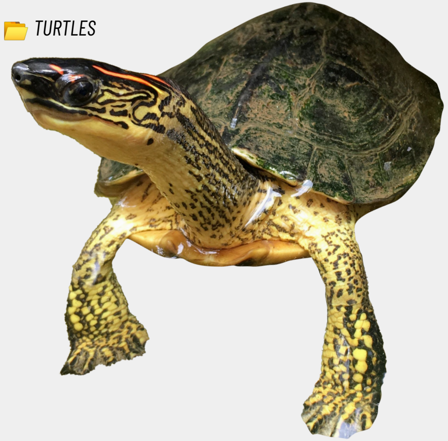

1

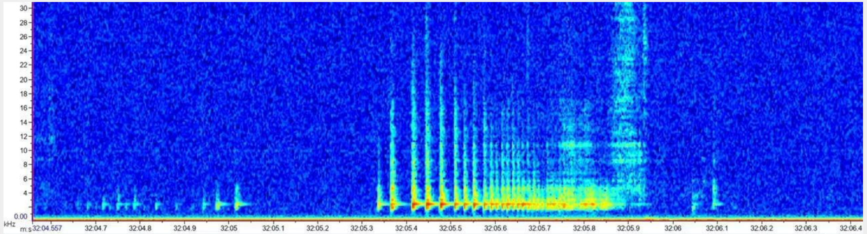

2

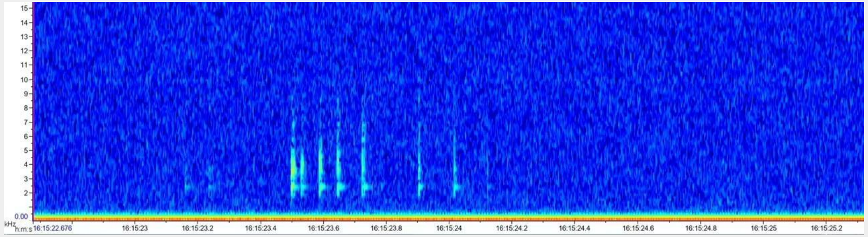

3

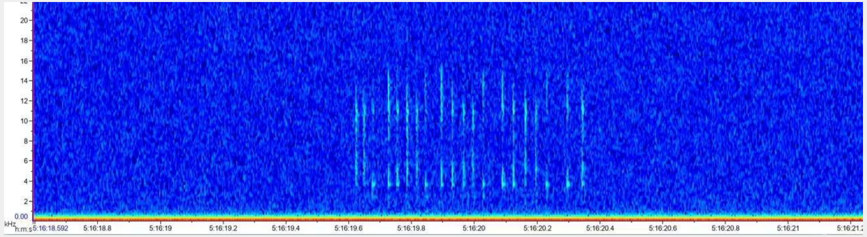

4

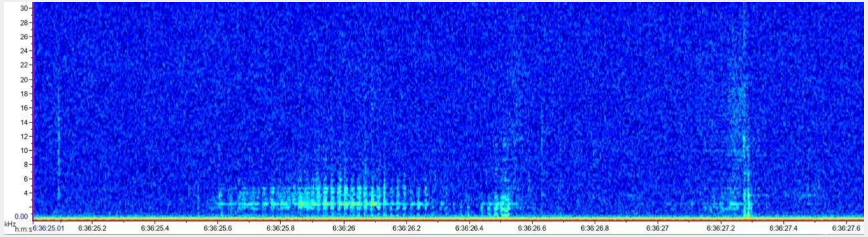

5

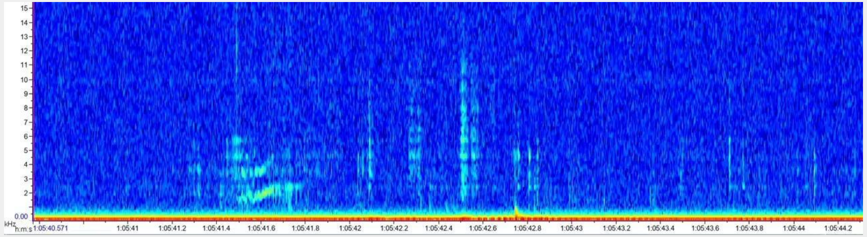

< Turtles tree

Next >

Geoemydidae

Rhinoclemmys pulcherrima

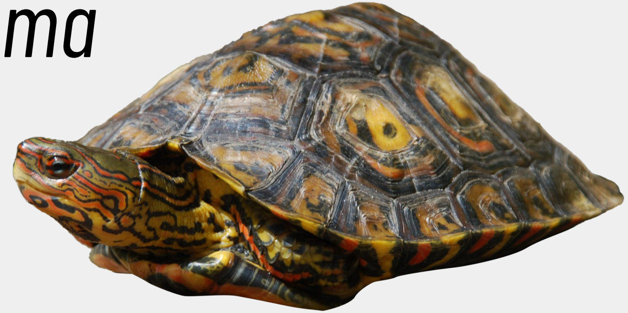

1

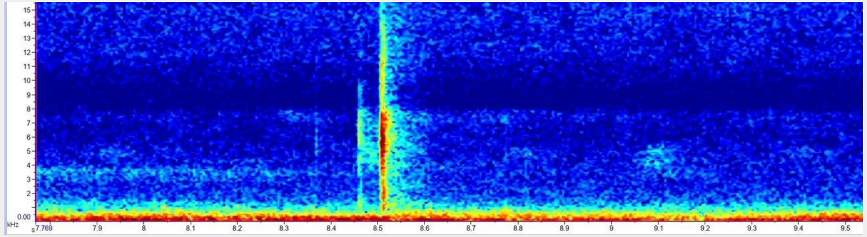

📁 HOME 📁 TYPHLONECTES COMPRESSICAUDA 📁 LEPIDOSIREN PARADOXA 📁 TURTLES
