# Supplementary material for: Common evolutionary origin of acoustic communication in choanate vertebrates
Source: Nat Commun. 2022 Oct 25;13:6089. doi: 10.1038/s41467-022-33741-8 (PMC9596459; doi:10.1038/s41467-022-33741-8)
Supplement: Supplementary file 10 — Reporting Summary [file 41467_2022_33741_MOESM10_ESM.pdf]

nature portfolio

Corresponding author(s): Gabriel Jorgewich Cohen
Marcelo Sánchez Villagra

Last updated by author(s): Sep 20, 2022

Reporting Summary

Nature Portfolio wishes to improve the reproducibility of the work that we publish. This form provides structure for consistency and transparency in reporting. For further information on Nature Portfolio policies, see our Editorial Policies and the Editorial Policy Checklist.

Statistics

For all statistical analyses, confirm that the following items are present in the figure legend, table legend, main text, or Methods section.

n/a	Confirmed	
☒	☐	The exact sample size (*n*) for each experimental group/condition, given as a discrete number and unit of measurement
☒	☐	A statement on whether measurements were taken from distinct samples or whether the same sample was measured repeatedly
☒	☐	The statistical test(s) used AND whether they are one- or two-sided<br>*Only common tests should be described solely by name; describe more complex techniques in the Methods section.*
☒	☐	A description of all covariates tested
☒	☐	A description of any assumptions or corrections, such as tests of normality and adjustment for multiple comparisons
☒	☐	A full description of the statistical parameters including central tendency (e.g. means) or other basic estimates (e.g. regression coefficient) AND variation (e.g. standard deviation) or associated estimates of uncertainty (e.g. confidence intervals)
☒	☐	For null hypothesis testing, the test statistic (e.g. *F*, *t*, *r*) with confidence intervals, effect sizes, degrees of freedom and *P* value noted<br>*Give P values as exact values whenever suitable.*
☒	☐	For Bayesian analysis, information on the choice of priors and Markov chain Monte Carlo settings
☒	☐	For hierarchical and complex designs, identification of the appropriate level for tests and full reporting of outcomes
☒	☐	Estimates of effect sizes (e.g. Cohen's *d*, Pearson's *r*), indicating how they were calculated

Our web collection on statistics for biologists contains articles on many of the points above.

Software and code

Policy information about availability of computer code

Data collection	No software was used during data collection
Data analysis	Sound analysis was conducted using Raven Pro 1.6 (Cornell Lab of Ornithology, Ithica, NY) and Praat 6.2.14 (Boersma, 2006). Further analyses were conducted using R (R core team 2018) with the package Ape 5.6-2 (Paradis et al., 2004).

For manuscripts utilizing custom algorithms or software that are central to the research but not yet described in published literature, software must be made available to editors and reviewers. We strongly encourage code deposition in a community repository (e.g. GitHub). See the Nature Portfolio guidelines for submitting code & software for further information.

Data

Policy information about availability of data

All manuscripts must include a data availability statement. This statement should provide the following information, where applicable:

- Accession codes, unique identifiers, or web links for publicly available datasets
- A description of any restrictions on data availability
- For clinical datasets or third party data, please ensure that the statement adheres to our policy

The authors declare that the data supporting the findings of this study are available within the paper and/or its supplementary information files. The literature search was conducted through the platforms Web of Science (https://clarivate.com/webofsciencegroup/solutions/web-of-science/) and Google scholar (https://scholar.google.com/).

Human research participants

Policy information about studies involving human research participants and Sex and Gender in Research.

Reporting on sex and gender	Not Applicable
Population characteristics	Not applicable
Recruitment	Not applicable
Ethics oversight	Not applicable

Note that full information on the approval of the study protocol must also be provided in the manuscript.

Field-specific reporting

Please select the one below that is the best fit for your research. If you are not sure, read the appropriate sections before making your selection.

☐ Life sciences ☐ Behavioural & social sciences ☒ Ecological, evolutionary & environmental sciences

For a reference copy of the document with all sections, see nature.com/documents/nr-reporting-summary-flat.pdf

Ecological, evolutionary & environmental sciences study design

All studies must disclose on these points even when the disclosure is negative.

Study description	We present new evidence for 53 species of four major clades (turtles, tuatara, caecilian and lungfish) in the form of vocal recordings and contextual behavioural information accompanying sound production. This and a broad literature-based dataset evidence acoustic abilities in several groups previously considered non-vocal. Critically, phylogenetic analyses encompassing 1800 species of choanate vertebrates reconstructs acoustic communication as a homologous trait
Research sample	We recorded 53 species, that include 50 turtle species, one caecilian, one lungfish and tuataras, as we aimed for vertebrates commonly considered non-vocal. Data collectecion aimed to cover the broadest phylogenetic representation of such groups, but was opportunistic at some dregree. Some of the endangered representatives of the Geoemydaidae, for example, were not included because we could not get access to these animals. Apart from the vocalizations we recorded, most of the acoustic communication data used in this work originates from the dataset published by Chen & Wiens (2020), that includes 1799 tetrapod species (supplementary data 4). In addition, we searched for information on acoustic communication among groups that are often considered to be silent (i.e., Testudines, Lepidosauria, Gymnophiona, Caudata and some anuran species).
Sampling strategy	Each species was recorded for at least 24 h, capturing both day and night activity. We aimed for recording males and females in different life stages whenever specimens were available. We also recorded ambient sound without the presence of any animals in order to account for possible noise/interference.
Data collection	Data collection was made by Gabriel J. Cohen, Arthur V. Pinatti, Nicole Klein, Stephan ettmar, Camila Ferrara, Sabrina Menezes and Jaren Serrano. For underwater sound recordings, we used the OceanBase (developed by the Laboratory of Acoustic and Environment - University of Sao Paulo, in partnership with Bunin tech®), an acoustic recorder specifically designed for underwater noise monitoring. It has a sensitivity of -157 ± 2 dB rel 1V/uPa ± 2dB and frequency band of 5Hz - 90kHz. In-air recordings were conducted using a Tascam® recorder DR-100MKIII with sensitivity of -115.5 ± 0.5 dB rel 13 mV/uPa ± 4dB and frequency band of 5Hz - 96kHz. Recordings were made in captivity using plastic pools to ensure that all sounds were produced by the animals being recorded.
Timing and spatial scale	Data was collected opportunistically between March 2020 and March 2022. Data collection continued until every studied groups had a significantive representation (which was in some cases precluded by the dificulties in accessing some of these animals). Data collection lasted at least 24 for each species, in order to capture as much activity as possible in different times of the day. Sounds were recorded in a frequency range of 5Hz - 90kHz.
Data exclusions	No data was excluded from analysis.
Reproducibility	We made sure to make all the raw data available, together with the codes and input files used in this analysis. A clear description of all procedures is available in the manusctipt.
Randomization	Randomization is not relevant to our study as we included all groups of choanate vertebrate animals accordingly to their position in the cladogram.
Blinding	Blinding does not apply to this study as it's main goal was to investigate the presence of acoustic behaviour among different animal groups. We did, however, record the environment without the presence of any animals to control for ambient sounds that could be misleading.

Did the study involve field work? ☐ Yes ☒ No

Reporting for specific materials, systems and methods

We require information from authors about some types of materials, experimental systems and methods used in many studies. Here, indicate whether each material, system or method listed is relevant to your study. If you are not sure if a list item applies to your research, read the appropriate section before selecting a response.

Materials & experimental systems

n/a	Involved in the study
☒	☐ Antibodies
☒	☐ Eukaryotic cell lines
☒	☐ Palaeontology and archaeology
☐	☒ Animals and other organisms
☒	☐ Clinical data
☒	☐ Dual use research of concern

Methods

n/a	Involved in the study
☒	☐ ChIP-seq
☒	☐ Flow cytometry
☒	☐ MRI-based neuroimaging

Animals and other research organisms

Policy information about studies involving animals; ARRIVE guidelines recommended for reporting animal research, and Sex and Gender in Research

Laboratory animals	This study did not use laboratory animals.
Wild animals	Wild animals used in this research were already maintained in captivity at different institutions such as zoos and research centers. A detailed list of species (53) with information about their approximate age is available in the supplementary material.
Reporting on sex	Information on sex of specimens was collected when possible (visible external sexual dimorphism) and compiled in the supplemental material. This information was not used in any analysis of the present work, as its focus has an evolutionary perspective of large clades, making information about sex of specimens irrelevant.
Field-collected samples	We did not compile information about captive conditions such as plastic pools' size and distance between microphone-source, as this varied greatly during our data collection. Many of the species we recorded are rare, endangered and hard to access. For this reason, we had to visit numerous institutions with a variety of conditions to be able to compile an expressive dataset. We were careful to keep the animals in comfortable sized pools (which also varies depending on the species' size and habits).
Ethics oversight	This research is non invasive and only used animals from institutions that are approved animal keepers. Therefore, we did not require any ethics approval besides the ones conducted by the same institutions that provided the animals.

Note that full information on the approval of the study protocol must also be provided in the manuscript.
